# Proteome Analysis of the UVB-Resistant Marine Bacterium *Photobacterium angustum* S14

**DOI:** 10.1371/journal.pone.0042299

**Published:** 2012-08-01

**Authors:** Sabine Matallana-Surget, Fabien Joux, Ruddy Wattiez, Philippe Lebaron

**Affiliations:** 1 UPMC Univ Paris 06, UMR7621, Laboratoire d'Océanographie Microbienne, Observatoire Océanologique, Banyuls/mer, France; 2 CNRS, UMR7621, Laboratoire d'Océanographie Microbienne, Observatoire Océanologique, Banyuls/mer, France; 3 Department of Proteomics and Microbiology, Interdisciplinary Mass Spectrometry Center (CISMa), University of Mons, Mons, Belgium; Consejo Superior de Investigaciones Cientificas, Spain

## Abstract

The proteome of the marine bacterium *Photobacterium angustum* S14 was exposed to UVB and analyzed by the implementation of both the post-digest ICPL labeling method and 2D-DIGE technique using exponentially growing cells. A total of 40 and 23 proteins were quantified in all replicates using either the ICPL or 2D-DIGE methods, respectively. By combining both datasets from 8 biological replicates (4 biological replicates for each proteomics technique), 55 proteins were found to respond significantly to UVB radiation in *P. angustum*. A total of 8 UVB biomarkers of *P. angustum* were quantified in all replicates using both methods. Among them, the protein found to present the highest increase in abundance (almost a 3-fold change) was RecA, which is known to play a crucial role in the so-called recombinational repair process. We also observed a high number of antioxidants, transport proteins, metabolism-related proteins, transcription/translation regulators, chaperonins and proteases. We also discuss and compare the UVB response and global protein expression profiles obtained for two different marine bacteria with trophic lifestyles: the copiotroph *P.* angustum and oligotroph *Sphingopyxis alaskensis*.

## Introduction

Research on the environmental effects of solar ultraviolet radiation in both terrestrial and aquatic ecosystems was largely stimulated by the discovery of an area of ozone depletion over Antarctica in the 1980's, the so-called ‘ozone hole’. The primary consequence of this phenomenon is that an increased amount of UVB radiation (280–315 nm) reaches the Earth's surface in high- and middle-latitude regions, and this is likely to increase the exposure of organisms in surface waters to UVB radiation [1], [2]. The response of heterotrophic bacteria to UV radiation involves a highly organized series of intracellular events, enabling them to attenuate the damaging impact of UVB radiation. It is well known that UVB radiation causes widespread damage in cells, and it also modulates the expression of many genes. Unlike transcriptomic approaches, only a few quantitative proteomics studies have explored the impact of damaging solar radiation on microorganisms. For example, a quantitative proteomic approach with 2D gels was used to compare the proteomes of irradiated and unirradiated *Deinococcus radiodurans* bacteria to identify the mechanisms of their extreme radioresistance and DNA repair [3], [4]. In this study, only 21 proteins correctly identified by mass spectrometry showed significant changes under radiation; however, none of the proteins were known to be relevant for radioresistance, except for the single-stranded DNA-binding protein (SSB) and PprA [5]. Another study was performed on the cyanobacterium *Nostoc commune*, a desiccation-tolerant terrestrial cyanobacterium, and its protein expression map was analyzed during growth under continuous UVB radiation using 2D gels [6]. A total of 27 UVB-induced proteins were identified that were mainly involved in lipid and carbohydrate metabolism and regulatory pathways. This study supported the idea that short-term stress and acclimation responses to damaging radiation are two completely different and remarkably complex strategies [6]. Another study focused on the light adaptation of the abundant marine cyanobacterium *Prochlorococcus marinus* MED4; this was assessed by a quantitative proteomic approach using iTRAQ [7]. The microorganism was cultured under three light intensities (low, medium and high), and fifteen proteins were deemed to be significantly influenced by changes in light intensity, especially photosystem-related proteins, which were down-regulated, and stress-related chaperones, which were up-regulated in high light compared with low light treatment. Finally, a comprehensive study of the effects of solar radiation on the oligotrophic marine bacterium *Sphingopyxis alaskensis* was recently assessed using the iTRAQ method [8]. The key factors implicated in an adaptive response to solar radiation included DNA-binding proteins, proteins involved in the detoxification of toxic compounds (such as glyoxal and reactive oxygen species), iron sequestration proteins that minimize oxidative stress, chaperones, proteins involved in nitrogen-related metabolism, and transcriptional/translational regulators [8]. To determine the species-specific pathways involved in the UV response and fundamental biological processes commonly affected in microorganisms, there is still a need for more studies on damaging UV radiation using key model of marine bacteria.

*Photobacterium angustum* S14 (formerly *Vibrio angustum*) is a heterotrophic gram-negative bacterium with a copiotrophic lifestyle isolated from surface coastal waters in Botany Bay (Sydney), Australia, and its genome has been fully sequenced [9]. *P. angustum* has served as a model for diverse stress studies. It has been previously reported that in response to starvation, *P. angustum* becomes more resistant to a variety of stresses such as visible light, UV radiation, heavy metal exposure, and temperature shifts; this is a phenomenon referred to as cross-protection against secondary stresses [10]. Thus, this bacterium produces a highly orchestrated response to starvation and stress conditions [11]. Moreover, *P. angustum* was previously found to be resistant to long-term exposure to UVB radiation, and its high level of resistance was well-correlated with its ability to efficiently repair the UVB-induced photoproducts during UVB exposure without any carbon source or photoreactivating light [12]. *P. angustum* must have evolved mechanisms of efficient repair for UVB-induced photoproducts in artificial seawater lacking carbon sources. Moreover, it is noteworthy that *P. angustum* was previously found to accumulate very low levels of cyclobutane pyrimidine dimers (CPDs) when grown under simulated solar radiation, even though UVB reduced the growth rate [13]. These latter findings are the reasons why we decided to construct a quantitative proteomics map of UVB-treated *P. angustum* cells. We sought to provide a profile of the proteins present and the major processes that occur under exposure to UVB after a given dose of radiation. Thus, the objective of this study was to examine the UVB response of this bacterial model using both gel-free and gel-based quantitative proteomics approaches to maximize the coverage of quantified proteins.

In this study, we utilized two different quantitative proteomics methods to screen for key proteins regulated by UVB. As a gel-free approach, we used the recently developed post-digest Isotope Coded Protein Labeling (ICPL) method [14]. The post-digest ICPL method can be classified as a shotgun type of experiment or a gel-free mass spectrometry (MS)-based method. As a second quantitative proteomics approach, we used the common gel-based technology, the two-dimensional difference gel electrophoresis (2D-DIGE). Both approaches are amine-specific proteomic strategies. Post-digest ICPL labeling uses heavy and light isotopic reagents, while the 2D-DIGE method employs a pair of fluorescent CyDyes. While the relative protein quantification using the DIGE approach is based on a 2D-gel image analysis comparing spot volume ratios, the relative peptide quantification using ICPL is inferred from integrated MS/MS peak areas of the heavy and light versions of the ICPL-labeled peptides. In this study, we demonstrate that it is valuable to combine different proteomics methodologies to reveal the many cellular pathways affected by UVB radiation.

## Materials and Methods

### Bacterial growth conditions and UVB radiation treatment

**Figure 1:**
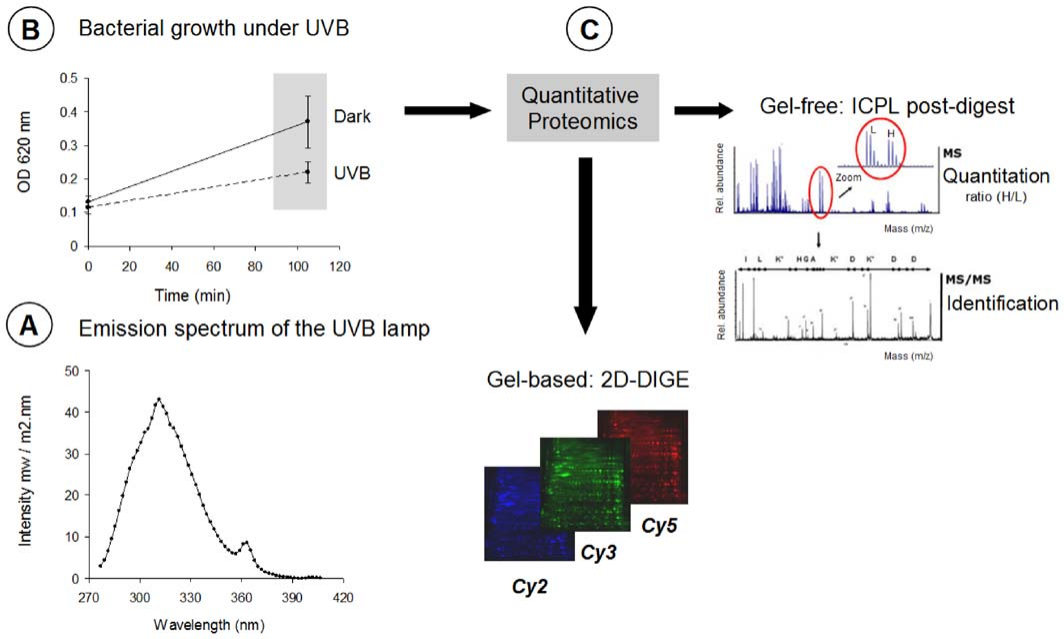
Summary of the experimental workflow. **A**. Emission spectrum of the UVB lamps used to irradiate the cultured cells of *P. angustum* **B**. Bacterial cells were collected at the beginning of log phase (OD = 0.1) and cultured either under UVB radiation or in the dark **C**. After 1.75 h of incubation, quantitative proteomics was performed by using either gel-free (Post-digest ICPL labeling) or gel-based (2D-DIGE) approaches. Quantitative data for ICPL were obtained from the MS spectrum (L: Light label: H: Heavy label).

After two precultures, *Photobacterium angustum* S14 was grown aerobically in 500 mL of Artificial Sea Water (ASW) supplemented with 3 mM D-glucose (ASW-G), vitamins and trace elements [15] in a rotary shaker (130 rpm) at 25°C. The growth was monitored by optical density (OD) measurements at 620 nm. At the beginning of the mid-log phase (OD = 0.1), cells were harvested and split into two parts for both the UVB irradiation and dark control conditions. For this purpose, 120 mL of culture was placed into a 250-mL quartz flask with a flat top covered with cellulose acetate (50% transmission at 280 nm) to remove residual UVC and exposed to UVB lamps (30 W/312 nm, Vilbert Lourmat, Fisher scientific); another 120 mL was maintained in darkness as a control. The UVB intensity used in this study corresponds to 1.05 W/m^2^. Irradiated cells and their respective dark controls were mixed with continuous magnetic stirring (100 rpm) for 1.75 h (corresponding to a UVB dose of 6.6 kJ/m^2^) at 25°C. At the end of this period, the OD was measured, and the cells were harvested for proteomics (Figure 1). The entire volume of the cultures from both conditions was centrifuged at 8 000 *g* for 15 min at 4°C. Based on methods previously used in another marine bacterium, the resulting pellet was washed twice in 1 mL of 0.2 M sucrose to remove excess salt [16] and freeze-dried at −80°C until use. Protein isolates were subsequently used for either the post-digest ICPL approach or 2D-DIGE method. For each proteomics approach, experiments were performed in quadruplicate for a total of eight independent experiments.

### Protein extraction and quantification

For the post-digest ICPL method, pelleted cells were resuspended in one pellet volume of 6 M guanidine chloride solution, and cells were broken by sonication on ice (5 cycles of 10 s, amplitude 60%, 1 pulse rate). The samples were subsequently centrifuged at 16 000 *g* at 4°C for 15 min. Protein samples were reduced with 5 mM Tris (2-carboxyethyl) phosphine at 60°C for 30 min and alkylated with 0.4 µM iodoacetamide at 25°C for 30 min. Proteins were recovered by acetone precipitation (2 h) with an acetone/protein ratio of 4/1. After a 15-min centrifugation at 16 000 *g* and an acetone evaporation, the resulting pellet was dissolved in 100 mM phosphate buffer (pH 8) containing 2 M urea. Protein isolation for the 2D-DIGE application was performed by resuspending the pelleted cells in 1 mL of 2DE extraction buffer (8 M urea, 2 M thiourea, 4% CHAPS). Sonication and sample clearing by centrifugation were performed as mentioned above.

The total protein concentration for both ICPL and 2D-DIGE was determined using the Bradford method according to the manufacturer's instructions, using bovine *γ*-globulin as a protein standard (Bio-Rad Protein Assay kit, Bio-Rad, Hertfordshire, UK).

### Post-digest ICPL labeling

For the gel-free approach, an overnight enzymatic digestion was conducted with sequencing grade modified trypsin (Promega) at an enzyme/substrate ratio of 1/25 at 37°C prior to the labeling. Tryptic peptides were then labeled according to the post-digest ICPL labeling procedure, which was recently described and optimized by Leroy and colleagues [14]. Briefly, after enzymatic digestion, 33 µg of tryptic peptides were submitted for labeling using 3 µL of ICPL-labeling reagent for 90 min at room temperature. This first labeling step was followed by the addition of 1.5 µL of supplemental reagent, and the reaction was allowed to proceed for 90 additional minutes. The protein samples obtained from dark-grown cells were labeled with 6-[^12^C]nicotinoyl-*N*-hydroxysuccinimide (light label), while the protein samples obtained from cells grown under UVB were labeled with 6-[^13^C]nicotinoyl-*N*-hydroxysuccinimide (heavy label). After destroying any excess reagents according the manufacturer's instructions, the two samples were combined, and the resulting labeled peptide mixture was basified with 2 µL of NaOH (2N) for 20 min to destroy possible esterification products; subsequently, 2 µL of HCl (2N) was added to neutralize the sample. The peptide solution was desalted using HyperSep SpinTip C18, and 40 µg of the digested proteins were then analyzed by 2D LC-MS/MS.

### 2D-DIGE (minimal dye labeling)

We performed 2D Fluorescence Difference Gel Electrophoresis (2D-DIGE) as a second quantitative proteomic approach with a gel-based methodology. DIGE experiments were conducted 7 times with 4 different biological replicates and three extra technical replicates run in parallel. One preparative gel was used for spot picking; thus, a total of seven analytical DIGE-gels and 1 preparative gel were performed. All the gels were run in only two batches of migration, and the same internal standard sample was used for all gels. Fifty-µg aliquots of protein from both the UVB and dark conditions were labeled with Cy3 or Cy5, respectively, and a pooled internal standard was systematically labeled with Cy2. Dye swapping with Cy3 and Cy5 between the two conditions enabled us to exclude any potential bias from the labeling reaction. To achieve optimal DIGE labeling and avoid spontaneous thiol oxidation, all experiments were performed with minimal light exposure. All the samples were incubated separately with the corresponding CyDye for 30 min on ice in the dark, and the reactions were subsequently quenched by the addition of excess lysine for 10 min. These labeled samples were then combined for 2D-DIGE analysis. Thus, 50 µg of Cy3- and Cy5-labeled samples were mixed pairwise and pooled together with the internal standard. For the first dimension, pooled samples containing 150 µg of labeled proteins were mixed with rehydration buffer [8 M urea, 2 M thiourea, 2% (w/v) CHAPS, 10 mM DTT, 1.2% pH 4–7 IPG buffer (v/v) and trace bromophenol blue] in a final volume of 350 µL and left overnight to rehydrate into 18-cm Immobiline Drystrips (pH 4–7).

Isoelectric focusing was performed at 20°C using IPGphorIII (GE Healthcare) with a current limit of 50 µA/gel for 1 h at 150 V followed by 1 h at 300 V. A gradient voltage was then applied for 1 h to reach 1000 V and subsequently for 3 h to reach a voltage of 8000 V. Finally, the focusing was continued at 8000 V until a total of 40 kVh had elapsed. Strips were then equilibrated at room temperature for two intervals of 12 min in an equilibration buffer [6 M Urea, 2% (w/v) SDS, 30% (v/v) glycerol and 50 mM Tris-HCl pH 8.6] supplied with 1% (w/v) DTT, and the strips were subsequently incubated in the same buffer with 4.6% (w/v) iodoacetamide replacing DTT. Equilibrated strips were briefly rinsed with running buffer [25 mM Tris, 192 mM glycine and 0.1% (w/v) SDS] and placed onto a 12.5% precast gel overlaid with agarose solution [0.5% low-melting agarose and trace bromophenol blue in running buffer]. Electrophoresis was performed at 20°C in the dark using a DALT six electrophoresis system (GE healthcare) at 4 W/gel for 30 min, followed by 18 W/gel for approximately 6 h until the bromophenol blue dye front reached the bottom of the gel. After the second dimension of electrophoresis, the gels were rinsed with Milli-Q water and immediately scanned on a Typhoon FLA 9000 imager (GE Healthcare) with a 100-µm spot resolution using the appropriate voltages to ensure no spot saturation. The photomultiplier tube setting was in the range of 450 to 600 V. The gels were subsequently stored at 4°C.

For spot picking, one preparative gel from the same pH range was run (1 mg of protein lysate each) at the same time as the three other analytical and technical replicate gels; thus, similar electrophoretic parameters for the first and second dimension runs were used for both analytical and preparative gels. The preparative gel, containing 1 mg of protein, was then stained overnight with Coomassie brilliant blue and fixed with 40% (v/v) ethanol and 10% (v/v) acetic acid.

Image Master Platinum (V.2.0) software was used to analyze the gel images. Gel images were processed with 10 000 expected spots and spots with volumes higher than 30 000. Seven fluorescent gels were run with 4 biological and 3 technical replicates; thus, a total of 21 2D-gel images were obtained and used to calculate average abundance. Protein spot expression levels that showed a statistically significant (p<0.01) increase or decrease in 6 of 8 (Cy3 or Cy5 labeled) gels were accepted as being differentially expressed. A 1.25-fold difference in abundance with a t-test p-value of less than 0.01 was considered significant. Spots identified as significantly up- or down-regulated on analytical gels were manually excised from the preparative gels for identification via mass spectrometry. Destaining, reduction, alkylation, and trypsin digestion of the proteins were conducted prior to peptide extraction.

### Liquid Chromatography Tandem Mass Spectrometry (LC-MS/MS)

For the gel-free 2D LC-MS/MS approach, dried protein samples were resuspended in 8 µL of the loading solvent and separated using the LC Ultimate 3000 system (Dionex). Briefly, six microliters containing 40 µg of protein were loaded onto a strong cation exchange (SCX) column (POROS S10, 10 cm, Dionex) and eluted sequentially using 5, 10, 25, 50, 75, 100, 120, 150, 200, 400, and 800 mM of sodium chloride (20 µL). The eluted fractions from each salt step were concentrated and desalted on a pre-column (C18, 15 cm, 75 µm i.d., Dionex) at a rate of 20 µL min^−1^. After a 15-min wash with the loading solvent, the pre-column was switched into line with an analytical C18 column containing reverse-phase packing material (Dionex). Peptides were eluted using a linear gradient of acetonitrile (ACN) at a rate of 0.2 µL min^−1^. The ACN gradient was 4–37% solvent B (80% ACN, 0.08% formic acid) for 100 min, 37–57% solvent B for 10 min and 57–90% solvent B for 10 min; solvent B was held at 90% for 10 min and then reset to 4%. The eluted peptides were analyzed online by mass spectrometry as described below.

Regarding the samples obtained from the gel-based 2D-DIGE approach, purified peptides from digested protein samples were identified using the LC Ultimate 3000 system (Dionex) coupled with an HCT ultra plus ion-trap mass spectrometer (Bruker Daltonics, Germany). Six microliters of each fraction were loaded onto a pre-column (C18 Trap, 5 mm×300 µm i.d., Dionex) using the Ultimate 3000 system with a flow rate of 20 µL min^−1^ of loading solvent (5% (v/v) ACN, 0.025% (v/v) TFA). After a 10-min desalting step, the pre-column was switched online with the analytical column (15 cm×75 µm i.d., PepMap C18, Dionex) and equilibrated in 96% solvent A (0.1% (v/v) formic acid in HPLC-grade water) and 4% solvent B (80% (v/v) ACN, 0.1% (v/v) formic acid in HPLC-grade water). Peptides were eluted from the pre-column to the analytical column with a gradient of 4 to 57% solvent B for 50 min and 57–90% solvent B for 10 min prior to entering the mass spectrometer. The flow rate was maintained at 0.2 µL min^−1^ with the Ultimate pump. The separated peptides were analyzed online using an ion-trap mass spectrometer (HCT ultra plus ion-trap mass spectrometer, Bruker Daltonics, Germany). Positive ions were generated by electrospray, and the instrument was operated in the information-dependent acquisition mode described as follows: MS scan range: 300–1500 *m*/*z*, maximum accumulation time: 200 ms, ICC target: 200 000. The 4 most intense ions in the MS scan were selected for MS/MS in dynamic exclusion mode: mode = ultrascan, absolute threshold = 75 000, relative threshold = 1%, excluded after spectrum count = 1, exclusion duration = 0.3 min, and averaged spectra = 5.

### Data processing generated from mass spectrometry

All MS/MS spectra were analyzed using Mascot (Matrix Science, London, UK; version 2.2), which was set up to search a local copy of the *P. angustum* database (retrieved from NCBI on December 19^th^, 2007; 4558 proteins), using fragment precursor and product ion tolerances of ±1.3 and an MS/MS tolerance of ±0.5 Da with the maximum number of missed trypsin cleavage sites set to 2. The oxidation of methionine, the iodoacetamide derivative of cysteine (Cys-carbamidomethyl) and for gel-free quantitative proteomics samples, the post-digest ICPL quantification chemistry of lysine and the N-terminus were specified in Mascot as variable modifications. Only identified proteins with protein scores above the Mowse calculated ion score, defined as the 95% confidence level, were considered. In this manner, all spectra that matched the databases with a Mowse score of <35 were automatically rejected. All matching spectra with Mowse scores greater than 35 (p<0.05) were manually inspected to ensure ion progressions of at least 4 consecutive y- or b-type ions, especially for the proteins identified with one single peptide. Any matches that did not meet these criteria were rejected. All proteins identified based on only one peptide were further substantiated by reporting both the Mascot ion score and an annotated tandem mass spectrum in the Supporting Information (Figure S1). The false discovery rate (FDR) was calculated at the peptide level for all experimental runs using the Decoy option in Mascot; this rate was estimated to be lower than 2% using the identity threshold as the scoring threshold system. Regarding the identification of the excised spots, we extracted all the identified proteins from each spot and provided the emPAI values, which take into account the number of sequenced peptides per protein and have been shown to be directly proportional to protein content [17]. Nevertheless, the spots in which we identified multiple proteins were considered to be “ambiguously quantified”. Indeed, the protein with the highest emPAI value is not necessarily the one exhibiting a change in quantity; thus, emPAI values were simply used as indicative numbers of protein abundance and did not allow us to discriminate between different proteins. The relative quantification of proteins was achieved during MS/MS by comparing the estimated abundance of reporter ion peaks that corresponded to UVB cultured cells (ICPL Heavy) with those of the dark cultured cells (ICPL Light). MS/MS spectra were searched against the *P. angustum* database for protein quantification using Mascot distiller v.2.3.2 (Matrix Science, London, UK), and the search settings were similar to those used in Mascot. The global mean and SD of the protein ratios were calculated for each pair of samples. Peak lists (.baf files) from the ESI-TRAP data were generated using Mascot Distiller (Version 3.2.1.0, Matrix Science). The parameters for the Mascot Distiller quantification procedure were as follows: correlation threshold: 0.8; integrated method: Simpsons; XIC threshold: 0.2; Max XIC width: 7; and quality: fraction. To obtain reliable quantitative data, a manual validation of all the quantified peptides was performed to filter out co-eluted peptides and/or noise. A quantified protein was kept only if at least two top ranking well-identified peptides were provided with a Mascot score of more than 35. Data were recorded for proteins that showed an average increase in abundance above 1.25 or below 0.8 in the UVB sample relative to the dark control; the data significantly different from the control were denoted with bold characters (Table 1) using the comparison test provided by the Mascot distiller. The cut-off for significance in fold-change was determined based on two technical replicates, and we used 295 commonly identified proteins to monitor technical variations. Of these proteins, 87% fell within 25% of the experimental variation (Figure S2). Therefore, we considered a fold-change of >1.25 or <0.8 to be a meaningful cut-off for the identification of true differences in protein level between UVB and dark conditions.

**Table 1:** List of the quantified proteins in four biological replicates using the post-digest ICPL labeling methodology.

Proteins name	Rep 1			Rep 2			Rep 3			Rep 4		
	Ratio	SD	# Pept	Ratio	SD	# Pept	Ratio	SD	# Pept	Ratio	SD	# Pept
VAS14_02738 hypothetical protein	0.58	0.00	1	0.57	0.00	1	0.51	1.25	2	0.71	0.00	1
VAS14_16916 phosphocarrier protein HPr	0.62	1.33	3	**0.67**	1.11	5	**0.54**	1.21	6	**0.44**	1.20	5
VAS14_04158 arginine ABC transporter	0.68	0.00	1	0.70	0.00	1	**0.70**	1.28	2	0.99	0.00	1
VAS14_22197 peptide ABC transporter	**0.72**	1.04	3	0.67	1.16	2	0.59	1.32	2	**0.75**	1.20	6
VAS14_20236 phosphoglycerate kinase	**0.72**	1.41	4	0.92	1.21	18	**0.73**	1.19	19	**0.66**	1.24	10
VAS14_17626 asparagine synthetase B	**0.73**	1.31	6	**0.72**	1.20	9	0.80	1.16	16	1.01	1.32	12
VAS14_07409 carbamoyl-phosphate synthase small subunit	0.74	1.55	4	0.81	1.37	5	0.85	1.20	6	0.79	1.16	3
VAS14_10219 phosphoenolpyruvate synthase	**0.77**	1.25	9	**0.81**	1.31	20	**0.84**	1.24	26	**0.89**	1.24	21
VAS14_02983 putative Cold shock-like protein	**0.77**	1.17	5	**0.69**	1.20	5	**0.64**	1.11	5	**0.56**	1.13	6
VAS14_17766 succinyl-CoA synthetase alpha subunit	**0.78**	1.27	4	**0.83**	1.20	9	**0.88**	1.16	16	**0.51**	1.60	8
VAS14_07734 putative glutamate synthase, large subunit	**0.78**	1.21	10	**0.83**	1.42	16	0.85	1.24	22	**0.84**	1.36	18
VAS14_21427 phosphoenolpyruvate carboxylase	**0.79**	1.28	15	**0.87**	1.22	18	**0.75**	1.33	25	0.91	1.52	22
VAS14_14614 putative Cold shock-like protein	**0.79**	1.13	4	0.75	1.20	4	**0.81**	1.10	4	**0.59**	1.06	3
VAS14_08175 3-isopropylmalate dehydrogenase	**0.80**	1.10	5	**0.77**	1.27	6	0.82	1.45	8	0.81	1.26	4
VAS14_07404 carbamoyl-phosphate synthase large subunit	0.80	1.08	12	0.86	1.39	18	**0.87**	1.16	13	**0.88**	1.23	15
VAS14_08340 inorganic pyrophosphatase	0.84	1.30	6	**0.80**	1.22	6	**0.78**	1.14	7	0.86	1.26	8
VAS14_21527 triosephosphate isomerase	0.84	1.13	4	**0.71**	1.09	7	**0.84**	1.15	7	**0.71**	1.32	6
VAS14_10584 3-deoxy-7-phosphoheptulonate synthase	0.95	1.09	10	0.82	1.26	11	**0.80**	1.28	14	**0.80**	1.29	12
VAS14_05653 putative superoxide dismutase	0.98	0.00	1	0.88	1.23	3	**0.78**	1.14	5	**0.76**	1.18	5
VAS14_18824 putative membrane protease subunits	1.21	1.54	6	**1.21**	1.03	3	1.28	1.48	6	**1.43**	1.16	6
VAS14_07124 molecular chaperone DnaK	**1.24**	1.23	25	**1.14**	1.24	29	1.06	1.27	27	**0.89**	1.28	25
VAS14_10439 putative ATP-dependent RNA helicase	**1.26**	1.05	3	**1.40**	1.32	8	1.70	2.12	9	**1.30**	1.23	6
VAS14_12874 hypothetical outer membrane protein OmpA	**1.28**	1.23	7	**1.33**	1.29	13	0.96	1.19	13	1.07	1.17	8
VAS14_06218 trigger factor	**1.31**	1.23	17	**1.19**	1.24	27	**1.18**	1.22	30	**1.15**	1.21	29
VAS14_09799 glyoxalase family protein	1.33	1.14	3	1.15	1.04	2	1.21	1.23	2	1.79	1.56	2
VAS14_05963 hypothetical outer membrane protein OmpA	1.36	1.31	3	1.37	1.42	4	1.00	1.10	6	1.11	1.22	5
VAS14_07334 translation initiation factor IF-2	**1.45**	1.38	17	**1.27**	1.31	15	1.35	1.56	26	1.23	1.46	13
VAS14_18834 RNA-binding protein Hfq	1.46	1.69	2	3.08	0.00	1	1.54	0.00	1	3.45	0.00	1
VAS14_00891 putative glutaredoxin 1	**1.47**	1.04	2	1.32	1.08	2	0.94	0.00	1	1.41	1.31	2
VAS14_06208 ATP-dependent protease ATP-binding subunit	1.49	1.14	2	1.26	1.31	5	**1.35**	1.17	7	1.80	0.00	1
VAS14_21207 putative FKBP-type peptidyl-prolyl cis-trans isomerase 1	1.50	1.66	2	**1.62**	1.23	7	1.10	1.26	7	1.18	1.21	5
VAS14_07384 ompL_phopr porin-like protein L precursor	**1.56**	1.11	3	0.96	1.22	3	**1.35**	1.18	5	1.76	1.27	3
VAS14_16369 putative GTP-binding protein	1.64	1.47	3	1.32	1.17	3	**1.53**	1.17	2	1.45	1.18	2
VAS14_06783 putative antioxidant, AhpC/Tsa family protein	**1.65**	1.37	17	**1.67**	1.24	20	**1.41**	1.15	17	**1.58**	1.16	21
VAS14_16756 Na(+)-translocating NADH-quinone reductase subunit F	1.65	1.29	3	**1.37**	1.28	6	1.18	1.41	12	**1.76**	1.11	6
VAS14_14319 hypothetical protein	1.81	0.00	1	1.70	1.34	2	**1.79**	1.06	2	**1.57**	1.17	3
VAS14_06728 putative inositol monophosphate family protein	1.91	1.23	2	1.54	1.79	5	1.86	1.12	8	1.61	0.00	1
VAS14_11709 putative lipoprotein	2.01	1.32	3	**3.31**	1.06	3	**2.01**	1.25	4	2.09	2.33	3
VAS14_07364 putative cell division protein FtsH	**2.32**	1.26	4	**2.28**	1.23	5	1.07	1.22	10	**1.66**	1.19	7
VAS14_20496 recombinase A	**2.79**	1.15	3	**2.91**	1.29	8	2.62	1.09	17	3.35	0.00	1

Proteins significantly different from the control within a given biological replicate are indicated with bold characters. **Ratio**: UVB/Dark, **SD**: standard deviation, **#Pept**: number of peptides used for protein quantification.

### Fluorescent western blot

Immunoblotting was used to confirm the changes in abundance of the well-conserved protein RecA detected by both 2D-DIGE and ICPL. Pelleted cells were lysed as described previously, and the supernatant was used for fluorescent western blotting analysis with ECL Plus (GE Healthcare). The protein samples were separated on a NuPAGE 4–12% Bis-Tris gel (Invitrogen), blotted onto a nitrocellulose membrane (Hybond-ECL; GE Healthcare) and probed with a mouse monoclonal antibody against RecA (1∶1000 dilution; Clone ARM321, Clinisciences, France) as the primary antibody. The amino acid sequence of RecA recognized by the primary antibody is presented in Figure S3. A sheep secondary antibody anti-mouse HRP-conjugated F(AB)_2_ fragment (GE Healthcare) was added in a 1∶5000 dilution. Membranes were air-dried, scanned using a Typhoon FLA 9000 (GE Healthcare) and quantified using ImageQuant analysis software (GE Healthcare).

## Results and Discussion

### Comparison of gel free and gel-based methods

**Figure 2:**
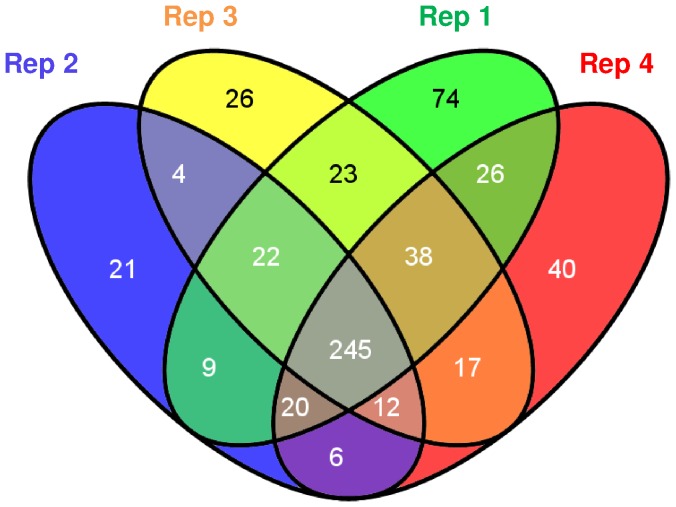
Venn diagram showing the non-redundant proteins quantified in four biological replicates.

**Figure 3:**
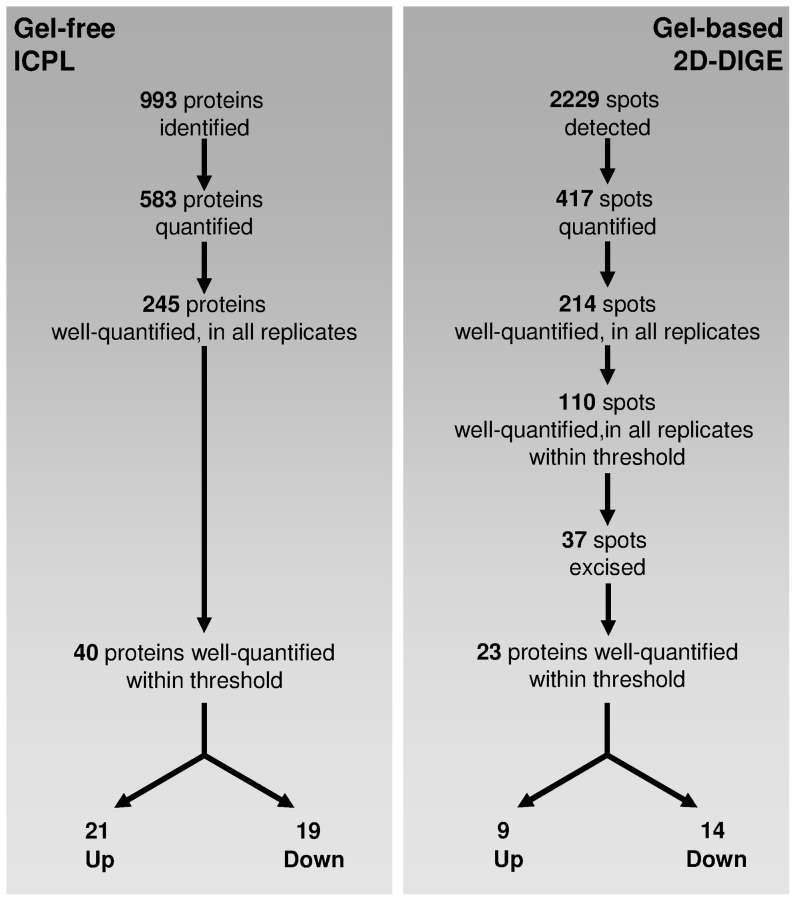
Comparison of the different steps of protein identification and quantification between the two experimental workflows.

After a 1.75-h incubation, a 43% increase in OD_(620 nm)_ was observed for the UVB irradiated cells, which was lower than that observed in the dark control cultures (Figure 1). A total of 993 proteins were identified by liquid chromatography-tandem mass spectrometry (LC-MS/MS), corresponding to 22% of the theoretical proteome of *P. angustum* (4558 proteins). Four different biological replicates were conducted for each of the two quantitative proteomics methods; thus, a total of 8 independent experiments were successfully performed. Regarding the four biological replicates conducted for the ICPL approach, an average of 400 proteins were quantified in each MS run. The distribution of the fold changes for all the ICPL-labeled proteins in each biological replicate ranged from 0.15 to 3.34. In total, 422 proteins were common in at least two different replicates, and 245 were detected in all four replicates, representing an overall average of 25% of the expressed proteome (Figure 2). In total, 583 ICPL-labeled non-redundant proteins had significant differential abundance compared with the dark control. The common set of proteins affected by UVB treatment (245 proteins) was manually inspected to ensure that both identification and relative quantification were correctly performed. After applying the commonly used cut-off thresholds of 1.25 and 0.8, only proteins exhibiting similar regulation profiles in all the replicates were retained as relevant proteins affected by UVB treatment. In summary, proteins that did not exhibit consistent up- or down-regulation between experiments were not considered good markers of UVB stress and were consequently removed from our final list of proteins of interest. By using these stringent criteria, a set of 40 proteins of interest were obtained; among these, 19 and 21 proteins were found to be down- and up-regulated, respectively (Table 1, Figure 3).

**Figure 4:**
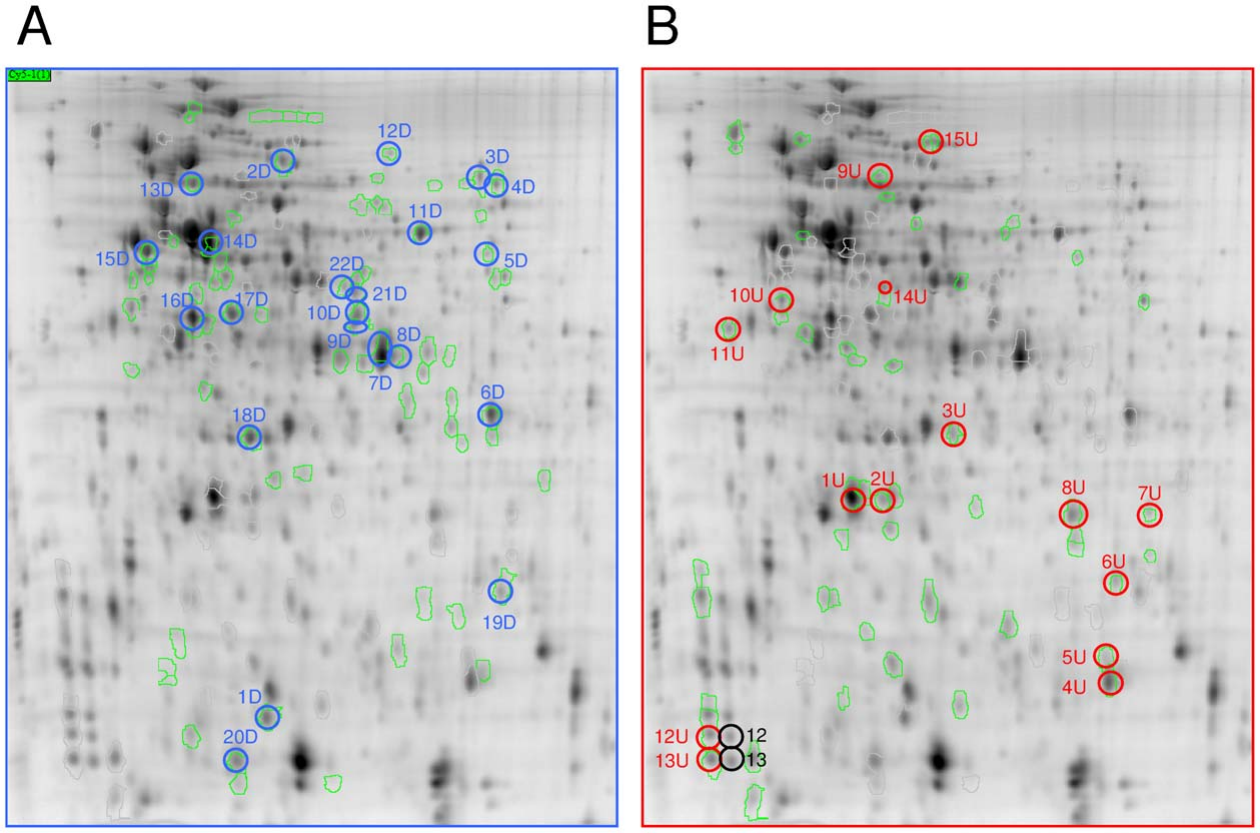
A representative standard 2D gel showing protein spots that were down-regulated (A) and up-regulated (B). Proteins of interest are circled in green, and excised spots are circled in blue or red according their relative spot volumes (D = down-regulated by UVB; U = up-regulated by UVB). Green circles correspond to the up- or down-regulated proteins not excised for MS analysis.

In this study, 2D-DIGE was also used to investigate the changes in protein expression associated with UVB treatment. The images obtained for each replicate are presented in Figure S4 and demonstrated a high level of reproducibility from one experiment to another. A total of 2229 spots were detected and matched among the seven gels. Of these, 417 spots were quantified and subjected to manual inspection. After validation, 214 spots were found to be well resolved in all replicates, and thus considered as well-quantified. Spots of interest were assigned by filtering out spot values using the average volume ratios of 1.25-fold changes in expression and selecting those that had a one-way ANOVA p-value of 0.01 or less. A total of 130 spots satisfied these requirements and were highlighted as spots of interest and circled in green in Figure 4. Among the 130 spots of interest, 76 and 54 spots presented an abundance ratio corresponding to either down- or up-regulation, respectively (green circles, Figure 4A and 4B, respectively). Unfortunately, all the spots of interest could not be excised due to overlapping spots, weak signal intensity, positional differences between spots, and mismatched protein spots. A total of 37 spots of interest were finally excised, trypsin digested and submitted to MS/MS for identification (Figure 3). The 37 spots yielded 47 MS/MS identifications due to co-migration issues. Thus, after manual inspection, 23 spots were properly identified, providing only one protein with a good score. Nine proteins were found to be up-regulated, and 14 were down-regulated (Table 2, Figure 3). The co-migration of proteins is the main drawback of 2D gel analysis. For 15 of the spots, it was not possible to ascertain which of the proteins within the mixture were altered in abundance; thus, an accurate quantification was not obtained for the following spots: 2D, 5D, 8D, 9D, 12D, 13D, 21D, 22D, 1U, 2U, 3U, 7U, 9U, 11U, and 14U (Table 2). The proteins identified as being significantly regulated using ICPL have been cross-validated using 2D-DIGE, and we identified a total of 23 proteins that were accurately quantified (one protein per spot) in the 2D-DIGE dataset (Figure 3). To summarize, the total number of proteins differentially regulated using both approaches was 55 [40+23-8]. Furthermore, cross-validation using both methods provided some clues regarding co-migratory proteins. Taking the ICPL data into account, we could hypothesize that for the 1U and 14U spots, the abundance of both the AhpC antioxidant protein (VAS14_06783) and RecA protein (VAS14_20496), respectively, were modulated under UVB radiation. Interestingly, the 2D-DIGE allowed us to distinguish a series of protein isoforms of the lipoprotein (VAS14_1709) behind spots 12, 12U and 13, 13U (Figure 4, Table 2). Unfortunately, we were not able to characterize the post-translational modification of this protein. The majority of protein isoforms are the result of post-translational modifications (PTMs) generated by metabolic processes to achieve specific functions; alternatively, they might be generated by various stresses. The study of isoforms could be very informative, and they might represent a new class of biomarkers for a given stress because one particular stress could be associated with only one specific isoform of the protein. Interestingly, it was previously reported that a same PTM (β-Methylthio-aspartic acid) within a well-conserved ribosomal protein, which has an almost identical amino acid sequence in *E. coli* and *S. oneidensis*, plays a key role in stabilizing ribosomal structure [18]; [19]. However, the current understanding of PTMs in bacteria is very limited, both in terms of the types of modifications and the frequency of their occurrence, because these modifications are more often encountered in eukaryotic cells than prokaryotes.

**Table 2:** Identification of the two-dimensionally separated protein spots of interest with the average fold change and associated p-values obtained from seven replicates.

Spot	Protein name	Ratio	p value (ANOVA)	# Peptides	Mass	Score	empAI
4D	VAS14_05498 putative oligopeptide ABC transporter,periplasmic oligopeptide-binding protein	0.34	3.70E-07	24	61324	1118	2.00
20D	VAS14_09609 hypothetical protein	0.53	1.51E-04	7	13023	290	3.07
7D	VAS14_06013 glyceraldehyde-3-phosphate dehydrogenase	0.53	7.12E-11	19	35441	2084	3.58
8D	VAS14_06013 glyceraldehyde-3-phosphate dehydrogenase	0.53	7.12E-11	41	35441	2084	3.58
	VAS14_14989 transaldolase			5	35103	274	0.31
6D	VAS14_04158 arginine ABC transporter, periplasmic arginine-binding protein	0.56	2.33E-06	15	26884	729	2.62
13D	VAS14_08125 pyruvate kinase	0.57	1.94E-09	16	50710	946	1.13
	VAS14_05498 putative oligopeptide ABC transporter,periplasmic oligopeptide-binding protein			16	61324	534	0.97
15D	VAS14_00448 phosphoribosylaminoimidazole synthetase	0.57	3.27E-07	12	37159	521	0.82
14D	VAS14_20441 phosphopyruvate hydratase	0.60	4.79E-07	79	45977	4993	7.59
9D	VAS14_10584 3-deoxy-7-phosphoheptulonate synthase	0.60	1.07E-06	17	38616	1119	2.16
	VAS14_20241 fructose-bisphosphate aldolase			4	38792	165	0.28
16D	VAS14_20441 phosphopyruvate hydratase	0.66	6.49E-04	2	45977	50	0.07
19D	VAS14_00681 bacterioferritin comigratory protein	0.67	3.40E-07	11	17417	163	1.43
2D	VAS14_19086 bifunctional phosphoribosylaminoimidazolecarboxamide formyltransferase	0.67	1.37E-08	11	57424	674	0.88
	VAS14_18544 30S ribosomal protein S1			8	60777	544	0.52
	VAS14_17821 arginyl-tRNA synthetase			7	64732	474	0.48
3D	VAS14_22197 peptide ABC transporter, periplasmic peptide-binding protein	0.70	2.77E-06	10	57916	480	0.39
11D	VAS14_16581 serine hydroxymethyltransferase	0.70	9.94E-09	24	45287	609	1.68
12D	VAS14_16581 serine hydroxymethyltransferase	0.71	7.30E-08	5	45287	422	0.33
	VAS14_18544 30S ribosomal protein S1			4	60777	246	0.17
	VAS14_06013 glyceraldehyde-3-phosphate dehydrogenase			2	35441	146	0.2
	VAS14_22994 glutamine synthetase			3	51536	121	0.2
18D	VAS14_21527 triosephosphate isomerase	0.71	2.55E-10	8	26968	374	0.59
1D	VAS14_14099 16 kDa heat shock protein A	0.75	2.95E-06	4	16561	325	1.11
10D	VAS14_20756 putative 2,3,4,5-tetrahydropyridine-2-carboxylate N-succinyltransferase	0.75	1.38E-08	15	36213	369	1.4
21D	VAS14_05668 aspartate-semialdehyde dehydrogenase	0.75	9.39E-06	13	40791	513	1.18
	VAS14_08310 malate dehydrogenase			2	32486	212	0.21
22D	VAS14_12994 glycine cleavage system protein T2	0.76	2.85E-04	6	40822	311	0.6
	VAS14_09604 acetyl-CoA acetyltransferase			6	41768	316	0.46
5D	VAS14_20221 S-adenosylmethionine synthetase	0.79	3.77E-05	2	42360	124	0.16
	VAS14_05498 putative oligopeptide ABC transporter,periplasmic oligopeptide-binding protein			4	61324	180	0.10
17D	VAS14_08310 malate dehydrogenase	0.79	4.67E-06	53	32486	2873	7.49
3U	VAS14_19241 30S ribosomal protein S3	1.26	5.38E-06	2	25439	86	0.28
	VAS14_02743 hypothetical protein			2	30191	67	0.11
	VAS14_20076 3-methyl-2-oxobutanoate hydroxymethyltransferase			2	28835	58	0.12
4U	VAS14_19236 50S ribosomal protein L22	1.26	2.73E-05	3	12172	46	0.28
11U	VAS14_16921 putative cysteine synthase A	1.33	1.55E-06	7	34364	348	0.74
	VAS14_09694 hypothetical ABC transporter, substrate binding protein			11	36644	226	0.68
12U	VAS14_11709 putative lipoprotein	1.33	1.48E-05	11	9172	204	1.62
13U	VAS14_11709 putative lipoprotein	1.33	6.75E-06	4	9172	234	1.62
9U	VAS14_18941 chaperonin GroEL	1.38	5.27E-07	5	57359	234	0.25
	VAS14_22517 bifunctional N-acetylglucosamine-1-phosphate uridyltransferase			5	48395	102	0.12
8U	VAS14_20971 ribosome releasing factor	1.40	2.35E-08	8	20820	431	1.45
15U	VAS14_19006 acetyl-coenzyme A synthetase	1.41	5.73E-05	8	72060	556	0.2
5U	VAS14_19236 50S ribosomal protein L22	1.50	9.95E-04	2	12172	118	0.28
6U	VAS14_08110 hypothetical protein	1.60	4.07E-07	2	16134	137	0.47
7U	VAS14_19181 transcription antitermination protein NusG	1.61	1.27E-06	4	16895	218	1.08
	VAS14_20971 ribosome releasing factor			2	20820	65	0.16
2U	VAS14_00453 uracil phosphoribosyltransferase	1.79	5.24E-04	2	22712	77	0.32
	VAS14_06783 putative antioxidant, AhpC/Tsa family protein			3	22576	48	0.32
10U	VAS14_07384 ompL_phopr porin-like protein L precursor	1.86	5.08E-06	25	37973	1438	4.79
1U	VAS14_19811 stringent starvation protein A	2.66	2.62E-09	3	24257	133	0.47
	VAS14_06783 putative antioxidant, AhpC/Tsa family protein			7	22576	118	0.32
14U	VAS14_12129 NADH:flavin oxidoreductase	3.29	1.04E-06	11	40302	434	0.61
	VAS14_20496 recombinase A			4	37902	78	0.12

The ratio was determined by comparing the peak intensities in LC-MS runs of UVB *versus* dark conditions (UVB/Dark). The number of peptides assigned a Mascot score above 35, as well as the protein mass, score and empAI value, are also listed for each identified protein. (D: down-regulated; U: up-regulated).

When comparing both approaches, we demonstrated that the post-digest ICPL approach enabled the simultaneous identification and quantification of a high number of proteins, which is suitable for a large scale screening upon a given stress. Interestingly, we observed a series of isoforms using the DIGE method, and only one specific protein state was found to be modified in its relative abundance ratio; this result was not accessible using ICPL. Although the DIGE method was more effective for the identification of protein isoforms and the statistics for multiplexed quantification are easier to implement, this technique suffers from a few major drawbacks. One of these is the co-migration of proteins that may interfere with accurate protein identification; another drawback is the difficulty in the analysis of basic, hydrophobic and large proteins. These two approaches appeared to be complementary, and each method was useful for discovering a unique set of novel differentially regulated proteins. The combination of both approaches offered a great potential for biomarker discovery; proteins that are significantly up- or down-regulated and able to be detected by both methods would constitute good biomarkers. Thus, 8 proteins identified with both methods and most importantly, regulated in the same manner (4 down-regulated and 4 up-regulated) constitute the UVB stress biomarkers in *P. angustum* (Table 3).

**Table 3:** UVB protein biomarkers of *P. angustum* quantified by both ICPL and 2D-DIGE.

Protein name	Ratio (UVB/Dark)	
	ICPL	2D DIGE
VAS14_04158 arginine ABC transporter	0.76	0.56
VAS14_10584 3-deoxy-7-phosphoheptulonate synthase	0.84	0.60
VAS14_21527 triosephosphate isomerase	0.78	0.72
VAS14_22197 peptide ABC transporter	0.68	0.70
VAS14_06783 putative antioxidant, AhpC/Tsa family protein	1.57	2.66 *1.79*
VAS14_07384 ompL_phopr porin-like protein L precursor	1.37	1.86
VAS14_20496 recombinase A	2.90	3.29
VAS14_11709 putative lipoprotein	2.30	1.33

* : 2 values are indicated for VAS14_06783 with 2D-DIGE because it was identified under 2 spots (1U, 2U).

### Differentially regulated proteins

Proteins that exhibited changes in abundance belonged to many functional categories and comprised proteins predicted to be localized to the cytoplasm as well as the cell membrane.

### DNA repair

**Figure 5:**
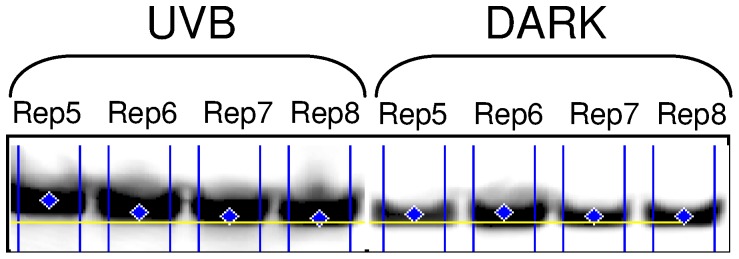
Detection of the RecA protein (≈40 kDa) by quantitative fluorescent western blot analysis of both UVB-treated cells and dark control cultures. Four different biological replicates are shown for each condition.

The RecA protein (RecA, VAS14_20496) is the key player involved in homologous recombination repair, which is one of the major DNA damage removal process in bacteria. RecA was found to be significantly up-regulated (3-fold, Table 3; 2-fold, Figure 5) under UVB treatment compared with the dark control. This result was obtained using three different quantitative proteomics approaches: ICPL, 2D-DIGE and fluorescent western blotting analysis (Table 3, Figure 5). Western blotting thus provided further confirmation of the robust methodologies used in this study (Figure 5). Taken together, these results indicate that RecA is a key UVB-biomarker in *P. angustum*.

**Figure 6:**
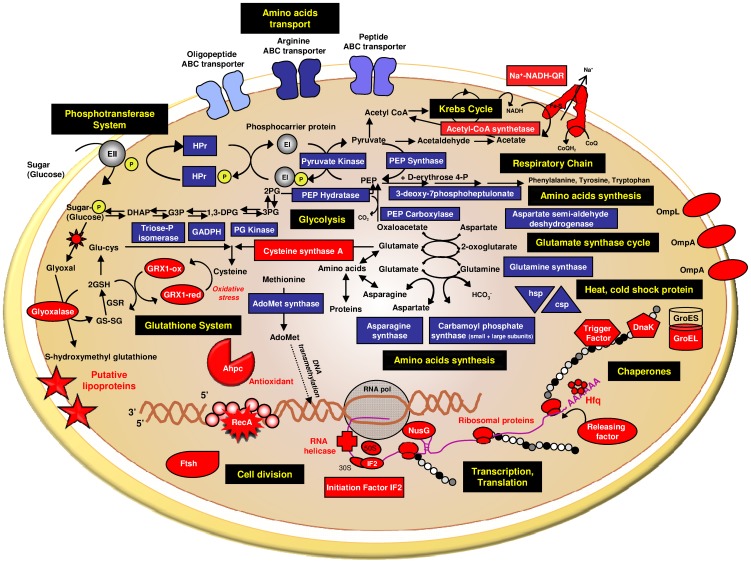
Diagram depicting the cellular pathways influenced by UVB radiation in *P. angustum*. The up- and down-regulated proteins are represented in red and blue, respectively.

The RecA protein plays multiple roles in several biochemical pathways, including recombinational processes, SOS induction, and mutagenic lesion bypass [20]. Briefly, the RecA protein binds strongly to ssDNA, thus coating the DNA to form a protein-DNA filament (Figure 6). Due to the presence of several DNA binding sites, the RecA protein can hold both single and double strands together simultaneously, thus promoting the exchange of homologous strands. It was previously demonstrated that radioresistance in *Deinococcus radiodurans* was related to its incredibly efficient system for the repair of double-stranded DNA breaks, which involved RecA-dependent recombinational repair [21]. Thus, in *P. angustum*, the key protein RecA could also play a critical role in UVB tolerance. However, cyclobutane pyrimidine dimers (CPDs) and pyrimidine (6-4) pyrimidone photoproducts (6-4PPs) are the predominant lesions caused by UVB radiation [22]. Booth et al. (2001) demonstrated a good correlation between loss of cell viability, accumulation of CPDs and RecA expression in *Vibrio natriegens* [23]. Interestingly, a previous study showed that CPDs were a major source of UV-induced DNA breaks [24]. Indeed, during DNA replication, non-excised and non-split CPDs trigger the formation of single-stranded gaps in the opposite DNA strand. Thus, the most prominent pathway induced by CPD lesions is that associated with DNA double-strand break (DSB) signaling and repair. Interestingly, in our experiment, RecA was expressed under dark conditions; this was demonstrated with ICPL, 2D-DIGE and more strikingly, the western blot analysis (Figure 5). RecA could thus respond to the continuous presence of DNA damage in the genome of *P. angustum*. In contrast, in *D. radiodurans*, the low basal level of RecA is not detectable by immunoprobe because of the critical threshold of expression [25]. However, it has been demonstrated that high concentrations of the RecA protein can partially suppress the sensitizing effect of the *ddrA* mutation in *D. radiodurans* exposed to gamma-irradiation, either by protecting single-stranded tails from degradation or inducing the recombinational repair pathway more rapidly [25]. Furthermore, in addition to recombinational repair, RecA is also involved in the regulation of the SOS response, which in turn regulates other repair pathways, including excision repair and translesion DNA synthesis repair. To investigate the role of RecA in the UVB resistance of *P. angustum*, it would be valuable to mutate this gene.

### Oxidative stress

A number of proteins that were more abundant in the *P. angustum* grown under UVB can be linked to the prevention of or the response to oxidative stress. During UVB exposure, reactive oxygen species (ROS) may be formed that subsequently damage DNA, proteins and membrane lipids. It is therefore not surprising that *P. angustum* contains genes to protect itself against oxidative stress. Proteins more abundant under UVB have been found to protect against oxidative DNA damage; these proteins include glutaredoxin, glyoxalase, the antioxidant AhpC and RecA (Figure 6). Superoxide Dismutase (SOD) (VAS14_05653, Table 1), which was identified with the post-digest ICPL method and reliably quantified in all four biological replicates, surprisingly exhibits a slightly smaller abundance under UVB treatment compared with the dark control.

In this study, one glutaredoxin (VAS14_00891, Table 1) was more abundant under UVB treatment. Disulfide bonds are required for proper protein folding and enhance protein stability and/or activity. The formation and reduction of disulfide bonds is catalyzed by specialized thiol-disulfide exchanging enzymes that contain an active site, and the major enzymes responsible for this process are thioredoxins and glutaredoxins. Glutaredoxins are small redox enzymes that are oxidized by substrates and use glutathione as a cofactor. They are subsequently regenerated by glutathione, and the oxidized glutathione is then reduced by glutathione reductase. Together, these components compose the glutathione system (Figure 6) [26], [27]. Glutaredoxins are also involved in alternative pathways, such as the formation of deoxyribonucleotides for DNA synthesis (by reducing the essential enzyme ribonucleotide reductase), generation of reduced sulfur (via 3′-phosphoadenylylsulfate reductase), signal transduction, and defense against oxidative stress [27]. Glyoxalase (VAS14_09799, Table 1) was found to be up-regulated under UVB radiation. Glyoxal may be produced through various pathways, including glucose oxidation, degradation of glycated proteins or lipid peroxidation, and may impair the function of membranes, proteins and DNA through alkylation, mutation and crosslinking reactions [28], [29]. If the glyoxal can be removed by the glyoxalase system, this minimizes the oxidative stress (Figure 6) [30].

The antioxidant Alkyl hydroperoxide reductase (AhpC)/Thiol specific antioxidant (TSA) (VAS14_06783, Tables 1, 2, 3) constitutes one biomarker of the UVB response in *P. angustum*, exhibiting 1.5-fold changes using the ICPL approach compared with 2.66-fold changes using 2D-DIGE (note that due to co-migration issues, we cannot assuredly ascertain the function behind this spot) (Tables 1, 2, 3). The AhpC protein is responsible for reducing organic hyperoxides and constitutes an important enzymatic defense against sulfur-containing radicals [31] and is of particular interest due to its putative functional role. The antioxidant AhpC protein will be the focus of future experiments to more accurately investigate its involvement in the UVB stress response of *P. angustum*.

### Transport proteins

Three different ATP-binding Cassette (ABC) transporters involved in amino acid or peptide uptake exhibited a decreased level of abundance upon exposure to UVB (VAS14_04158; VAS14_22197; VAS14_05498, Tables 1, 2). Both arginine and peptide ABC transporters (VAS14_04158 and VAS14_22197) were characterized as down-regulated UVB biomarkers (Table 3). Active transport across membranes is a vital function in cells and often performed by the ABC transporter system, which is involved in the transport of multiple substrates across membranes, including metabolites, proteins and drugs [32]. Similar to what was observed in the transcriptome of *Shewanella oneidensis* exposed to ionizing radiation, amino acid transport was down-regulated in *P. angustum* [33]. The repression of amino acid and peptide transport could decrease the energy requirement in UVB-treated cells and thus limit the generation of additional ROS [33].

Three other transport proteins classified as outer membrane proteins (OmpL, VAS14_07384; OmpA VAS14_12874 and VAS14_05963; Tables 1, 2, 3) were up-regulated under UVB radiation. The OmpL protein was found to be an up-regulated UVB biomarker (Table 3). Gram-negative bacteria are surrounded by a characteristic additional membrane layer referred to as the outer membrane, and its main role is usually to serve as a permeability barrier to prevent the entry of toxic compounds as well as to allow the influx of nutrients [34]. The outer membrane proteins act as porins and allow for the efficient diffusion of small and hydrophilic solutes. Although the specific substrate of the OmpL channel remains unknown [35], the OmpA channel is the most well-studied major outer membrane protein of *Escherichia coli* and functions as a bacteriophage receptor [36]. Interestingly, a previous study demonstrated that an OmpA deletion mutant of *E. coli* was significantly more sensitive than the wild-type strain to multiple stresses, such as sodium dodecyl sulfate (SDS), cholate, acidity and high osmolarity [37]. These results indicated that OmpA contributes to the structural integrity of the outer membrane. Thus, the increased abundance of three outer membrane proteins in UVB-irradiated *P. angustum* suggests that the maintenance of a stable outer membrane during exposure to damaging UVB radiation is important. These proteins are thus likely to play a vital structural role in *P. angustum*.

### Metabolism

Several enzymes that participate in general metabolic pathways, such as the phosphotransferase system, glycolysis, the Krebs cycle and amino acid synthesis, were found to be affected by UVB radiation. Three proteins involved in the phosphotransferase system were found to be down-regulated under UVB radiation: the Hpr protein (VAS14_16916), pyruvate kinase (VAS14_08125) and PEP synthase (VAS14_10219) (Tables 1, 2). This major ATP-dependent carbohydrate transport system catalyzes the phosphorylation of incoming sugar substrates concomitantly with their translocation across the cell membrane. The phosphoryl group from phosphoenolpyruvate (PEP) is transferred to the phosphoryl carrier protein HPr by enzyme I. Phospho-HPr then transfers the phosphoryl group to the permease (enzyme II) (Figure 6) [38], [39]. Three enzymes involved in the glycolysis pathway were found to be down-regulated: triosephosphate isomerase (VAS14_21527; Table 3), glyceraldehyde-3-phosphate dehydrogenase (GADPH, VAS14_06013, Table 2), and phosphoglycerate kinase (PG kinase, VAS14_20236, Table 1) (Figure 6), as well as the malate dehydrogenase involved in the Krebs cycle (VAS14_08310, Table 2). Overall, a UVB-stressed cell would preferentially use energy for UV resistance mechanisms (protection/repair) rather than general metabolism, which is energetically costly. A large number of enzymes involved in amino acid synthesis, including aspartate-semialdehyde dehydrogenase (VAS14_05668, Table 2), asparagine synthase (VAS14_17626, Table 1), the small and large subunits of carbamoyl-phosphate synthase (VAS14_07409 and VAS14_07404, Tables 1, 2), glutamate synthase (VAS14_07734, Table 1), 3-deoxy-7-phosphoheptulonate synthase (VAS14_10584, Tables 1, 2), serine hydroxymethyl transferase (VAS14_16581, Table 2), and S-adenosylmethionine synthetase (VAS14_20221, Table 2) showed a decreased abundance upon UVB irradiation. The carbon backbones of amino acids come from other major pathways, such as the glycolysis pathway or Krebs cycle. Consequently, enzymes involved in amino acid synthesis were also found to be down-regulated under UVB treatment.

In contrast, the NADH-quinone reductase (VAS14_16756, Table 1), which functions as a molecular energy converter by catalyzing the electron transfer from NADH to ubiquinone, was found to be up-regulated under UVB by approximately 1.5-fold. The significant amount of energy released from the oxidation of NADH allows for the pumping of sodium ions instead of protons [40], [41]. The respiratory chain of marine bacteria requires Na^+^ for maximum activity. Subsequently, the electrochemical gradient generated by the sodium supplies energy that can be used for a wide range of diverse functions, such as chemical (ATP) synthesis, osmotic activities, toxin efflux and motility [40], [41]. By increasing the pumping of Na^+^, *P. angustum* would be committing more energy to UV resistance. Furthermore, it was previously reported in *Helicobacter pylori* that NADH-quinone reductase enhances oxidative stress resistance [42].

### Transcription/Translation

The RNA helicase (VAS14_10439, Table 1), which is involved in nearly all aspects of RNA metabolism, was found to be up-regulated upon UVB stress. RNA helicases catalyze the separation of double-stranded DNA and RNA and function as transcriptional activators [43]. The RNA chaperone Hfq (VAS14_18834, Table 1) presented a higher abundance under UVB treatment. This protein is known to bind small regulatory non-coding RNA (sRNAs) and facilitate post-transcriptional gene regulation by helping these sRNA identify their mRNA targets during stress responses [44], [45], [46]. Hfq was also found to bind tRNA and the poly(A) tail of mRNA [47], [48]. The sRNA-mRNA duplex inhibits mRNA function by increasing mRNA degradation, decreasing mRNA translation, or both [49]. Interestingly, it has been previously reported that an *hfq* mutant of *E. coli* exhibited pleiotropic phenotypes, including an increased sensitivity to UV radiation [50]. It has also been reported that the Hfq protein modulates the sigmaE-mediated envelope stress response and sigma32-mediated cytoplasmic stress response in *E. coli* [51]. Thus, the transcriptional machinery may also play an important role in stress response pathways.

Several proteins associated with translation (Initiation factor IF2 VAS14_07334; NusG VAS14_19181; and the ribosomal proteins VAS14_19236 and VAS14_18779; Tables 1, 2) were found to be more abundant under UVB treatment than their respective dark controls, indicating that these elements play a pivotal role in the UVB response by modulating translation. An alternative role for ribosomal proteins as chaperones has been demonstrated in *E. coli*, in which they may ensure proper RNA and protein folding [52]. In this study, we determined that the ribosome releasing factor, a protein that participates in the ribosome pathway, was activated under UVB irradiation. The ribosome releasing factor (VAS14_20971, Table 2) is responsible for the release of ribosomes from mRNA after the termination of protein biosynthesis [53] and may increase the efficiency of translation by recycling ribosomes from one round of translation to another. This result suggests that the persistent activation of the ribosome pathway may play a key role in cellular responses to UVB radiation [54]. The active regulation of protein levels inside the cell is critical for the resistance of *P. angustum* under UVB radiation.

Taken together, the up-regulation of both transcription and translation factors and ribosomal proteins indicates that despite UVB stress conditions, cells successfully maintain the genetic information required for their successful survival.

### Protein folding and proteolysis

Three proteins involved in protein folding and processing, DnaK (VAS14_07124, Table 1), GroEL (VAS14_18941, Table 2) and a trigger factor (VAS14_06218, Table 1), exhibited increased abundance as a result of UVB treatment. The chaperones play critical roles in the folding of newly synthesized proteins and refolding of misfolded proteins. The increased level of these proteins in response to irradiation indicates that an important mechanism for coping with UV-induced stress in *P. angustum* is the maintenance of proper protein function (Figure 6). Previous studies have indicated a role for GroEL and DnaK in coping with UV-induced damage in both *E. coli* and mammalian skin cells [55], [56]. DnaK and GroEL regulate both sigma32 activity and degradation, thereby allowing sigma32 to indirectly sense unfolded proteins by sensing chaperone occupancy.

In contrast to chaperones that prevent protein unfolding or rescue unfolded proteins, proteolytic activity is important for processing irreversibly damaged or denatured proteins. We found three proteins (VAS14_18824, VAS14_06208, and VAS14_07364, Table 1) that shared similarities with proteases; of these, two proteases have not yet been functionally characterized. The FtsH (filamentation temperature sensitive) protein (VAS14_07364, Table 1) is an ATP-dependent zinc metallopeptidase. The FtsH gene of *Bacillus subtilis* has been identified as a general stress gene that is transiently induced after a thermal or osmotic upshift [57] and is essential for cell viability in *E. coli* [58]. The FtsH protein mediates proteolysis and is also involved in transport pathways, peroxisome assembly, the cell division cycle, gene expression [58], insertion of proteins into membranes and the disassembly/oligomerization of protein complexes [59]. Furthermore, FtsH was found to play a crucial role in degrading unneeded or damaged membrane proteins and also targets the cytoplasmic signaling factor sigma32 (σ^32^). The FtsH protease controls σ^32^stability [60], and the DnaK and GroEL chaperone machines control σ^32^ activity [60], [61], thus ensuring homeostatic control. However, the σ^32^ factor regulates the expression of heat shock promoters and overaccumulates in the absence of FtsH [60], [61]. Thus, it would not be surprising if the increased abundance of both protein chaperones (DnaK and GroEL) along with the FtsH protease were linked to the down-regulation of the heat/cold shock protein, as explained by a decrease in the abundance of the σ^32^ factor. In *P. angustum*, one heat shock protein (VAS14_14099) and two cold shock proteins (VAS14_ 02983 and VAS14_14614) were found to be down-regulated by UVB treatment (Figure 6). Because a large number of other stress conditions induce the HSPs, this response is often referred to as the universal stress response [62].

### Hypothetical proteins

Four of the 55 proteins of interest were identified as hypothetical proteins and found to be either down- (VAS14_02738, ∼1.70 fold, Table 1; VAS14_09609, 1.90 fold, Table 2) or up-regulated (VAS14_14319, ∼1.75 fold, Table 1; VAS14_ 08110, 1.60 fold, Table 2) under UVB radiation. These proteins could be quantified by both gel-free and gel-based methods and piqued our interest because they are fully hypothetical proteins with no conserved domains; they also exhibited greater than 1.5-fold changes in expression upon exposure to UVB radiation. Furthermore, two other putative proteins that contained conserved domains but had no associated functions were found to be up-regulated under UVB radiation (putative GTP binding protein, VAS14_16369, ∼1.5 fold, Table 1; putative lipoprotein, VAS14_11709, Table 3). The lipoprotein biomarker quantified using both quantitative proteomics methodologies exhibited various fold changes depending on the methodology (2.33 with post-digest ICPL and 1.33 with 2D-DIGE). As mentioned in the previous paragraph, the DIGE methodology allowed us to detect several isoforms of the putative lipoprotein, and further experiments are underway to characterize its function and understand why one isoform is more involved in the UVB response than the other. Finally, it would be valuable to perform host expression of the hypothetical proteins to assess their various functions and conclude if they participate actively in the UVB resistance of *P. angustum*.

### Comparison of the copiotrophic *versus* the oligotrophic bacterial response to UVB stress

Microorganisms have been classified into two main trophic lifestyles based upon their environment and main physiological traits. Copiotrophic microorganisms such as *P. angustum* are specialized for life in nutrient-enriched habitats of the sea, while oligotrophic bacteria are specifically adapted to life in low-nutrient environments [63], [64]. A recent study has highlighted the key differences in the genomes of two marine bacteria: *Sphingopyxis alaskensis* RB22456, an abundant oligotrophic alpha-proteobacterium, *versus* the copiotrophic gamma-proteobacterium *P. angustum* S14 [9]. Given that UVB penetrates deeper into clear oligotrophic open oceans compared with eutrophic coastal and estuarine waters [65], it would not have been surprising to observe a greater level of resistance in oligotrophic bacteria, which are hypothetically better adapted to cope with UVB than copiotrophic prokaryotes. Nevertheless, we previously reported that the UVB response of these bacteria, which have evolved two entirely different trophic lifestyles, was more subtle than first expected. We demonstrated that *P. angustum* and *S. alaskensis* may have very distinct strategies for resistance to UV radiation. Although *S. alaskensis* presented a very efficient photoenzymatic repair system, no significant evidence of overexpression of proteins directly involved in DNA repair (*i.e.*, photolyase) was observed in the proteomics approach. Indeed, two proteins involved in DNA repair (DNA repair RadA: Sala_1806; DNA repair RecN: Sala_0546) were identified in the expressed proteome, but these proteins did not have significant differential abundance under any of the conditions tested [8]. In contrast to *S. alaskensis*, the RecA protein was found to play a key role in the UVB resistance of *P. angustum* and characterized as one of the UVB biomarkers. This is one of the most striking differences between the UV responses of these bacteria. Both bacteria showed an active response regarding oxidative stress defense, and several key proteins, including thioredoxin/glutaredoxin, glyoxalase, glutathione, the antioxidant Ahpc, and several chaperonin proteins (DnaK, GroES/EL, and the trigger factor), were up-regulated. When *S. alaskensis* was treated with UVB, iron and nitrogen homeostasis appeared to play a more important role than in UVB-treated cells of *P. angustum. P. angustum*'s response to UVB involved a larger number of transporters than the response of *S. alaskensis*. Interestingly, it has been previously reported that copiotrophs have a larger proportion of extracytoplasmic proteins (i.e., cytoplasmic membrane, periplasmic, outer membrane, and extracellular proteins) and more diverse array of outer membrane proteins compared with oligotrophic bacteria [9]. *S. alaskensis* may have selected a strategy of protection as opposed to the efficient strategies for DNA damage removal and replacement of cellular components selected in *P. angustum*. Moreover, in addition to its very efficient DNA repair mechanisms and oxidative stress protection system, *P. angustum* exerts tight control over the structure of its cell membrane and could conserve extra energy for UV resistance by diverting energy away from the maintenance of general metabolism. Finally, it should be noted that several hypothetical proteins must be functionally characterized to further explore the mechanisms of UVB resistance in *P. angustum*; members of this pool of proteins could have some remarkably interesting roles.

### Conclusion

The use of a combined proteomic analysis has allowed us to identify a set of biomarkers of the UVB stress response in *P. angustum*. We have characterized the RecA protein, which is responsible for efficient DNA damage removal under UVB radiation in *P. angustum*. We have observed the deployment of membrane proteins that may facilitate nutrient exchange and stabilize the structure of the cell membrane in *P. angustum*. Transcription and translation were both tightly regulated, along with RNA and protein chaperones; this allows for the replacement of damaged cellular components and proper RNA and protein folding. The machinery associated with oxidative repair and protection was also found to be important in UVB-stressed cells of *P. angustum*. This proteomics study has allowed us to explore both the technical aspect of quantitative proteomics methodology and diverse UVB response strategies of two well-studied marine microorganisms.

## Supporting Information

Figure S1 Annotated tandem mass spectra.
(PDF)

Figure S2 The % variation for the common proteins (295) from the two technical replicates.
(TIF)

Figure S3 Alignment of the RecA protein sequence from *P. angustum* with its homolog from *E. coli*. Boxed residues indicates similar amino acids that are recognized by the primary antibody against RecA.
(TIF)

Figure S4 2D-DIGE experimental workflow from seven replicates. The three landmarks used for gels matching are indicated in blue, for the seven standard gels.
(TIF)
