# Supplementary material for: Proteome Analysis of the UVB-Resistant Marine Bacterium Photobacterium angustum S14
Source: PLoS One. 2012 Aug 1;7(8):e42299. doi: 10.1371/journal.pone.0042299 (PMC3411663; doi:10.1371/journal.pone.0042299)

REPLICATE 1

VAS14_ 00681 (score 98)

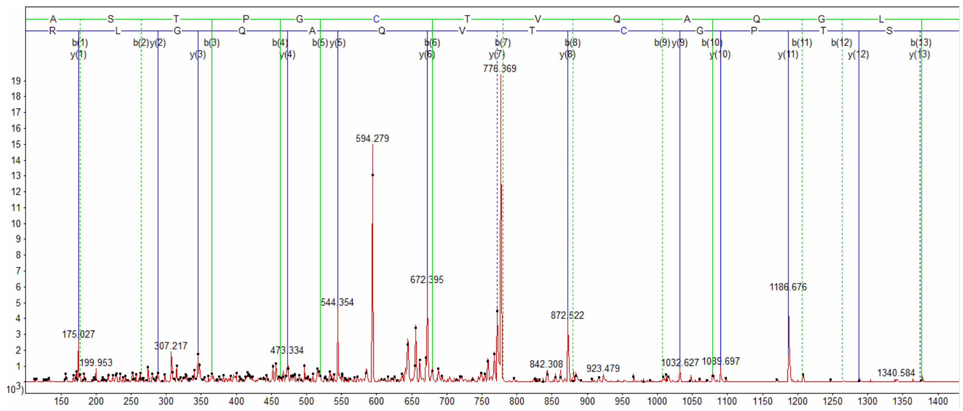

VAS14_18354 (score 97)

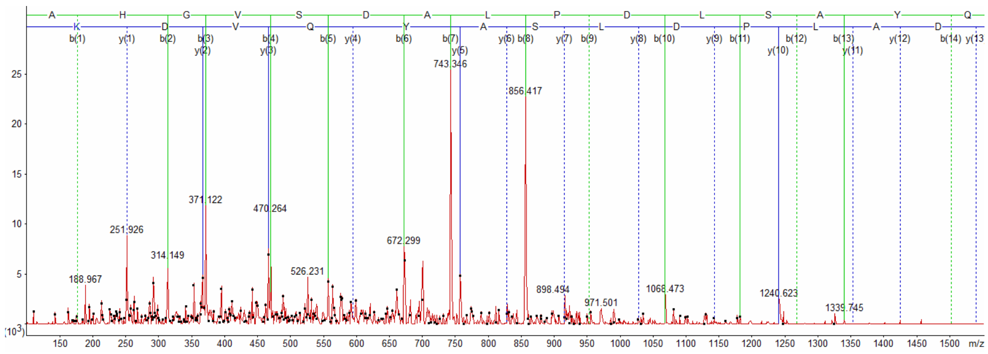

VAS14_17676 (score 91)

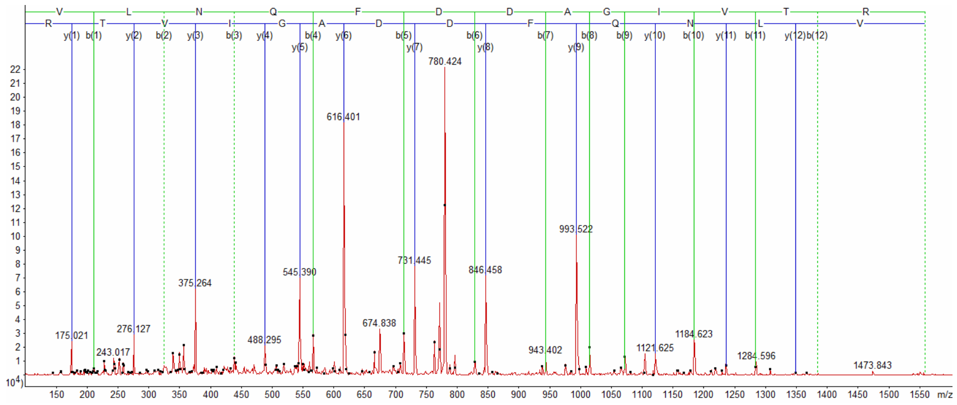

VAS14_ 01671 (score 73)

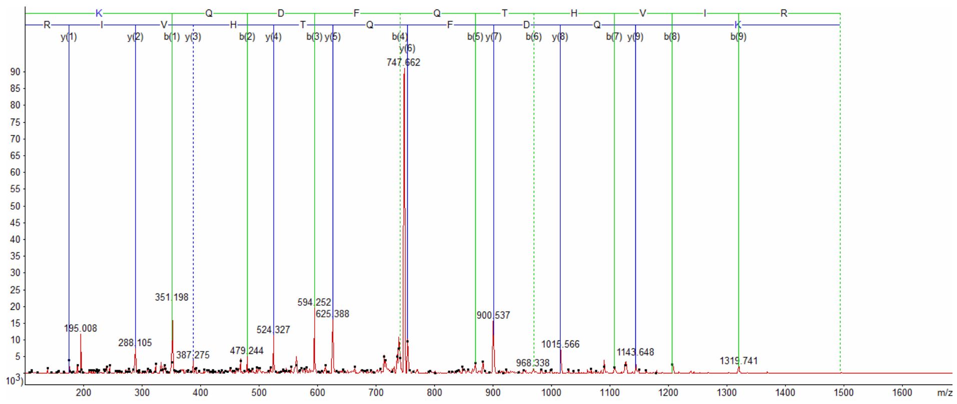

VAS14_16741 (score 72)

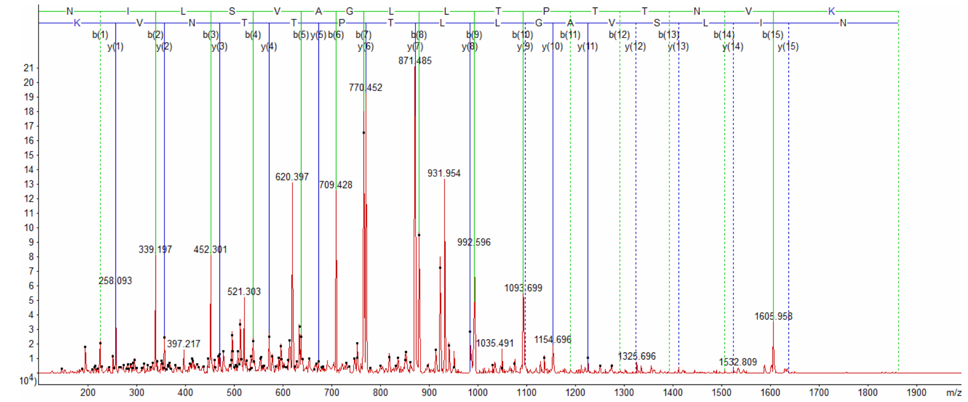

VAS14_16496 (score 70)

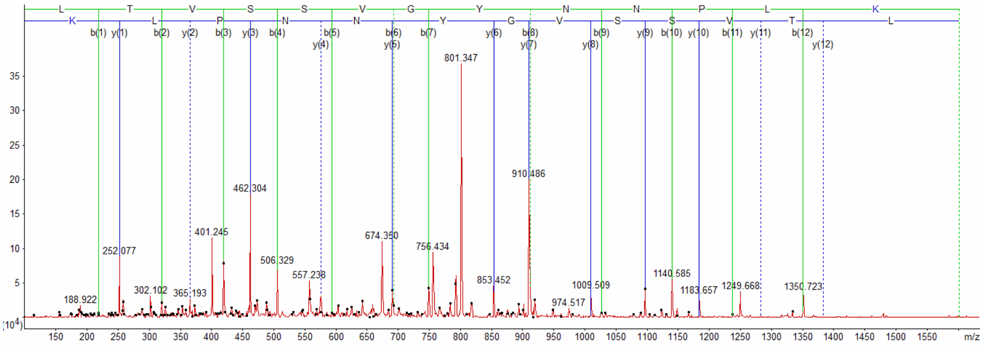

VAS14_08455 (score 66)

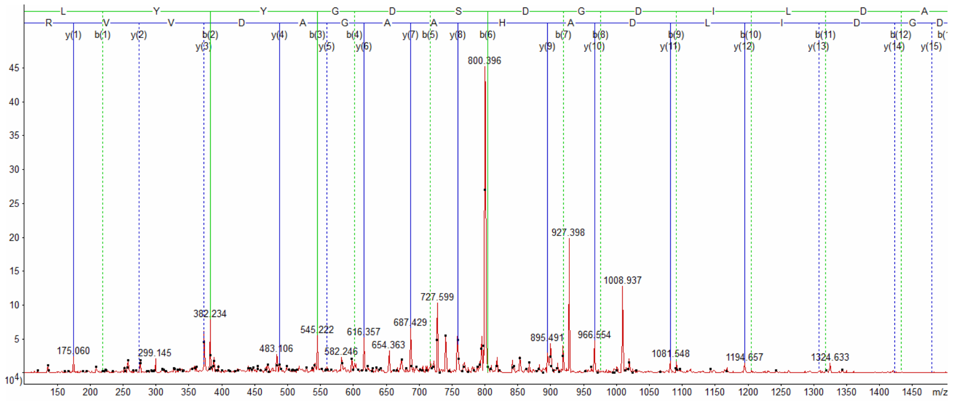

VAS14_ 07819 (score 64)

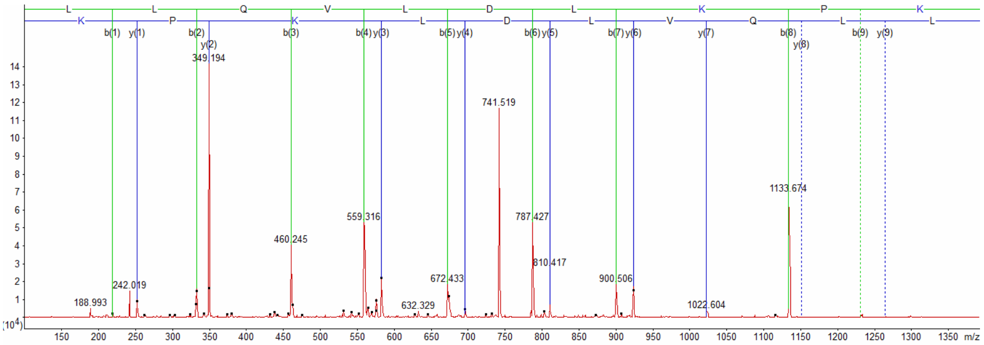

VAS14_19851 (score 59)

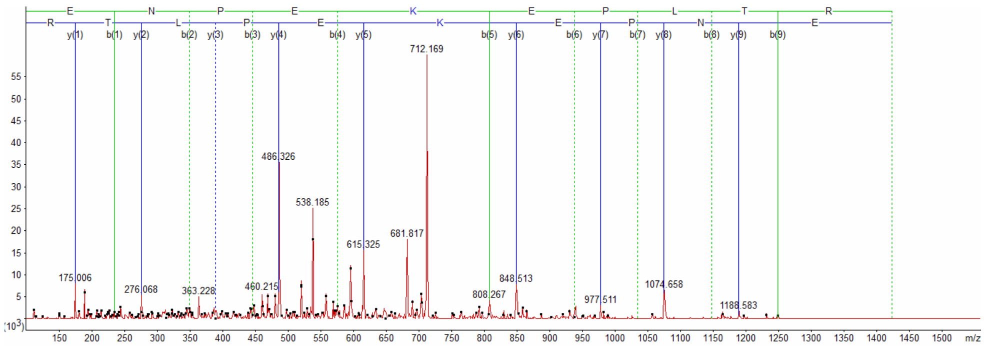

VAS14_06678 (score 58)

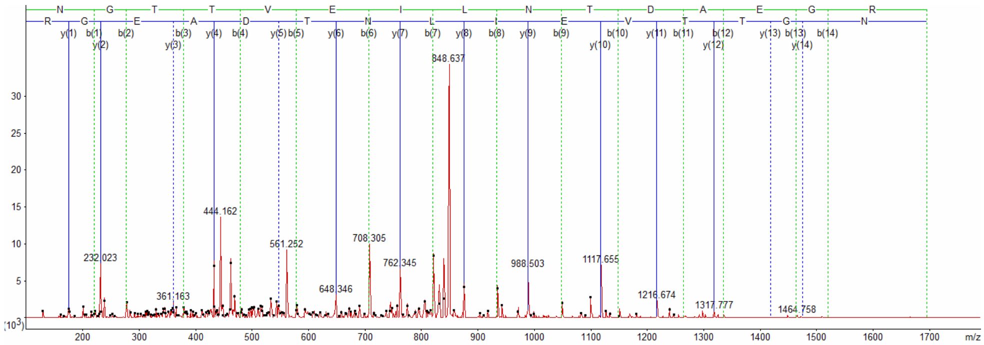

VAS14_18729 (score 53)

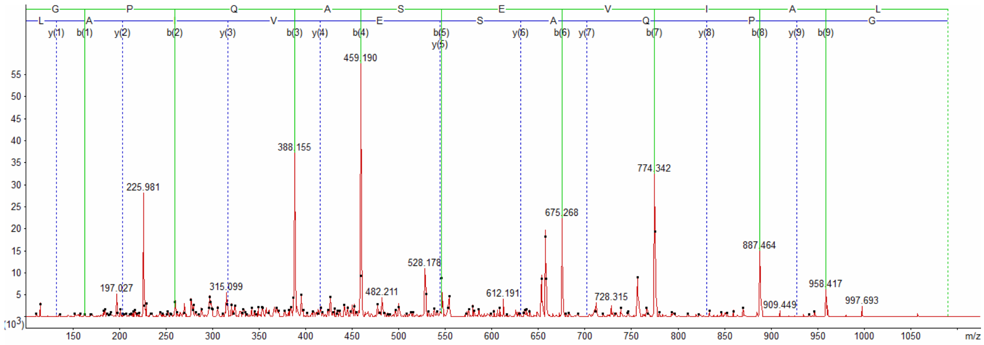

VAS14_21837 (score 52)

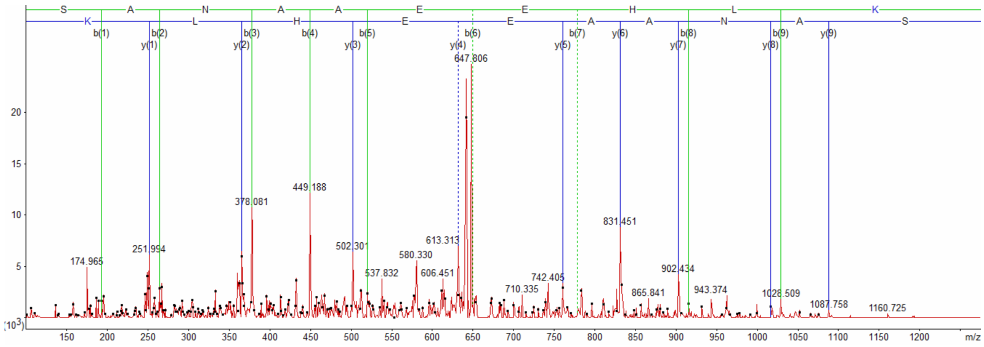

VAS14_07509 (score 52)

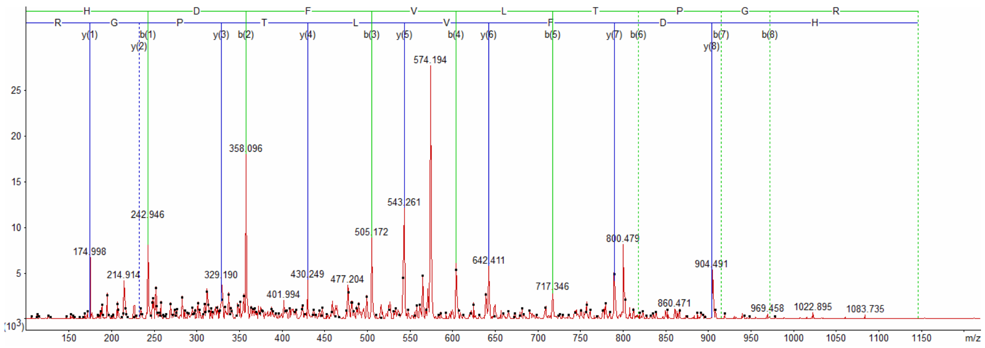

VAS14_17636 (score 52)

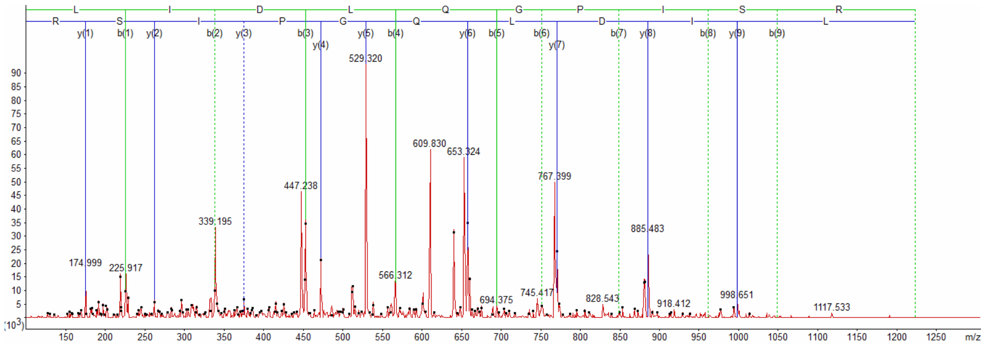

VAS14_05243 (score 51)

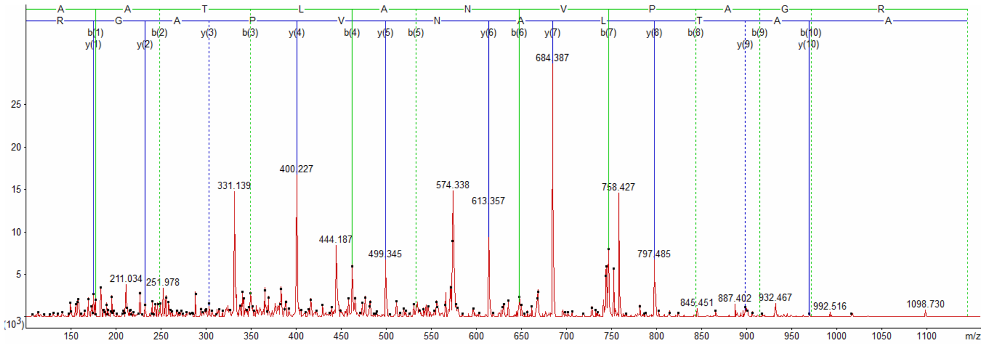

VAS14_07319 (score 40)

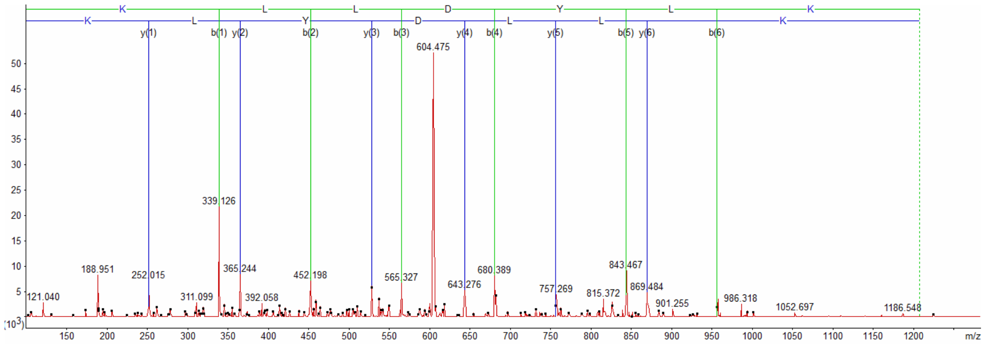

REPLICATE 2

VAS14_20261 (score 76)

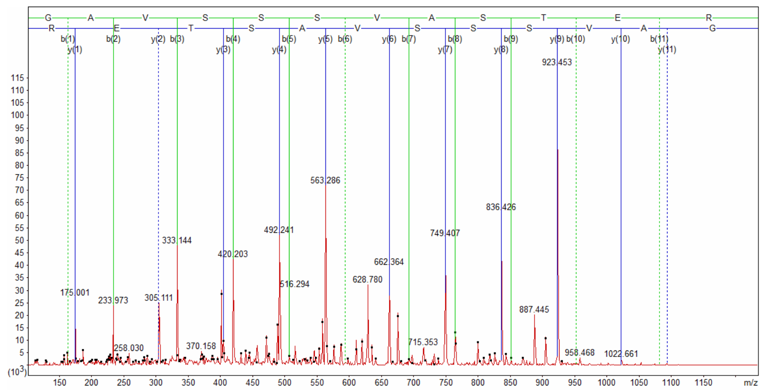

VAS14_18359 (score 76)

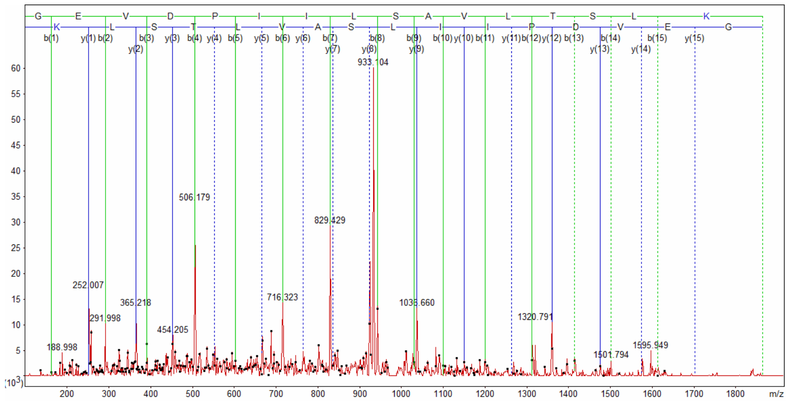

VAS14_07945 (score 74)

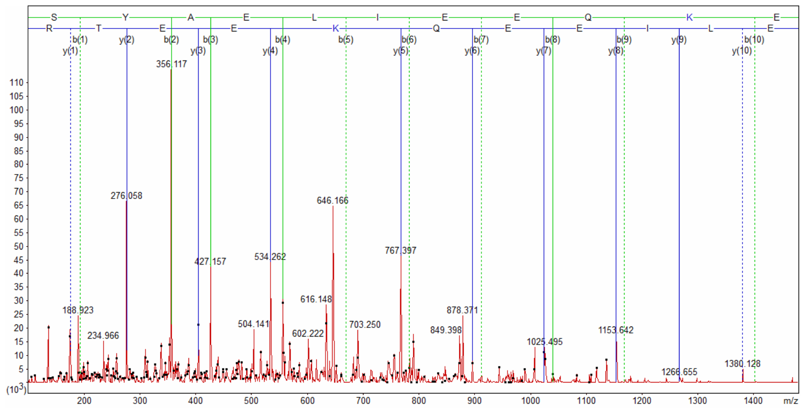

VAS14_17236 (score 74)

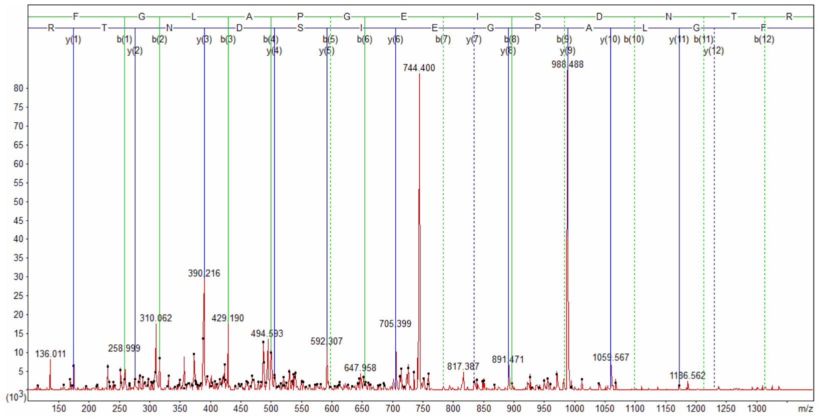

VAS14_12249 (score 73)

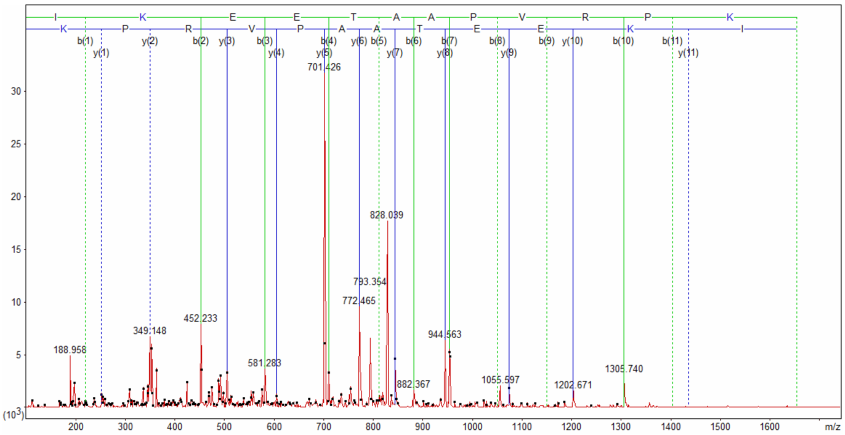

VAS14_11649 (score 73)

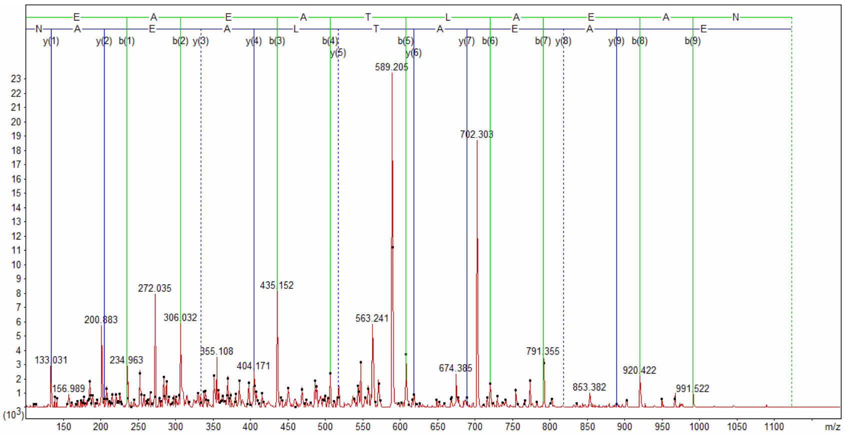

VAS14_07424 (score 70)

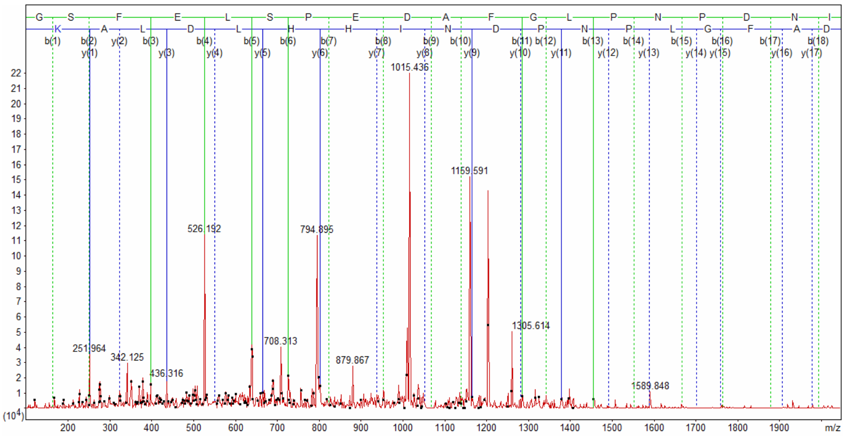

VAS14_07719 (score 68)

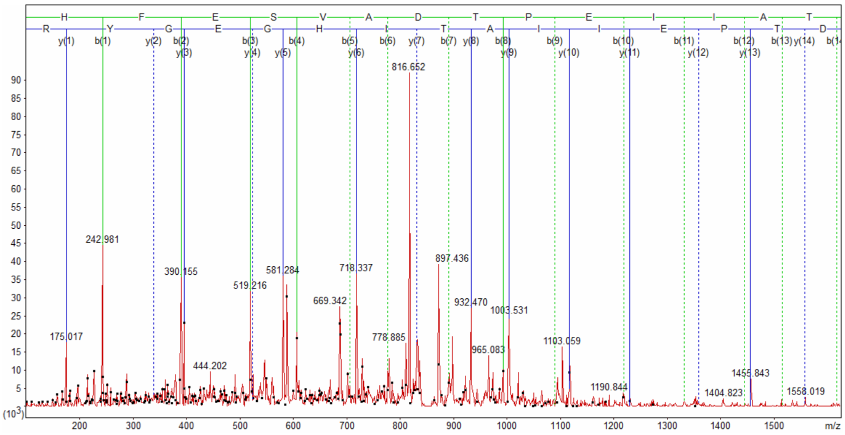

VAS14_00546 (score 68)

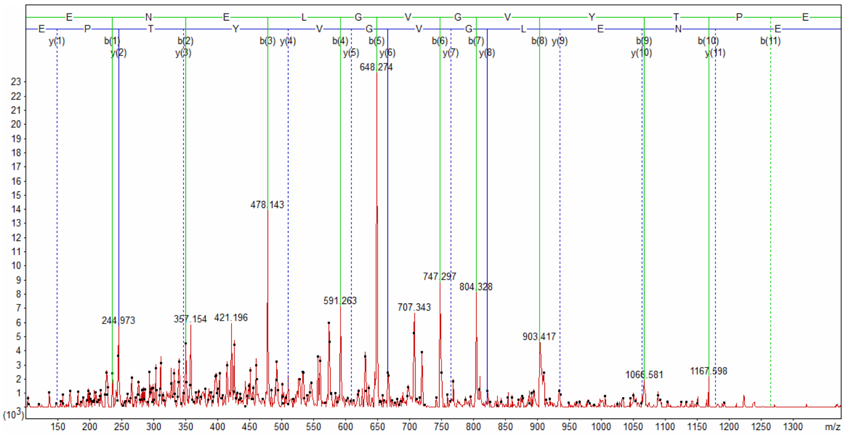

VAS14_06113 (score 67)

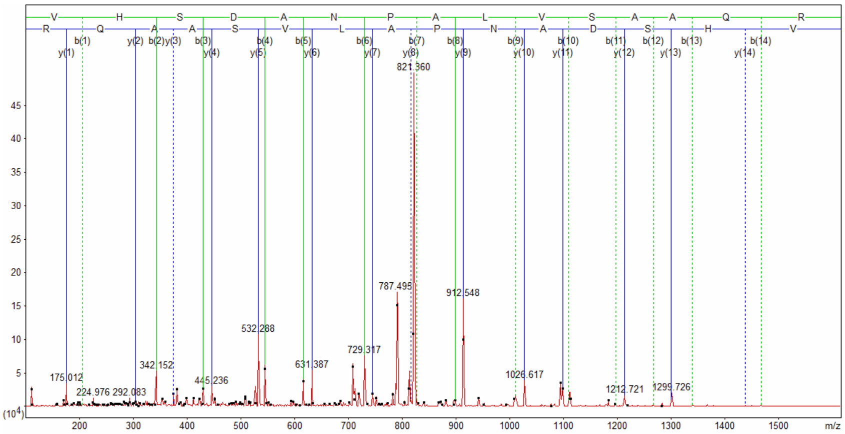

VAS14_21772 (score 66)

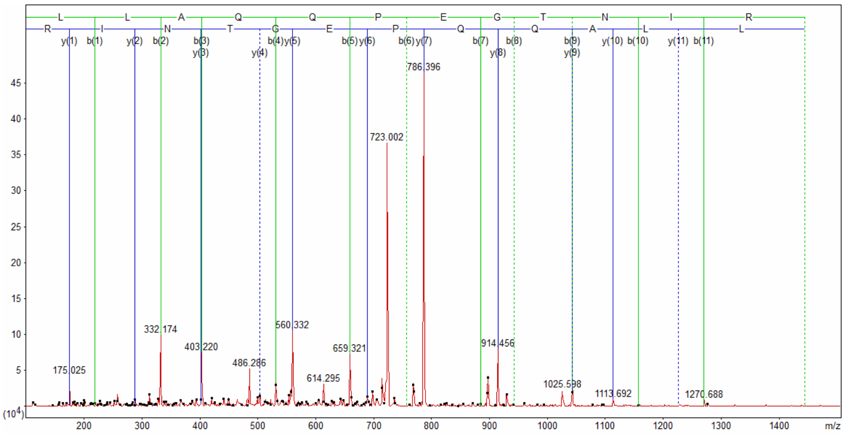

VAS14_04213 (score 65)

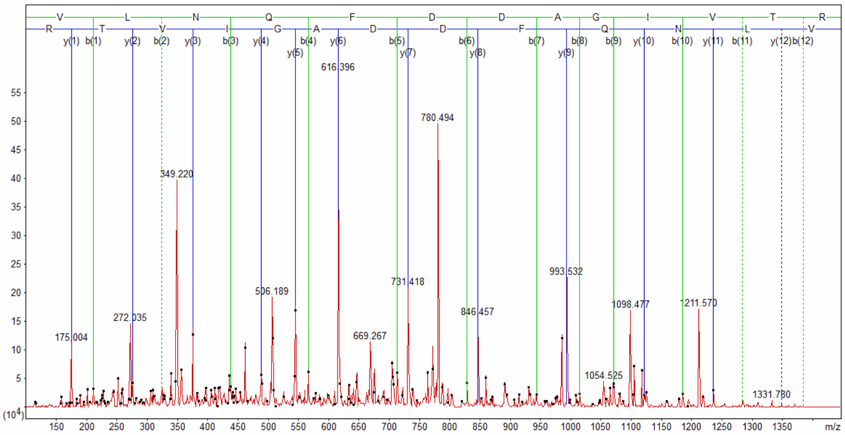

VAS14_ 18669 (score 64)

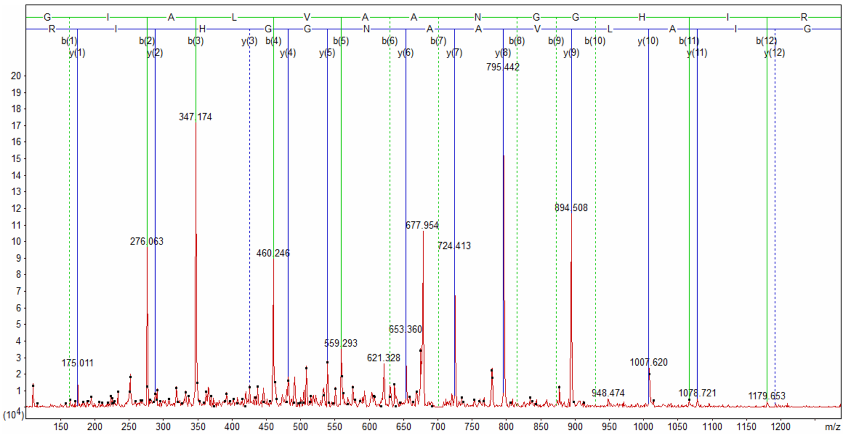

VAS14_ 03748 (score 63)

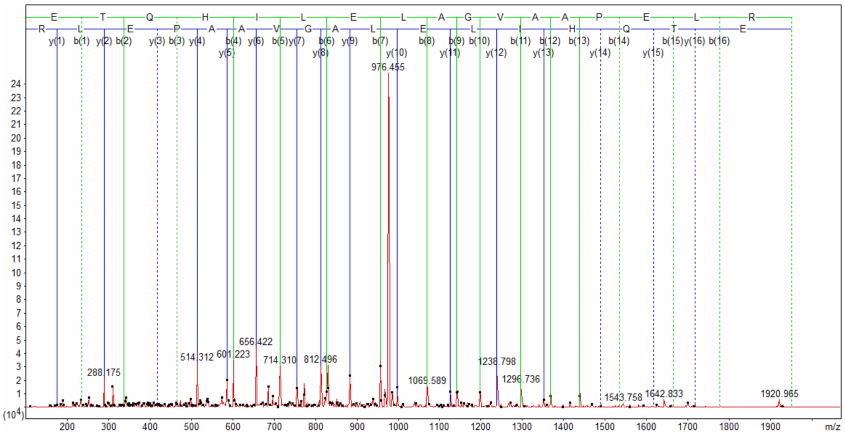

VAS14_19101 (score 62)

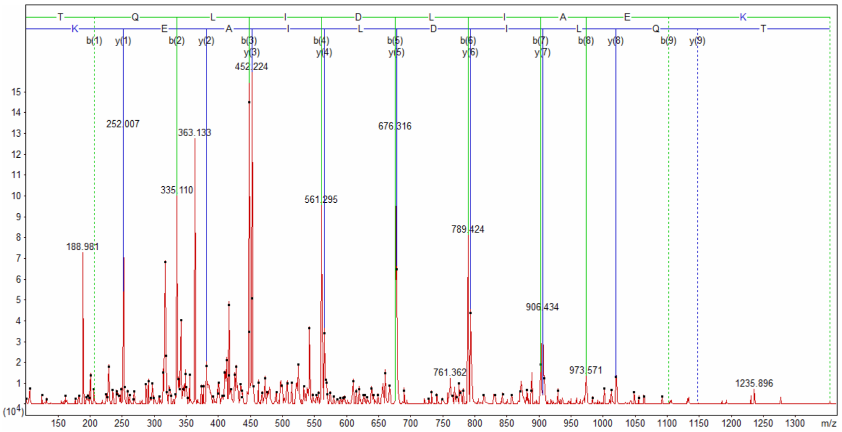

VAS14_ 02738 (score 61)

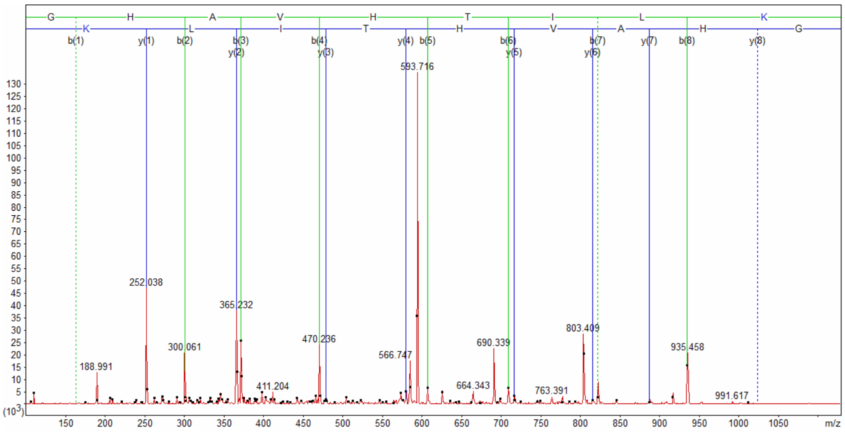

VAS14_10579 (score 60)

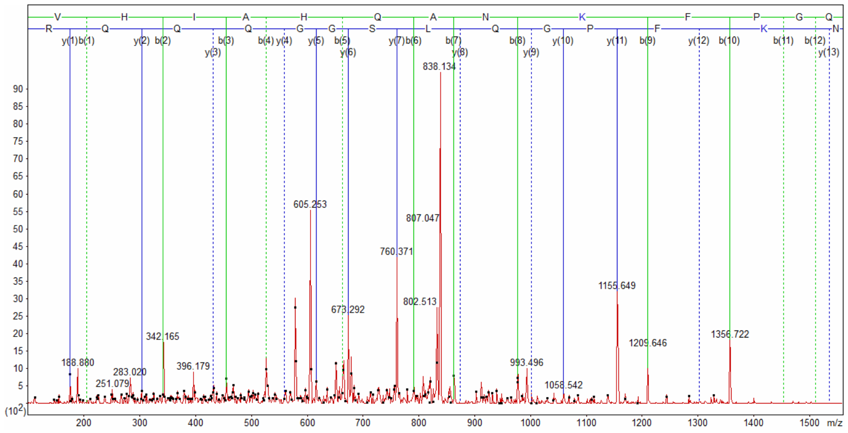

VAS14_18354 (score 56)

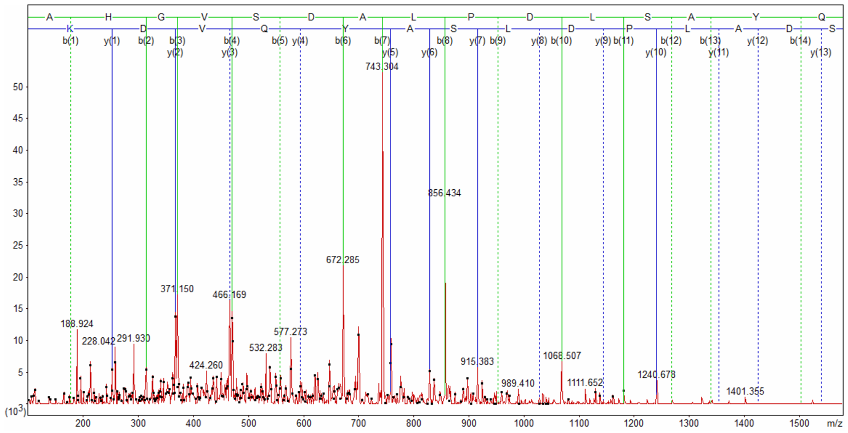

VAS14_01231 (score 56)

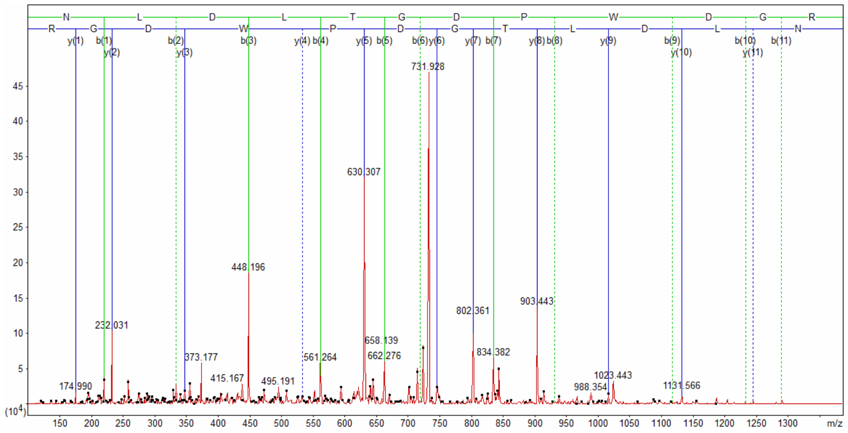

VAS14_ 13819 (score 55)

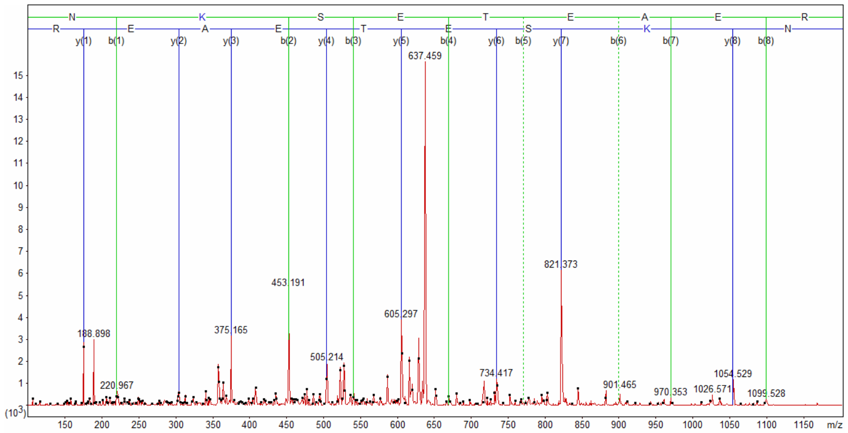

VAS14_18399 (score 55)

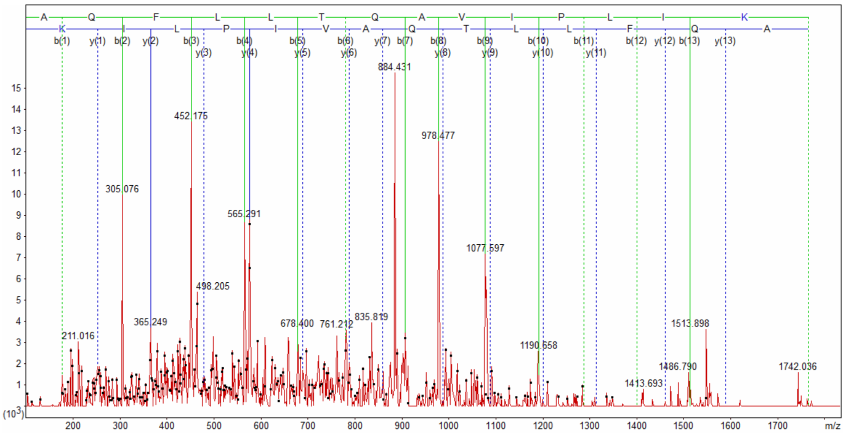

VAS14_19761 (score 54)

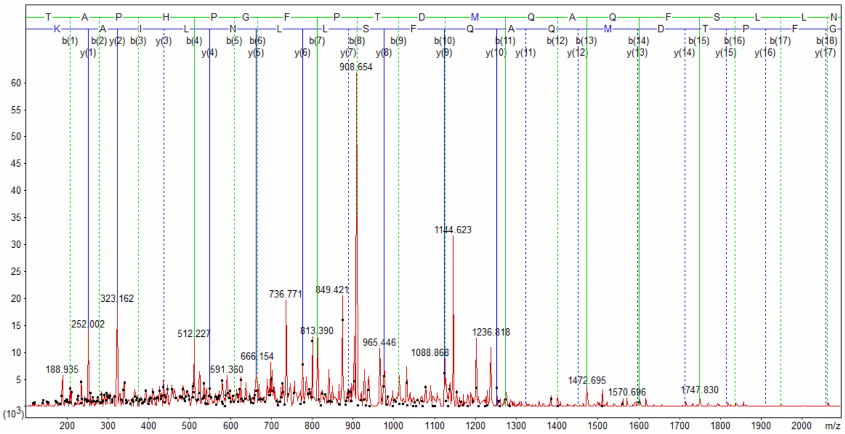

VAS14_19581 (score 54)

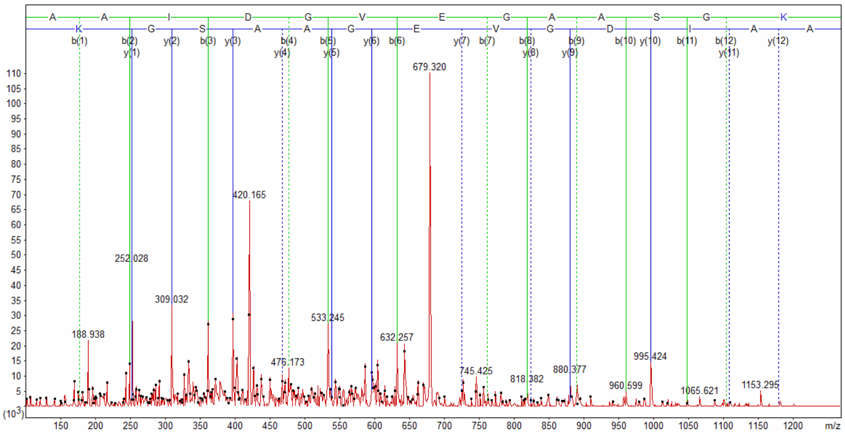

VAS14_07344 (score 54)

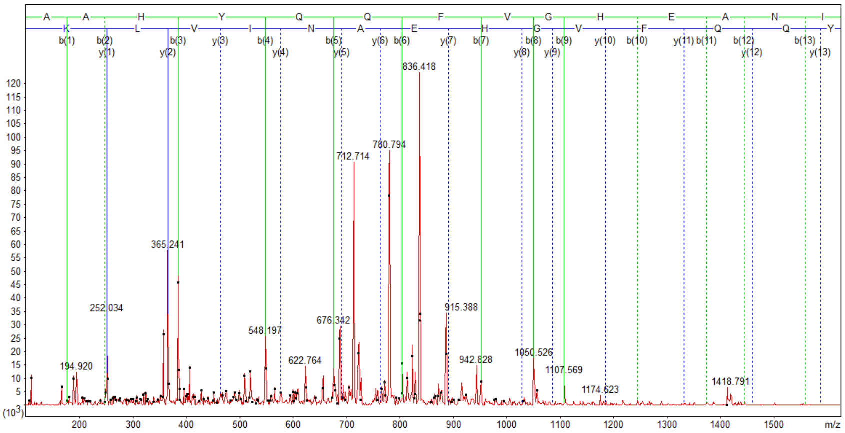

VAS14_12919 (score 52)

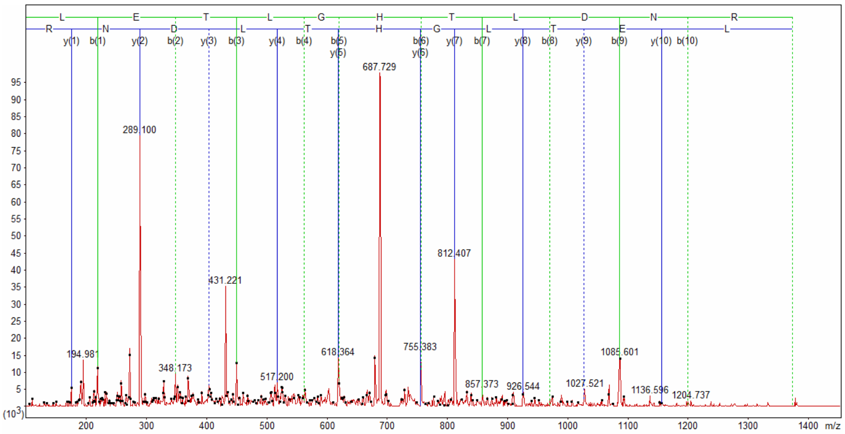

VAS14_19666 (score 52)

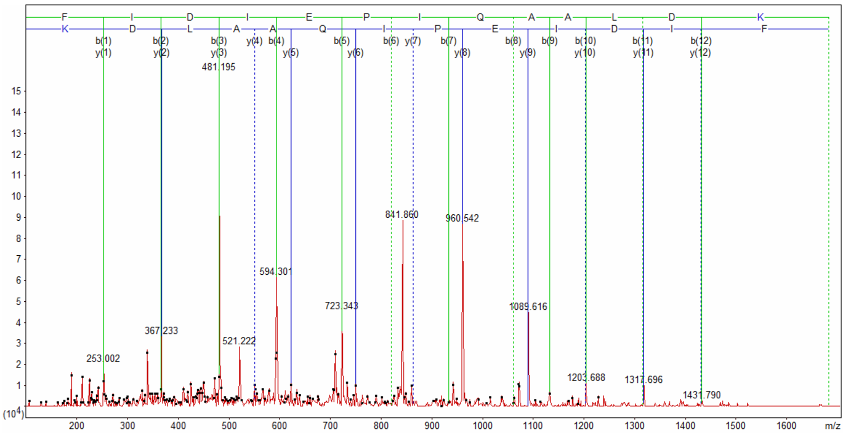

VAS14_20371 (score 50)

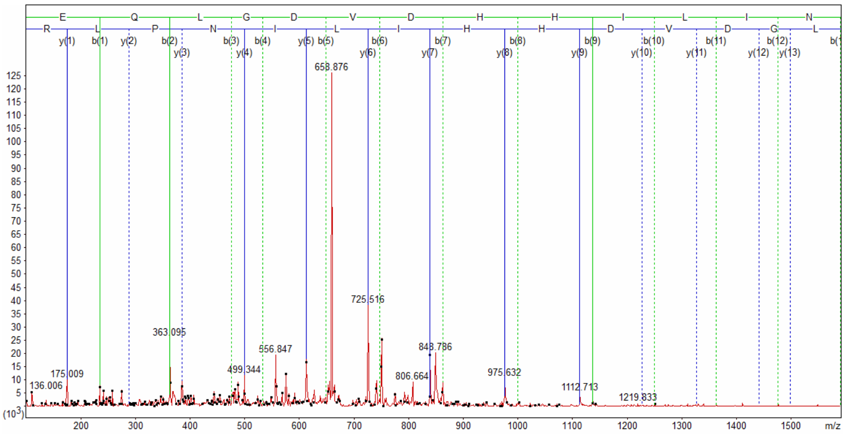

VAS14_17911 (score 47)

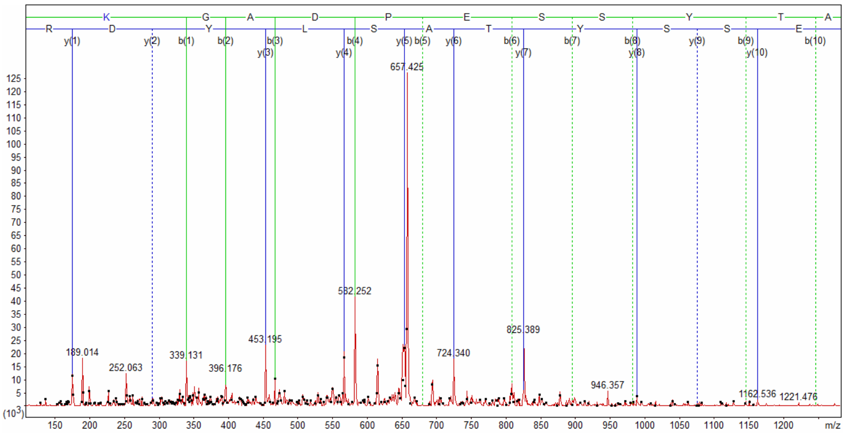

VAS14_01731 (score 47)

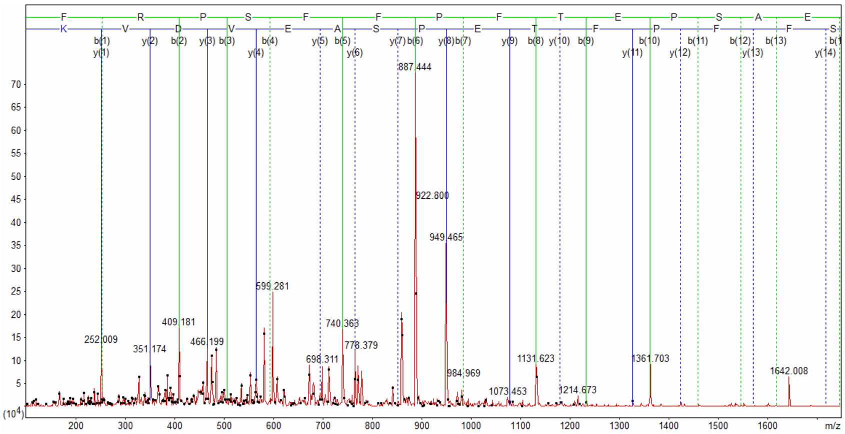

VAS14_16601 (score 46)

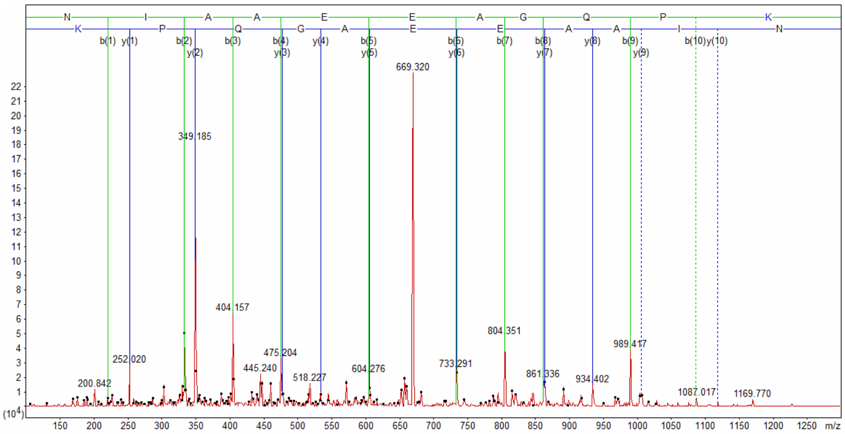

VAS14_ 21572 (score 44)

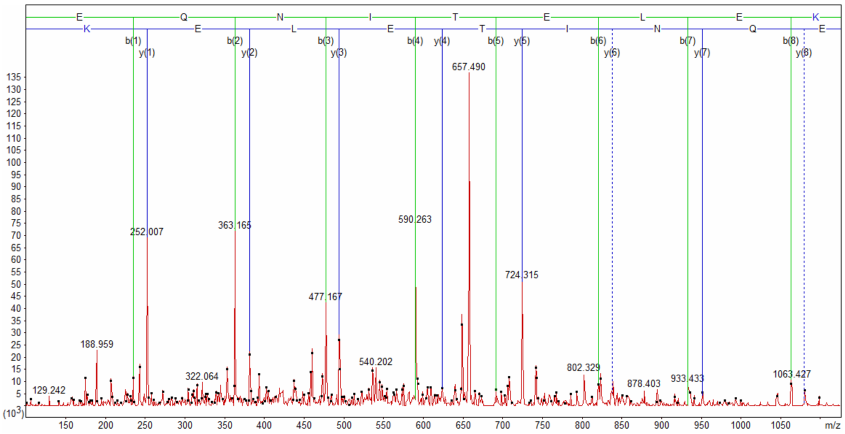

VAS14_00976 (score 42)

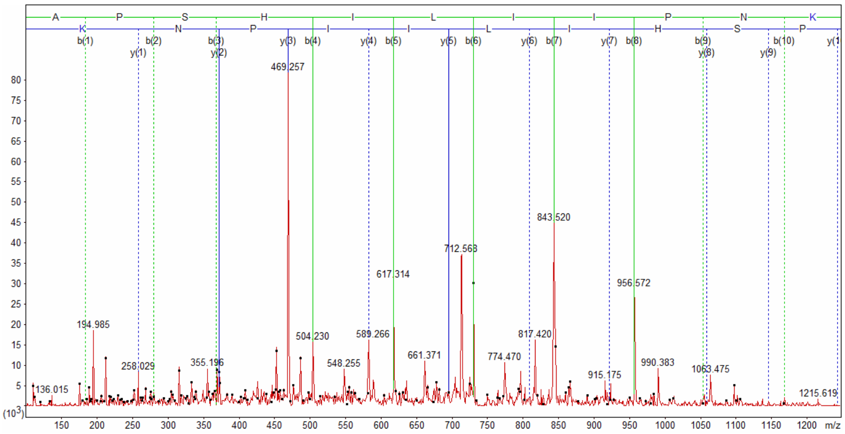

VAS14_16439 (score 42)

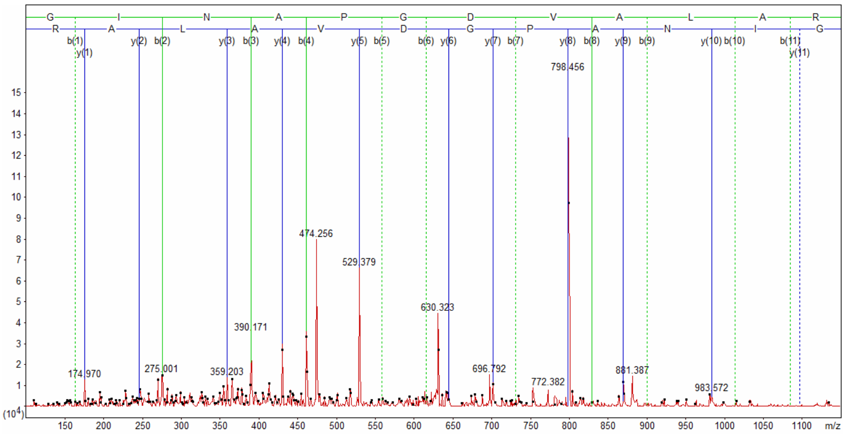

VAS14_00443 (score 41)

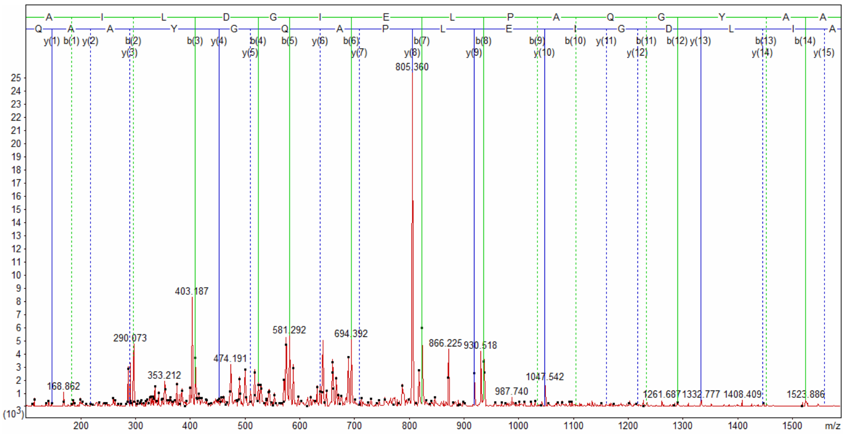

VAS14_21302 (score 41)

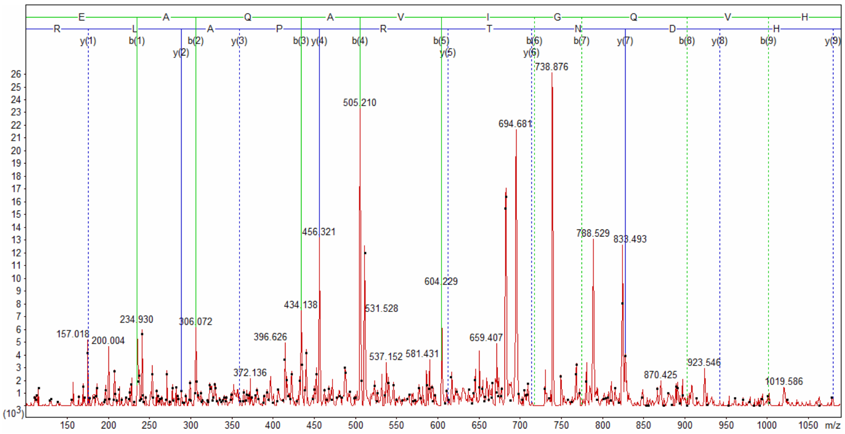

VAS14_21282 (score 40)

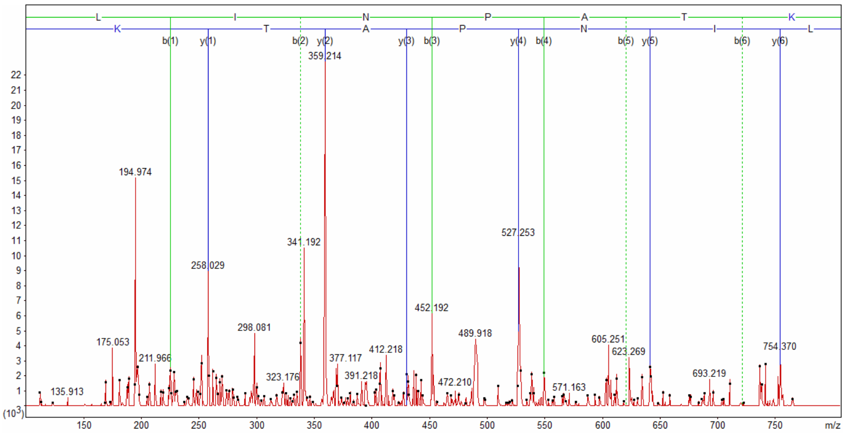

VAS14_19141 (score38)

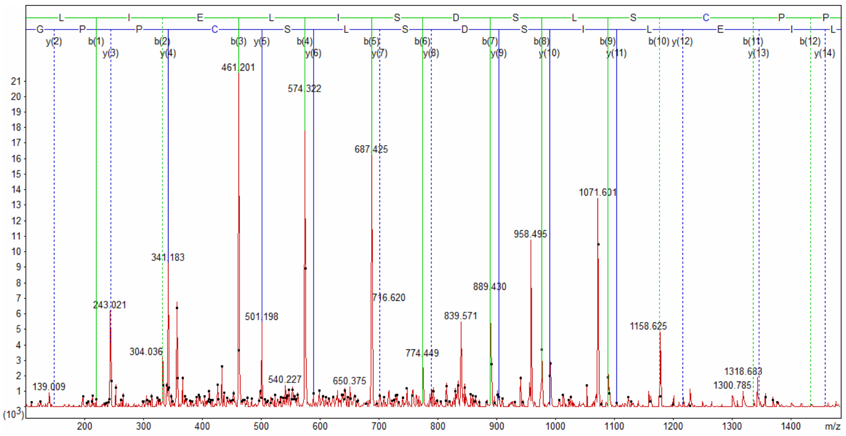

REPLICATE 3

VAS14_ 10609 (score 143)

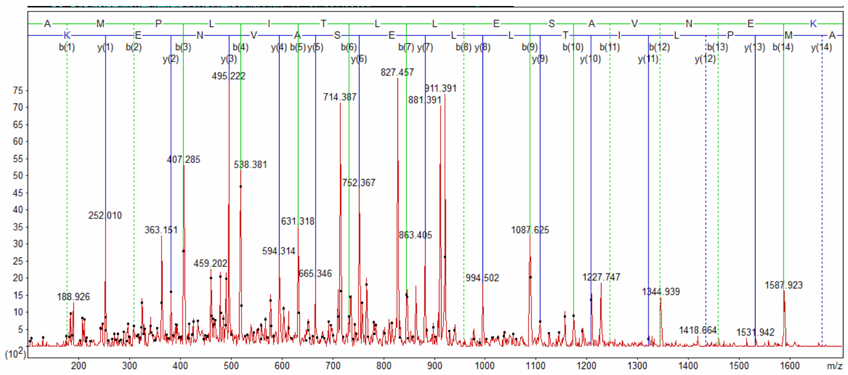

VAS14_ 07324 (score 122)

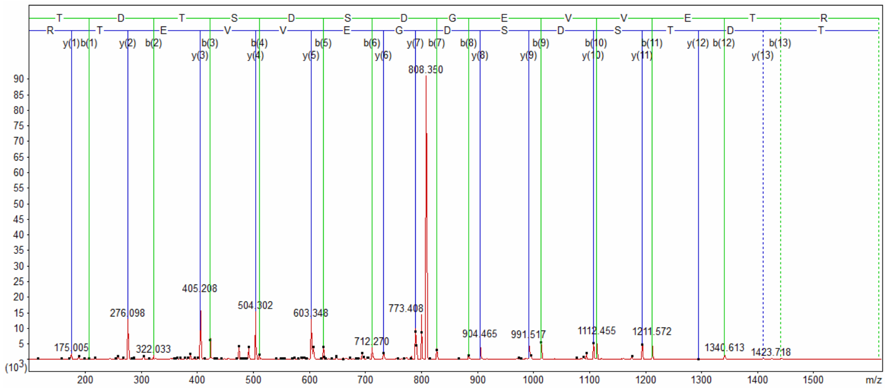

VAS14_ 03748 (score 121)

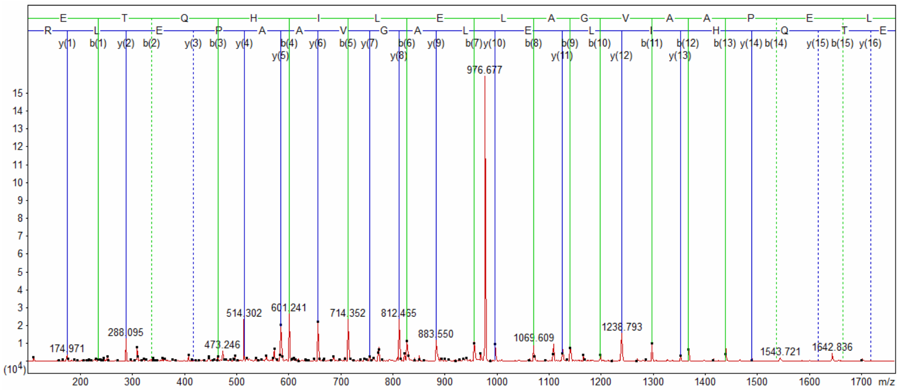

VAS14_22452 (score 112)

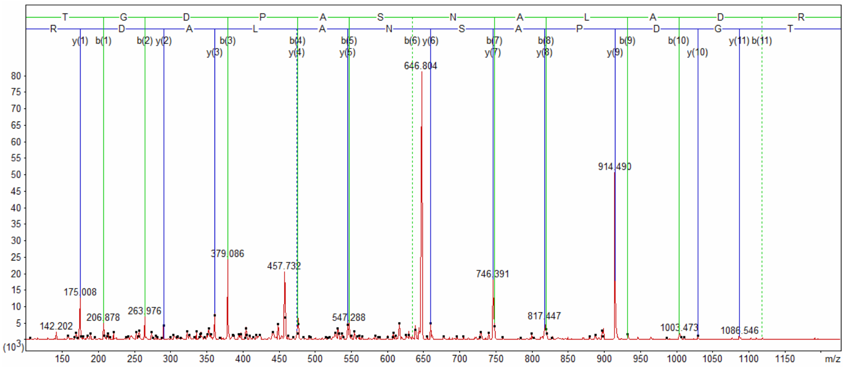

VAS14_19511 (score 101)

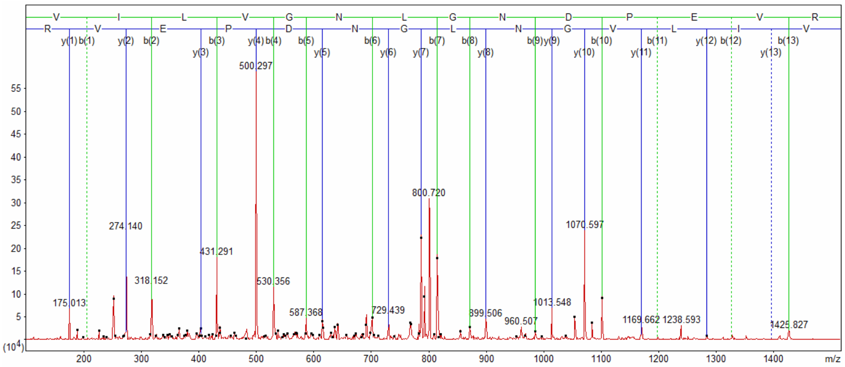

VAS14_21467 (score 109)

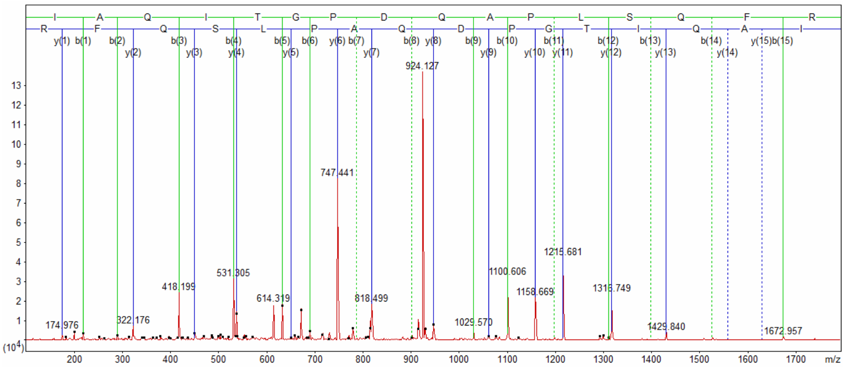

VAS14_00951 (score 104)

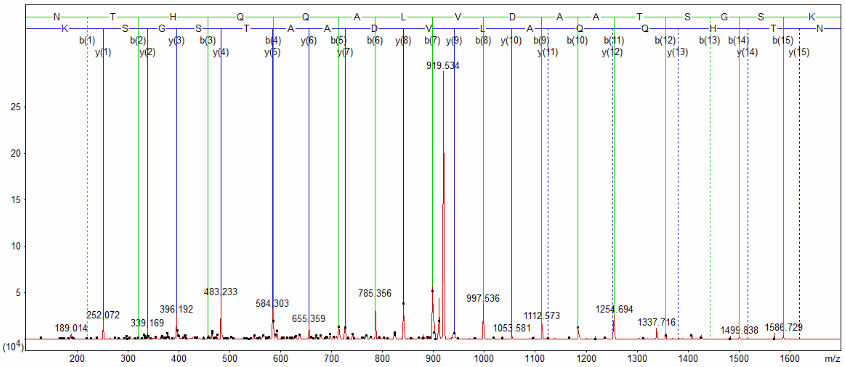

VAS14_07169 (score 99)

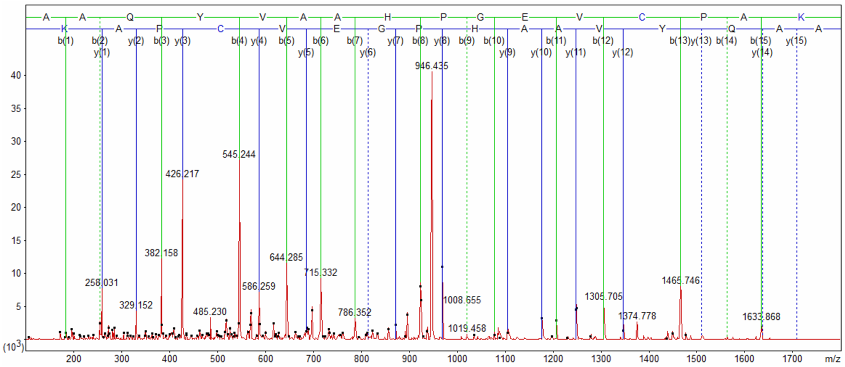

VAS14_ 09464 (score 95)

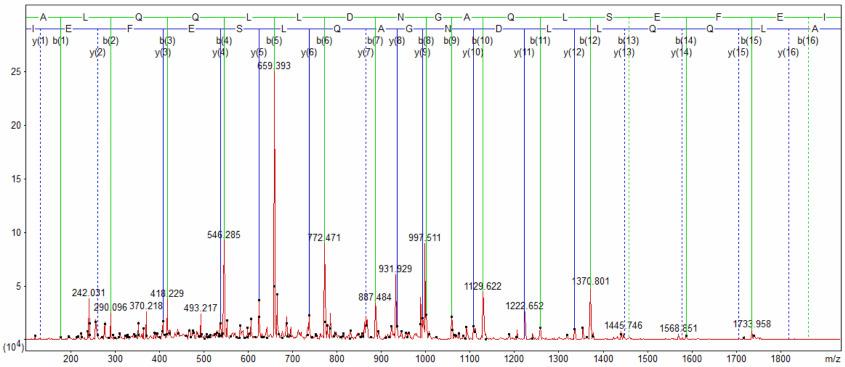

VAS14_00448 (score 93)

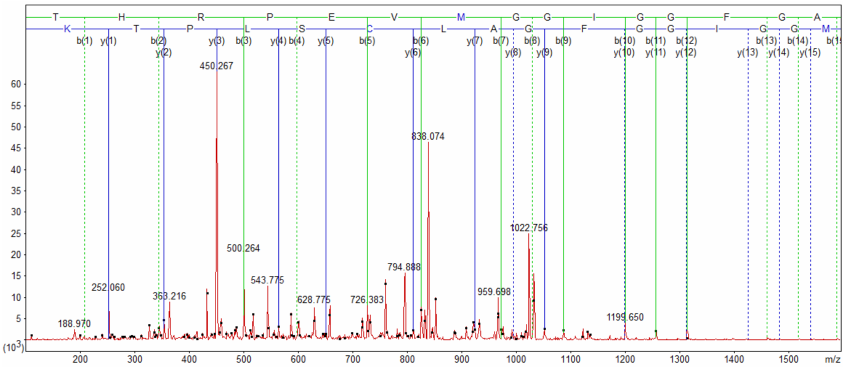

VAS14_19086 (score 93)

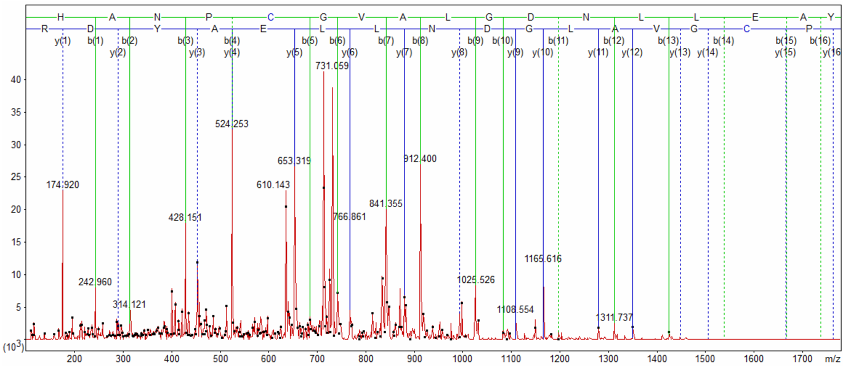

VAS14_12979 (score 87)

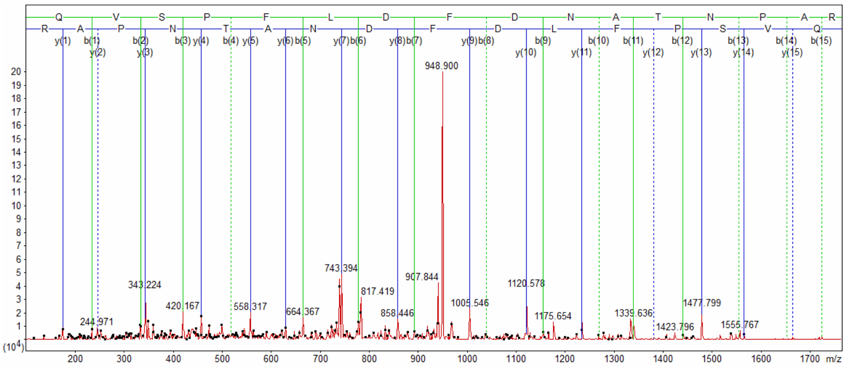

VAS14_18931 (score 79)

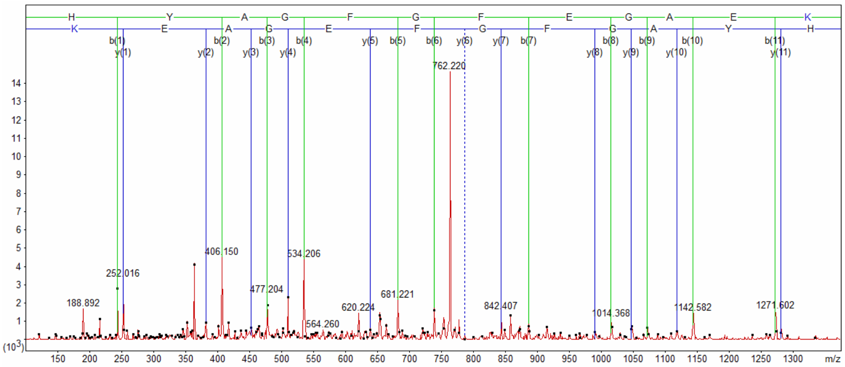

VAS14_ 18834 (score 78)

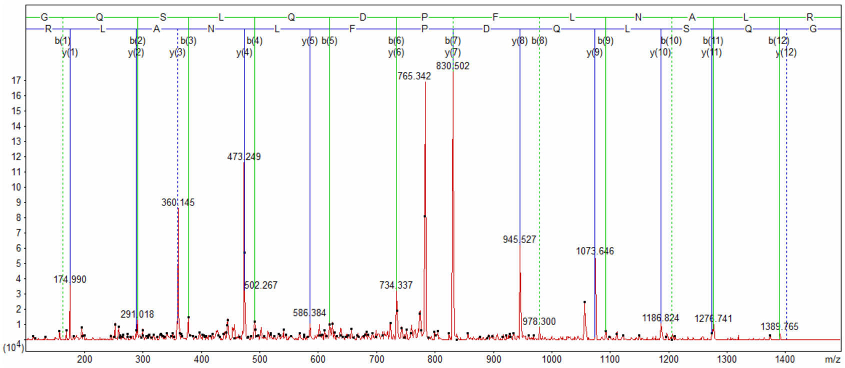

VAS14_19301 (score 73)

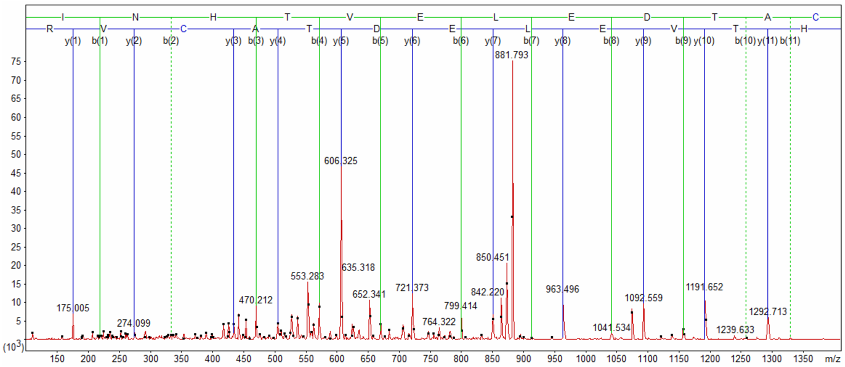

VAS14_19141 (score 70)

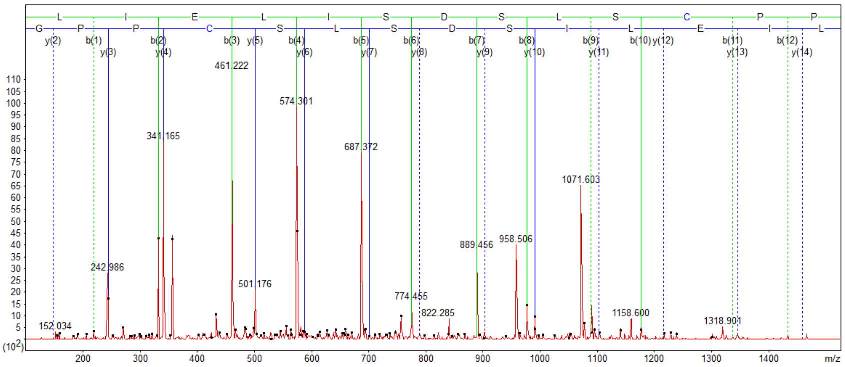

VAS14_02266 (score 69)

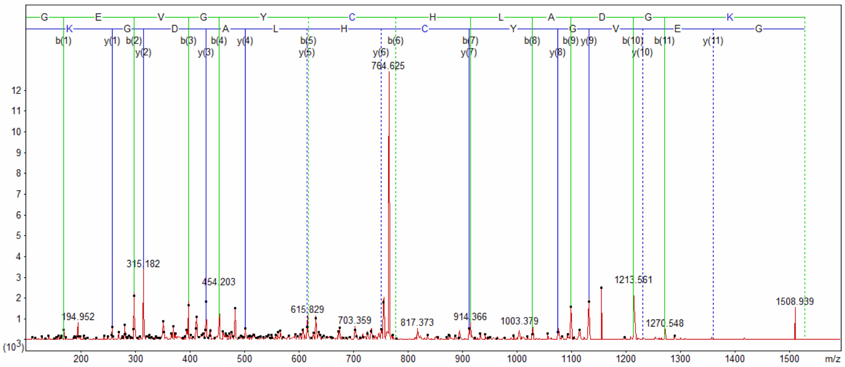

VAS14_11504 (score 69)

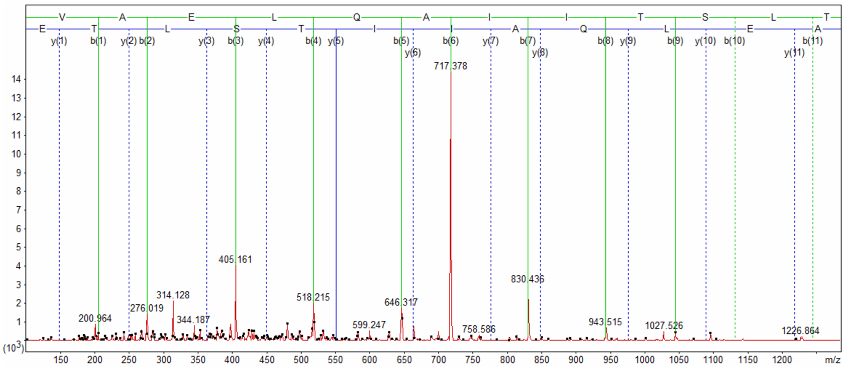

VAS14_05238 (score 66)

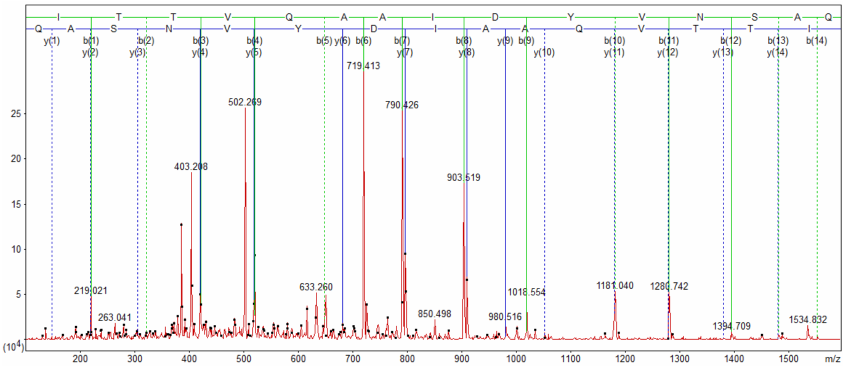

VAS14_ 18194 (score 66)

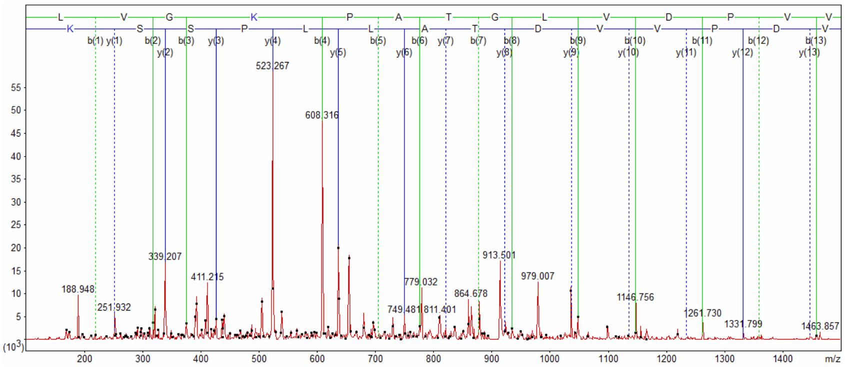

VAS14_10544 (score 66)

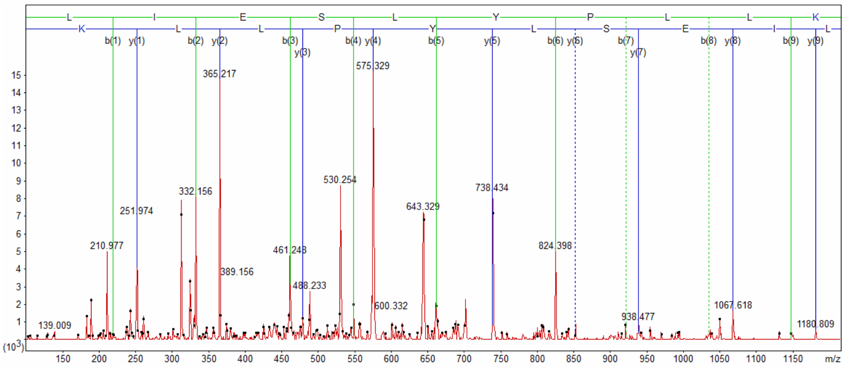

VAS14_11494 (score 65)

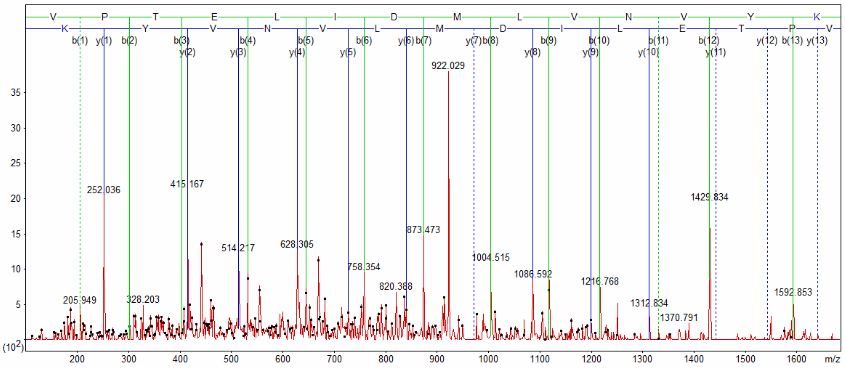

VAS14_ 17636 (score 64)

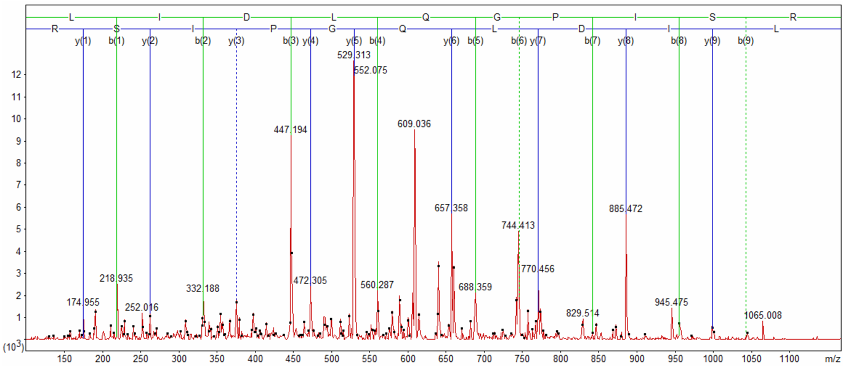

VAS14_16601 (score 64)

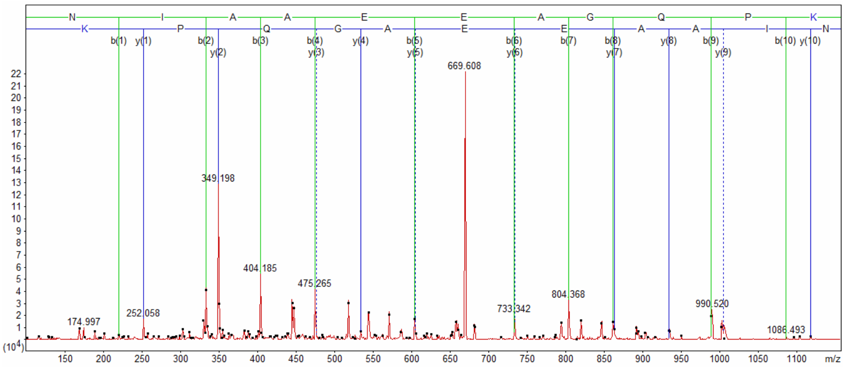

VAS14_21772 (score 63)

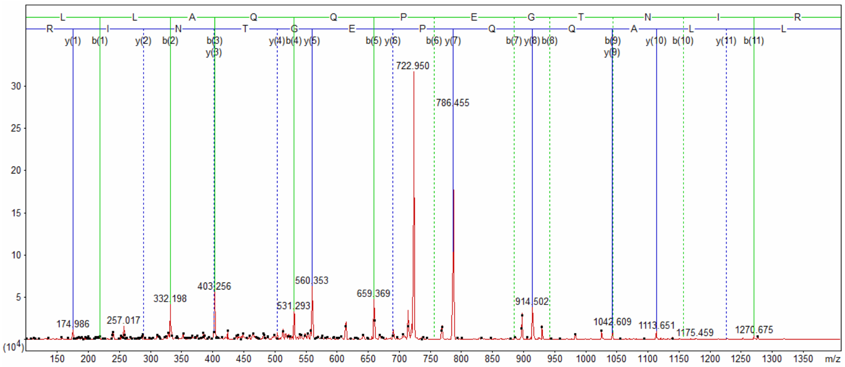

VAS14_ 20171(score 62)

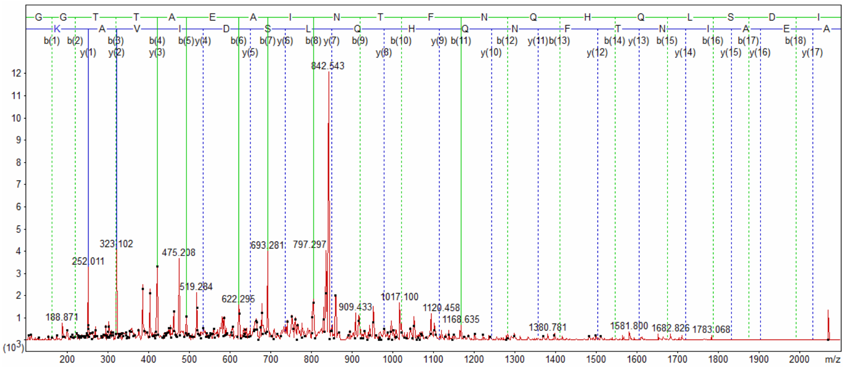

VAS14_05413 (score 60)

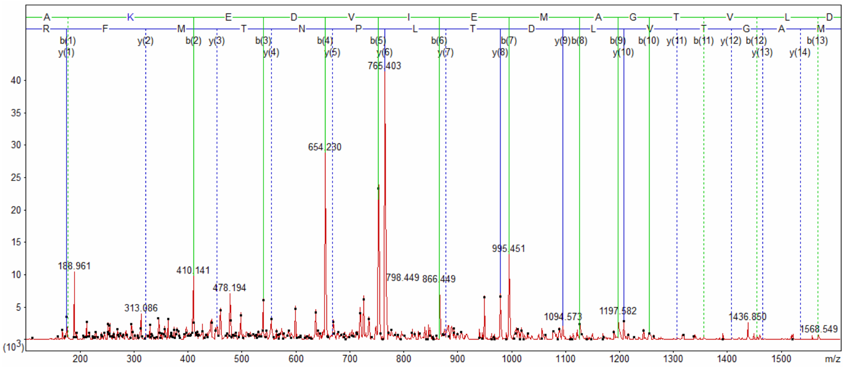

VAS14_06918 (score 59)

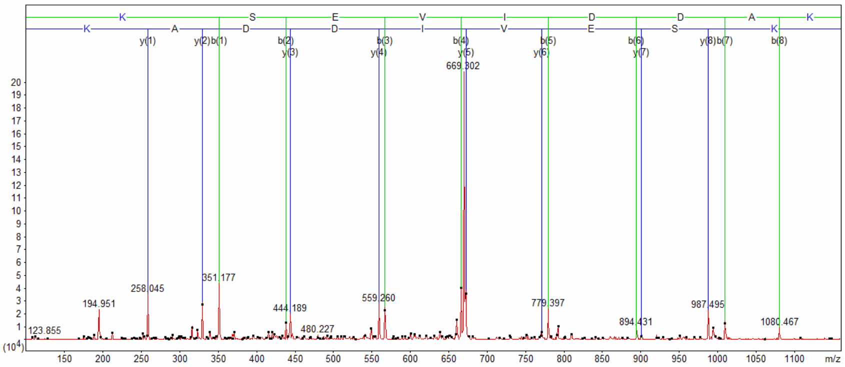

VAS14_19666 (score 57)

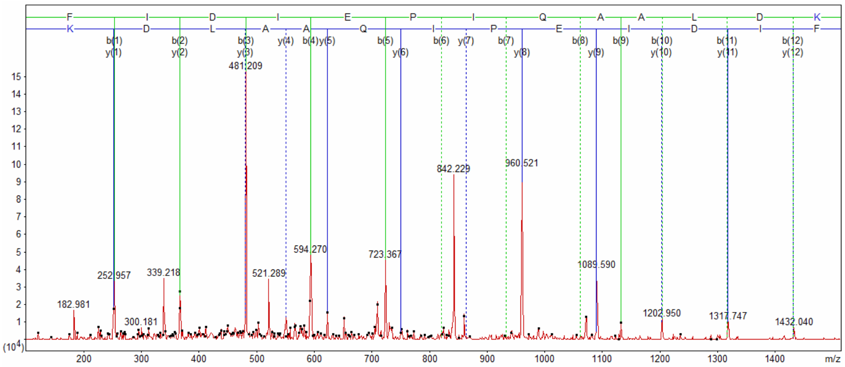

VAS14_02738 (score 55)

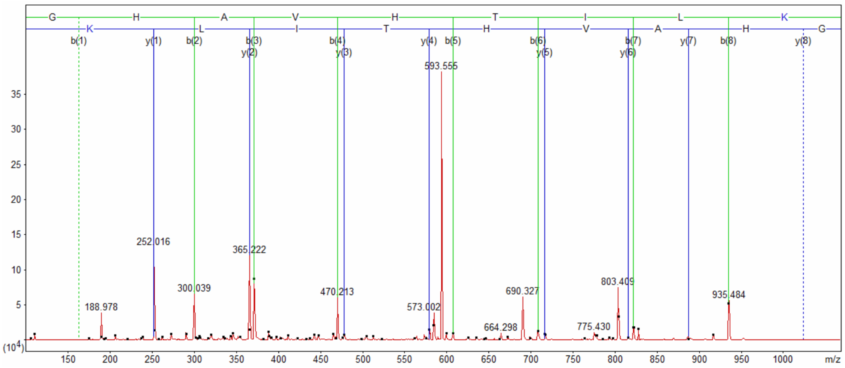

VAS14_ 00641(score 53)

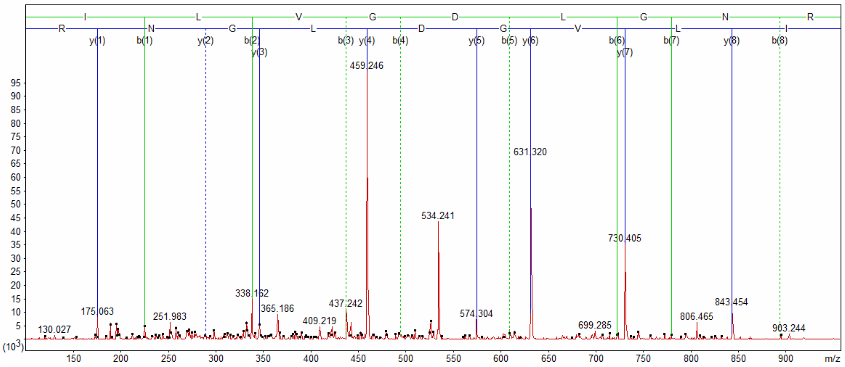

VAS14_19896 (score 49)

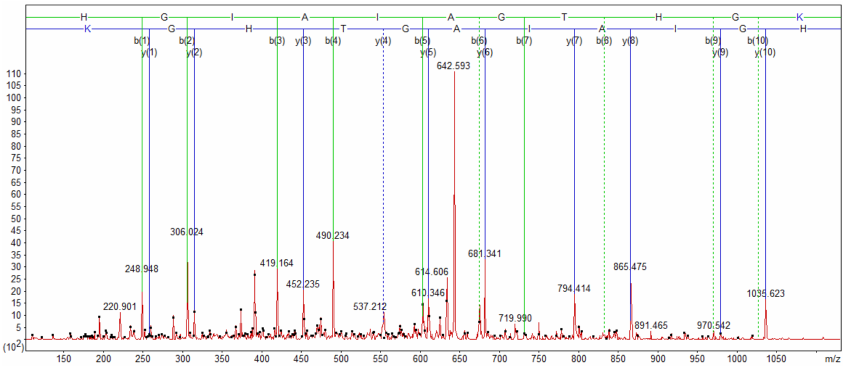

VAS14_03053 (score 47)

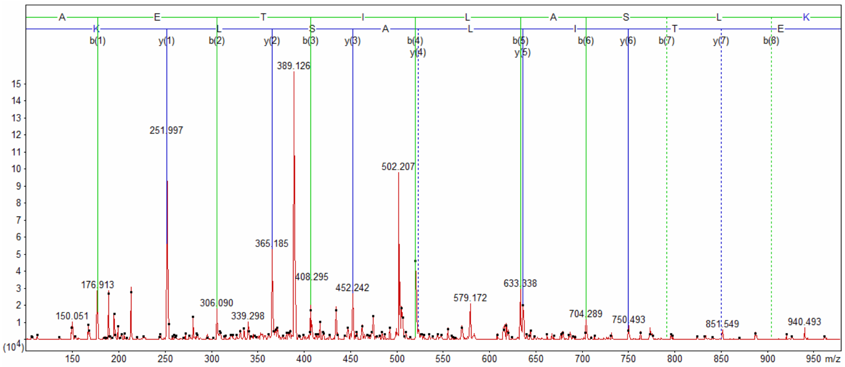

VAS14_07789 (score 46)

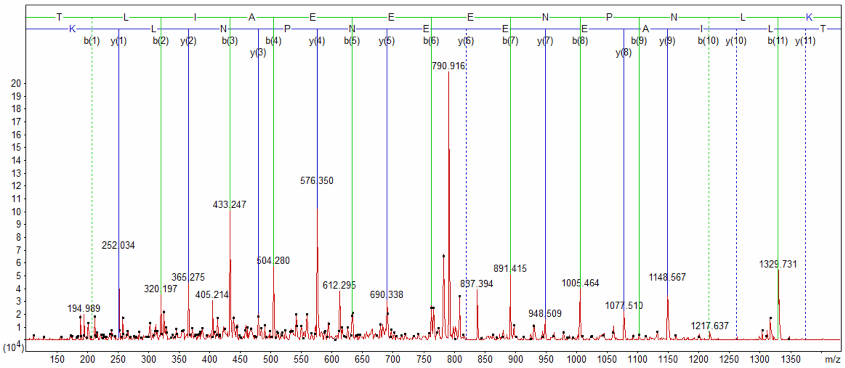

VAS14_05903 (score 41)

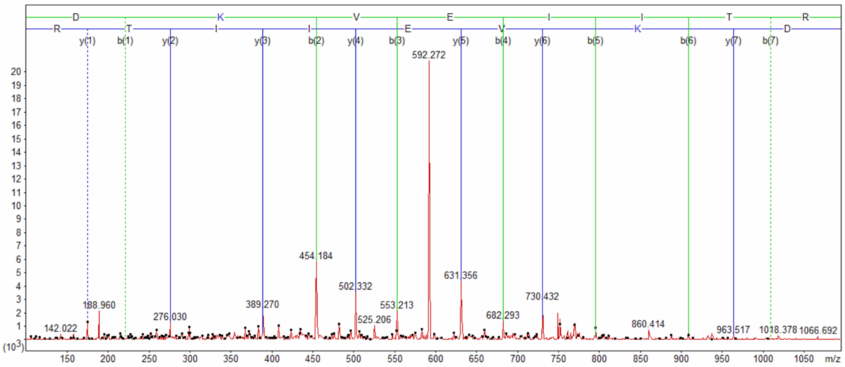

VAS14_07945 (score 40)

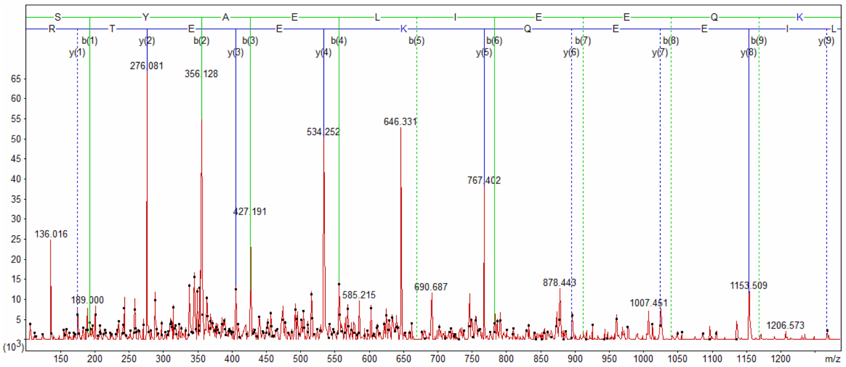

REPLICATE 4

VAS14_07719 (score 82)

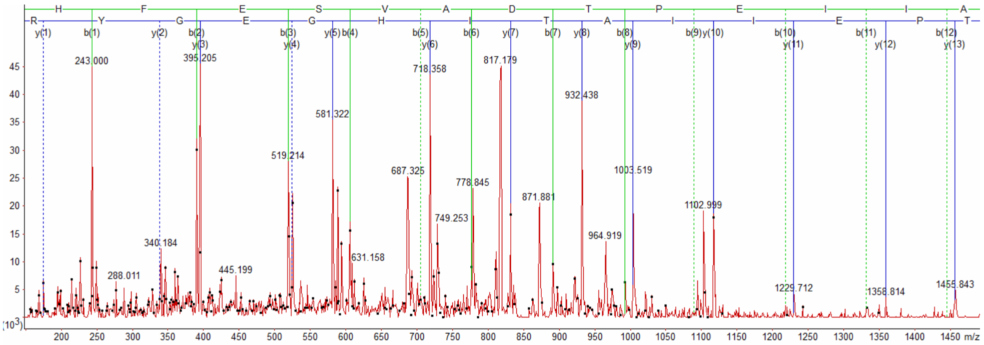

VAS14_04213 (score 78)

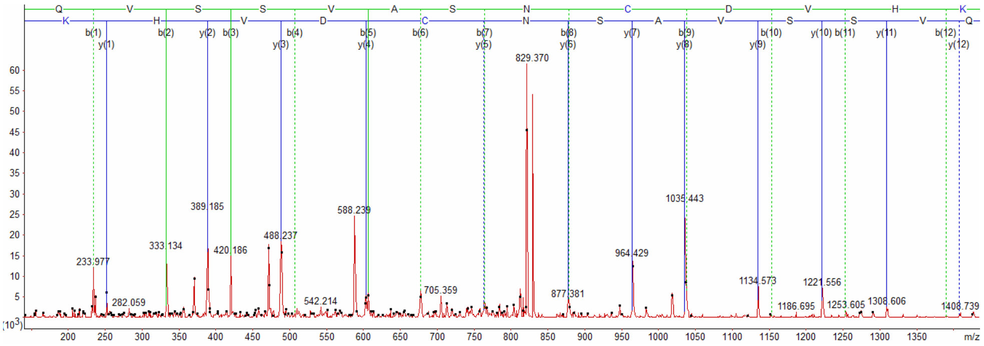

VAS14_21507 (score 73)

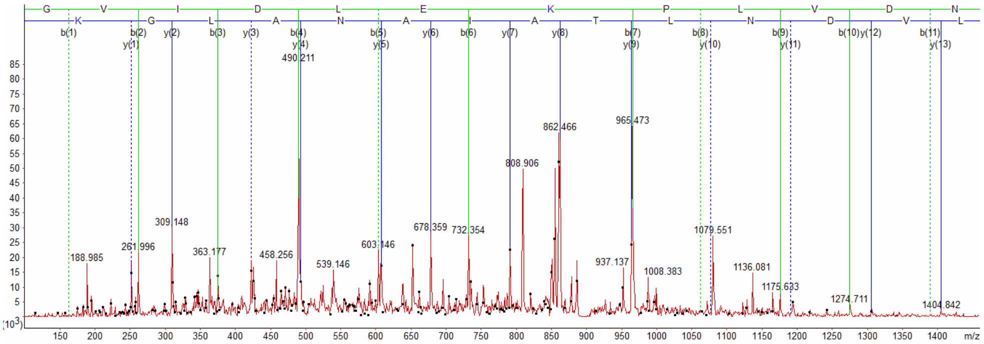

VAS14_ 06353 (score 68)

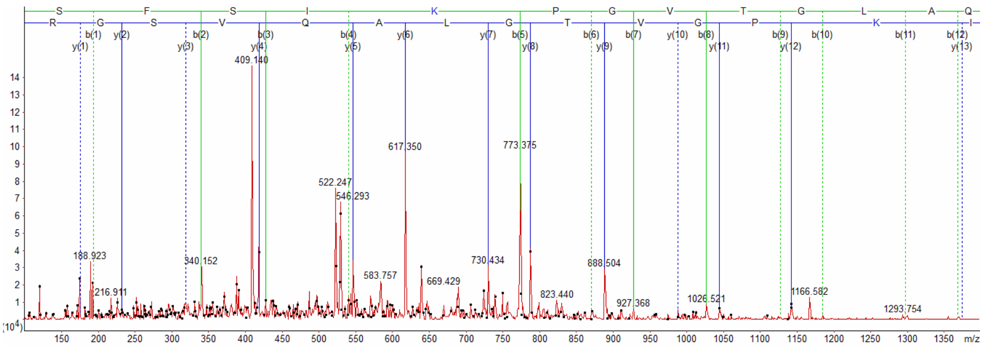

VAS14_21772 (score 67)

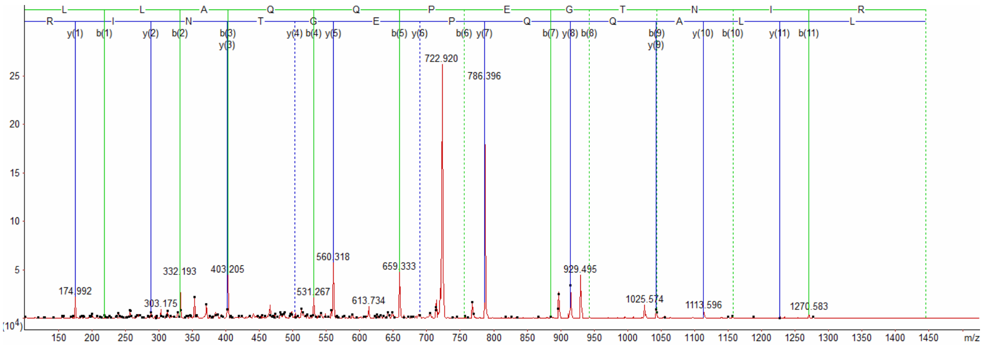

VAS14_21322 (score 65)

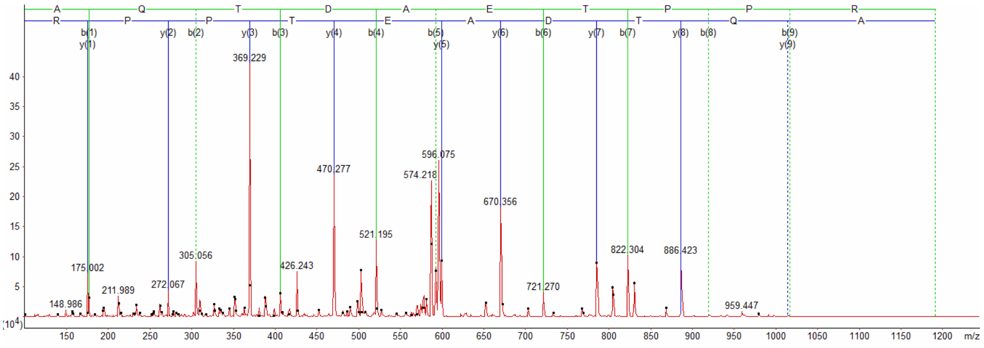

VAS14_04148 (score 65)

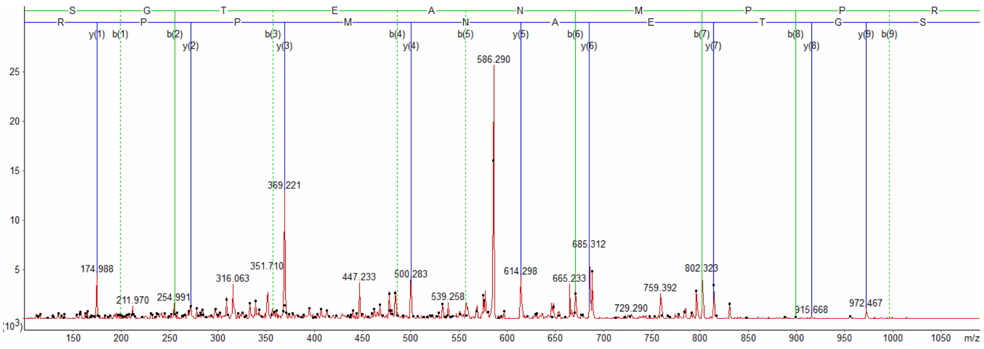

VAS14_17316 (score 65)

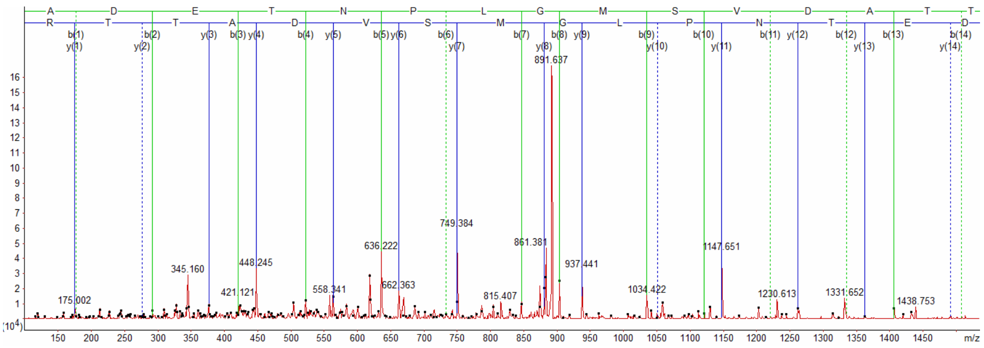

VAS14_ 08335 (score 61)

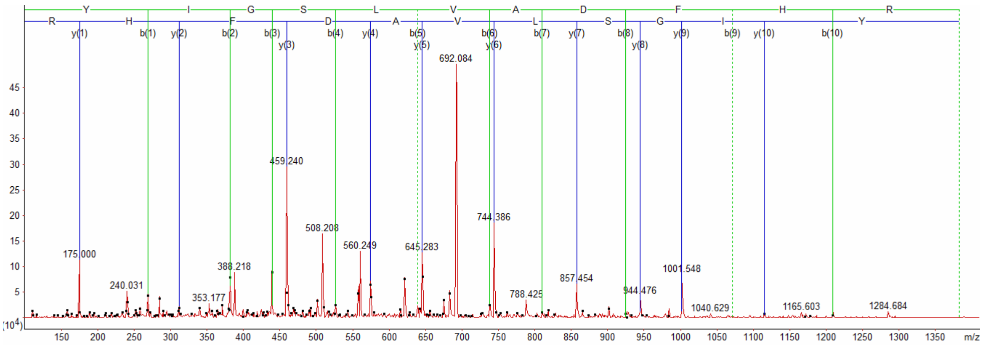

VAS14_18961 (score 60)

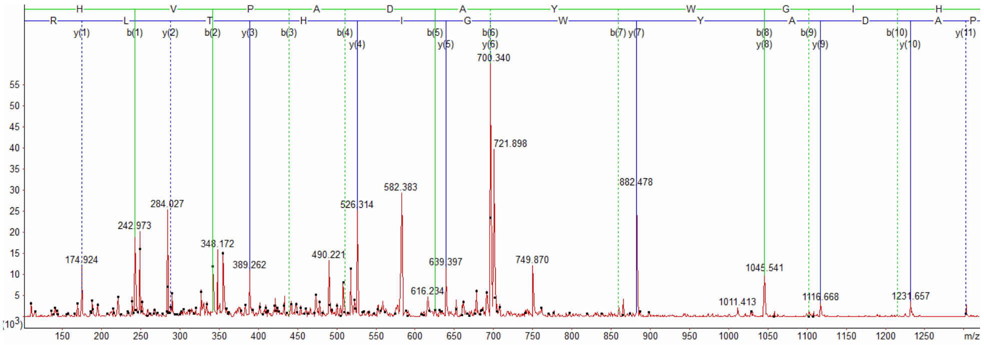

VAS14_05643 (score 57)

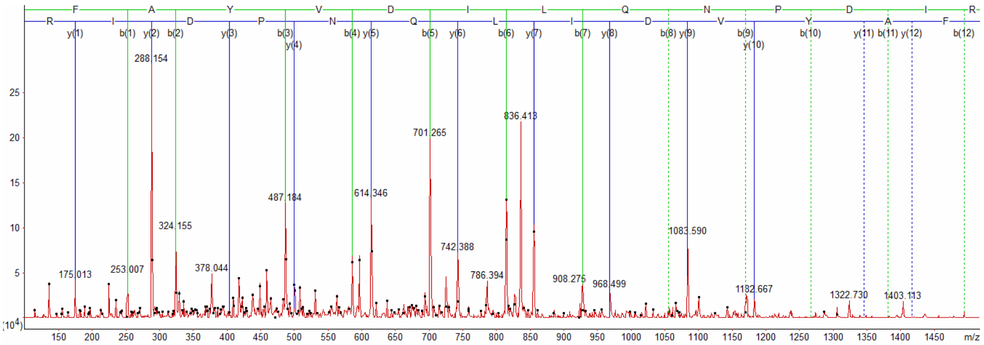

VAS14_21362 (score 57)

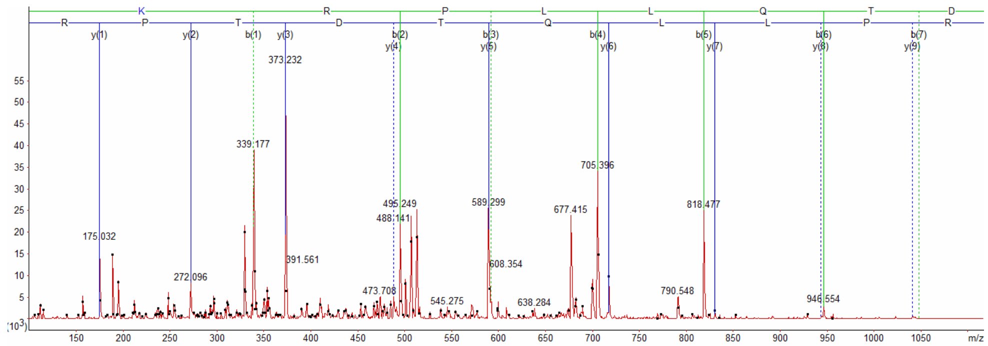

VAS14_22162 (score 56)

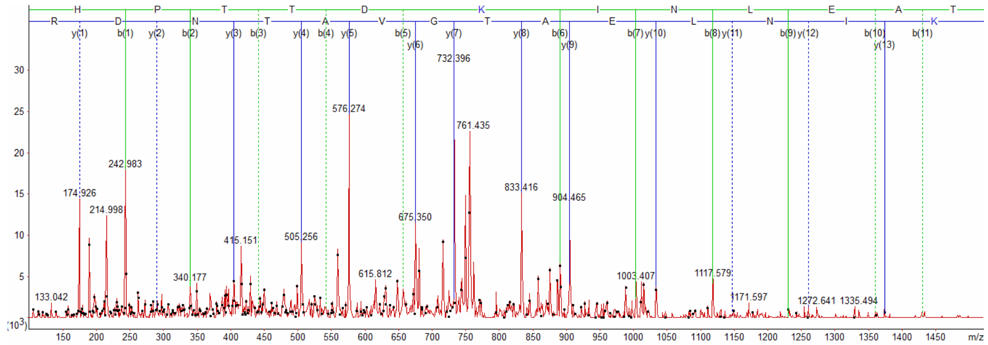

VAS14_04613 (score 56)

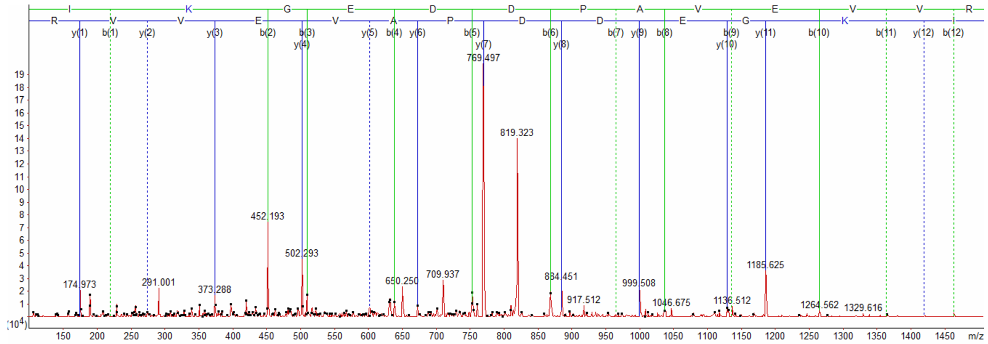

VAS14_17871 (score 55)

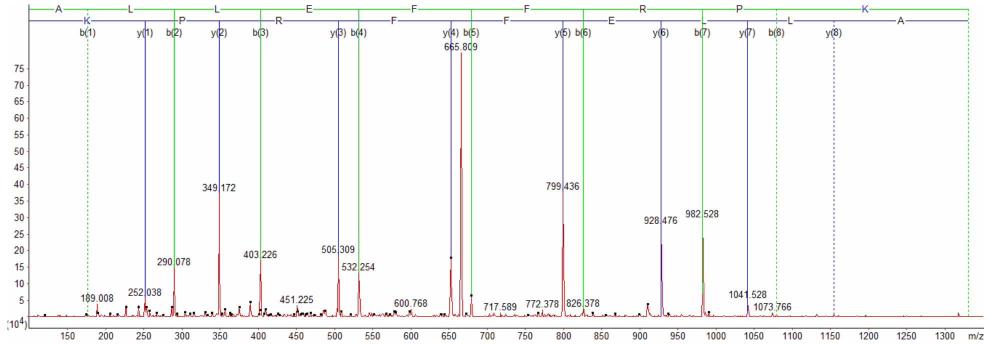

VAS14_02878 (score 54)

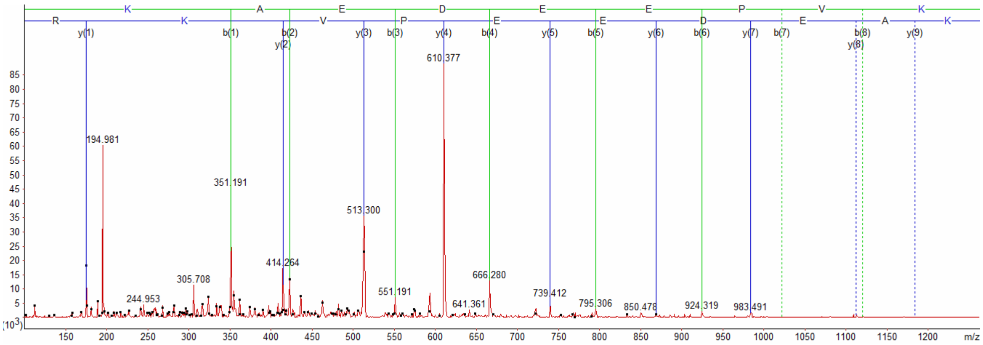

VAS14_06693 (score 53)

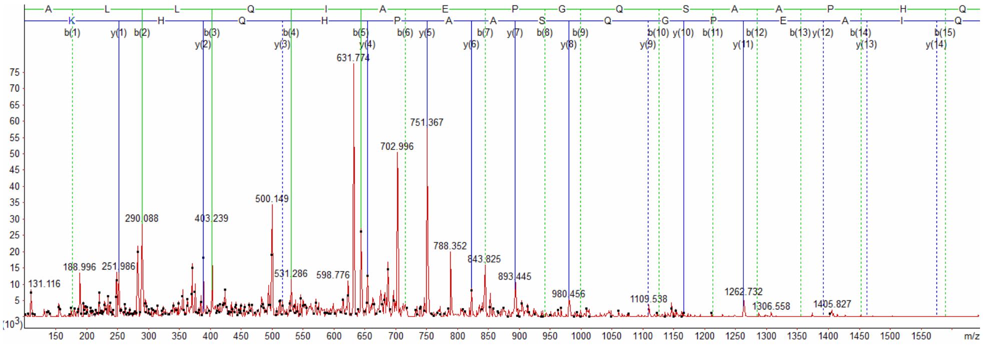

VAS14_22794 (score 54)

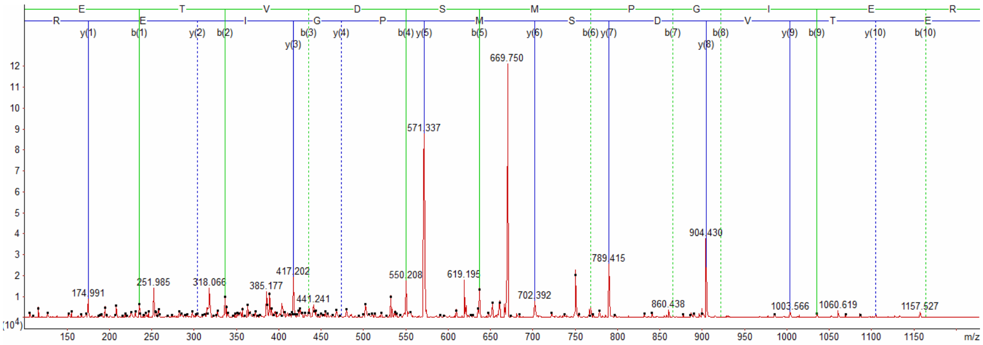

VAS14_09399 (score 50)

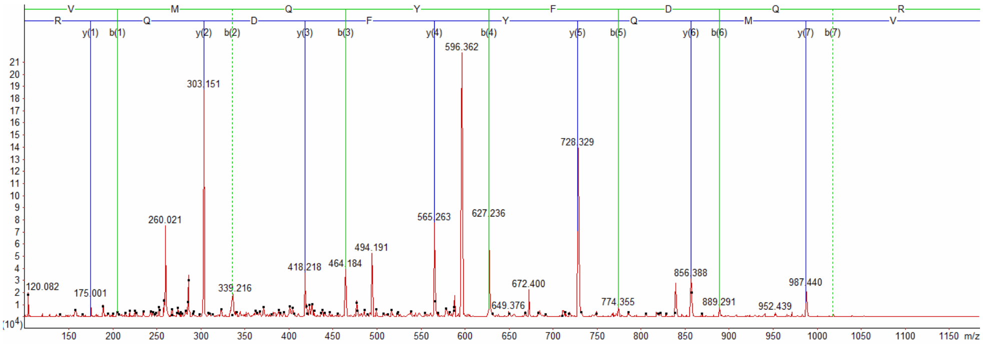

VAS14_ 22854 (score 46)

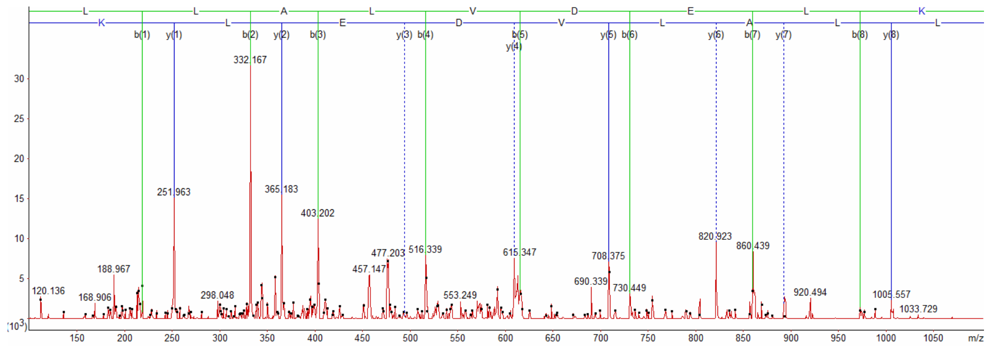

VAS14_07369 (score 46)

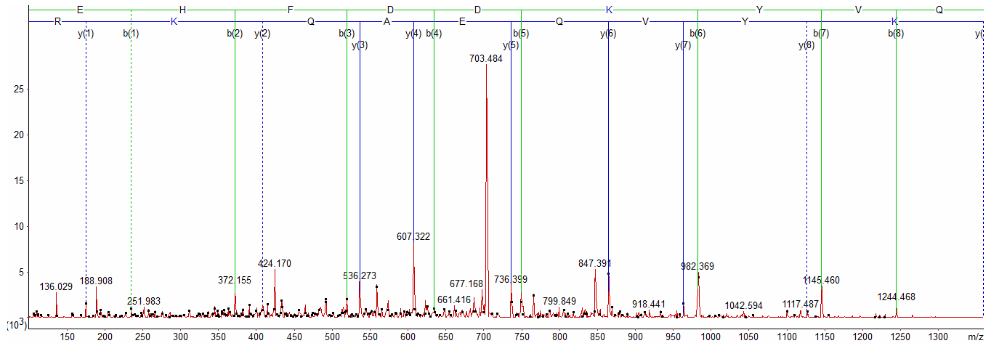

VAS14_18624 (score 46)

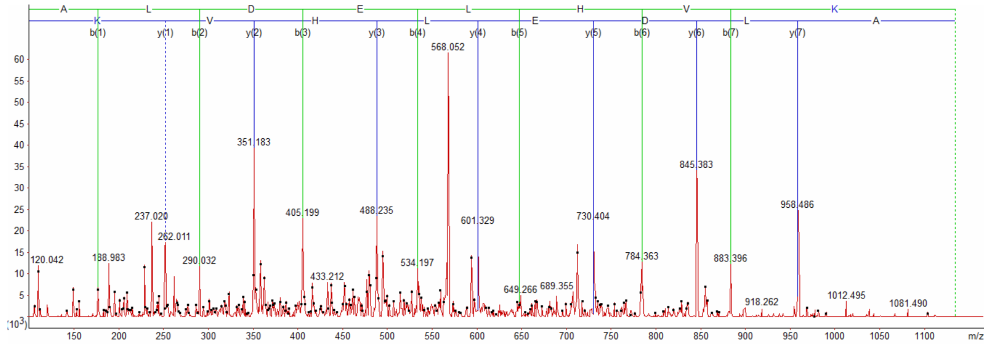

VAS14_03053 (score 46)

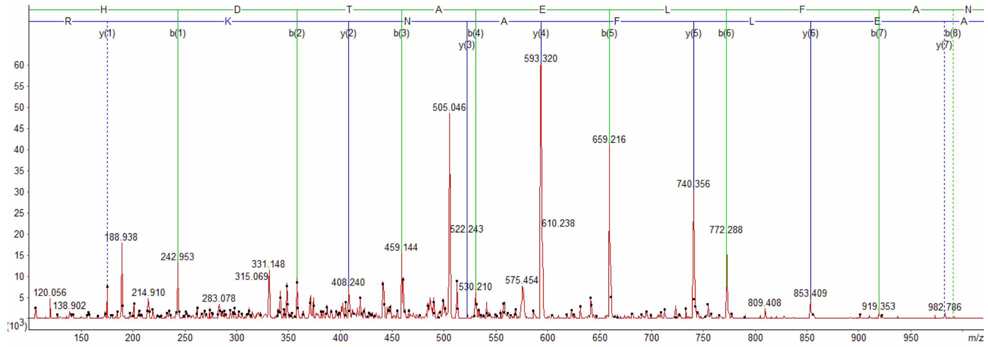

VAS14_15679 (score 45)

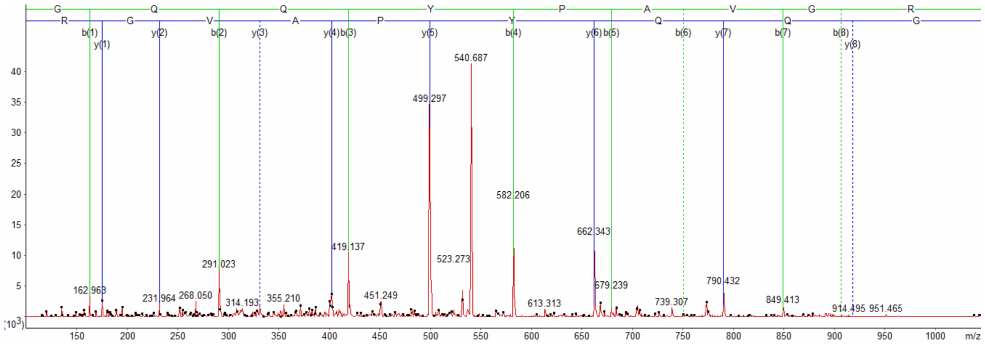

VAS14_02316 (score 43)

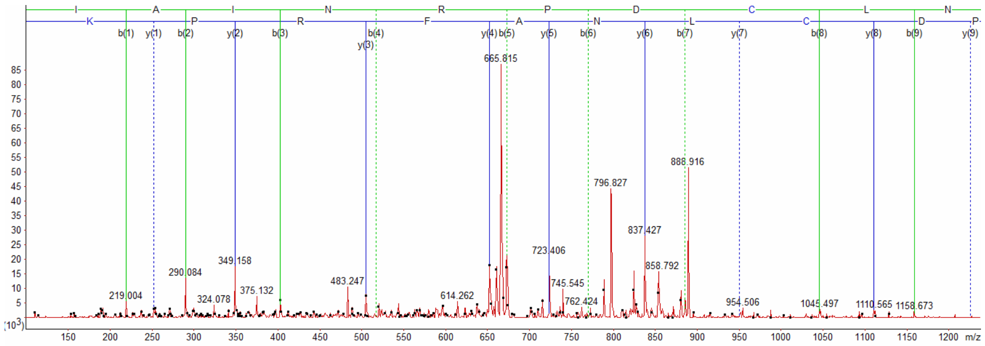

Supplement: Figure S1 — Annotated tandem mass spectra. (PDF) [file pone.0042299.s001.pdf]
