# Supplementary figures and images for: Proteome Analysis of the UVB-Resistant Marine Bacterium Photobacterium angustum S14
Source: PLoS One. 2012 Aug 1;7(8):e42299. doi: 10.1371/journal.pone.0042299 (PMC3411663; doi:10.1371/journal.pone.0042299)

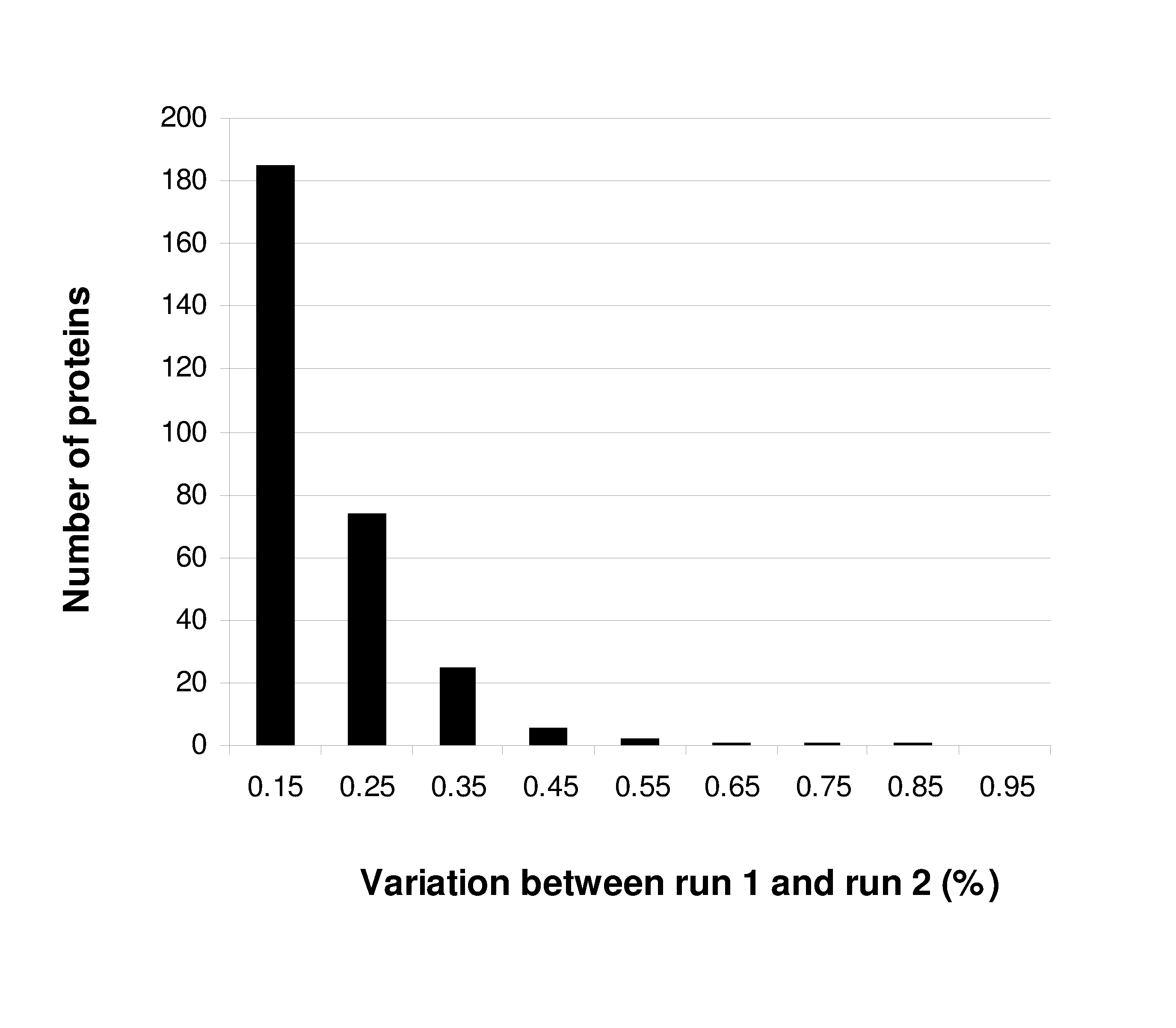

Supplement: Figure S2 — The % variation for the common proteins (295) from the two technical replicates. (TIF) [file pone.0042299.s002.tif]

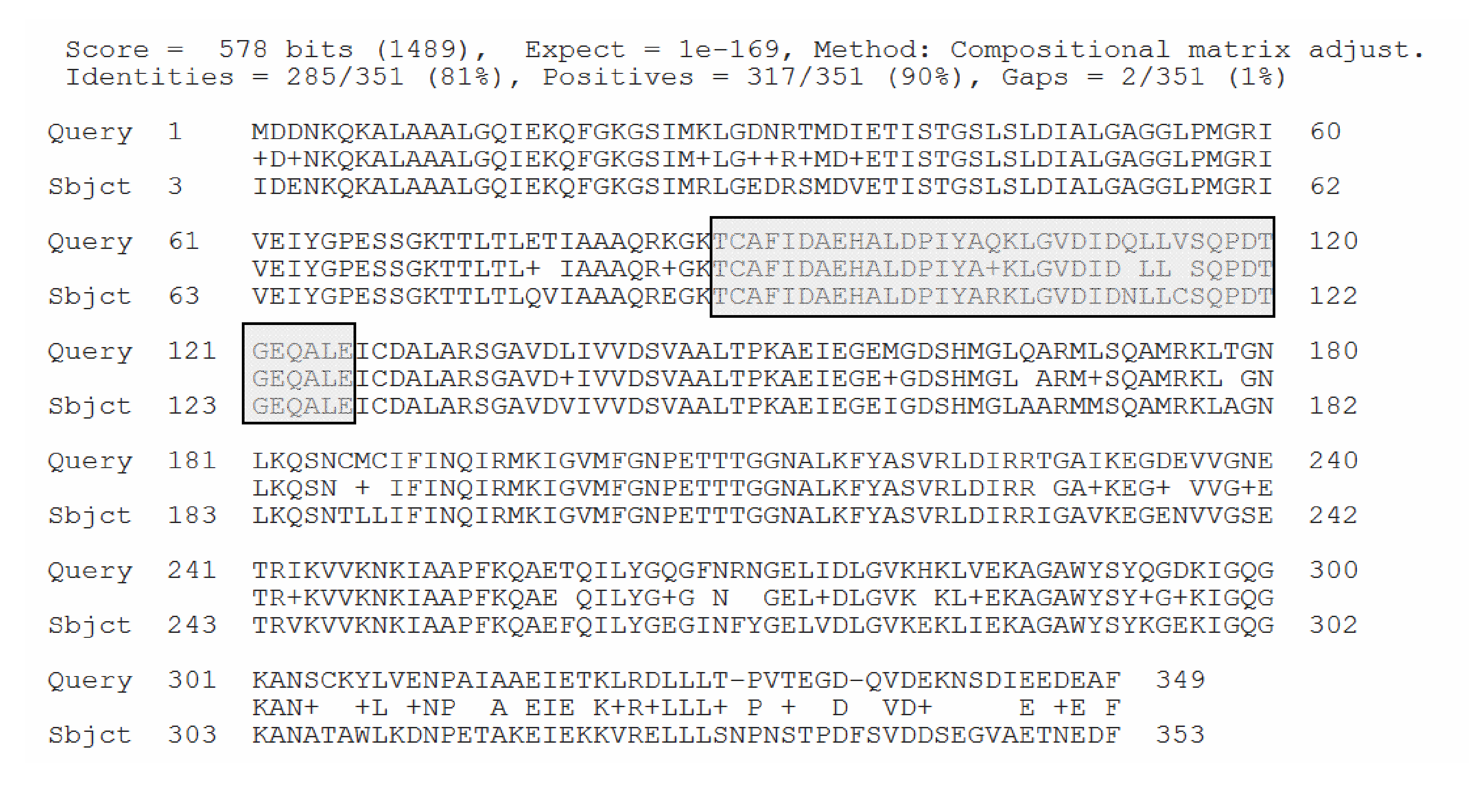

Supplement: Figure S3 — Alignment of the RecA protein sequence from P. angustum with its homolog from E. coli. Boxed residues indicates similar amino acids that are recognized by the primary antibody against RecA. (TIF) [file pone.0042299.s003.tif]

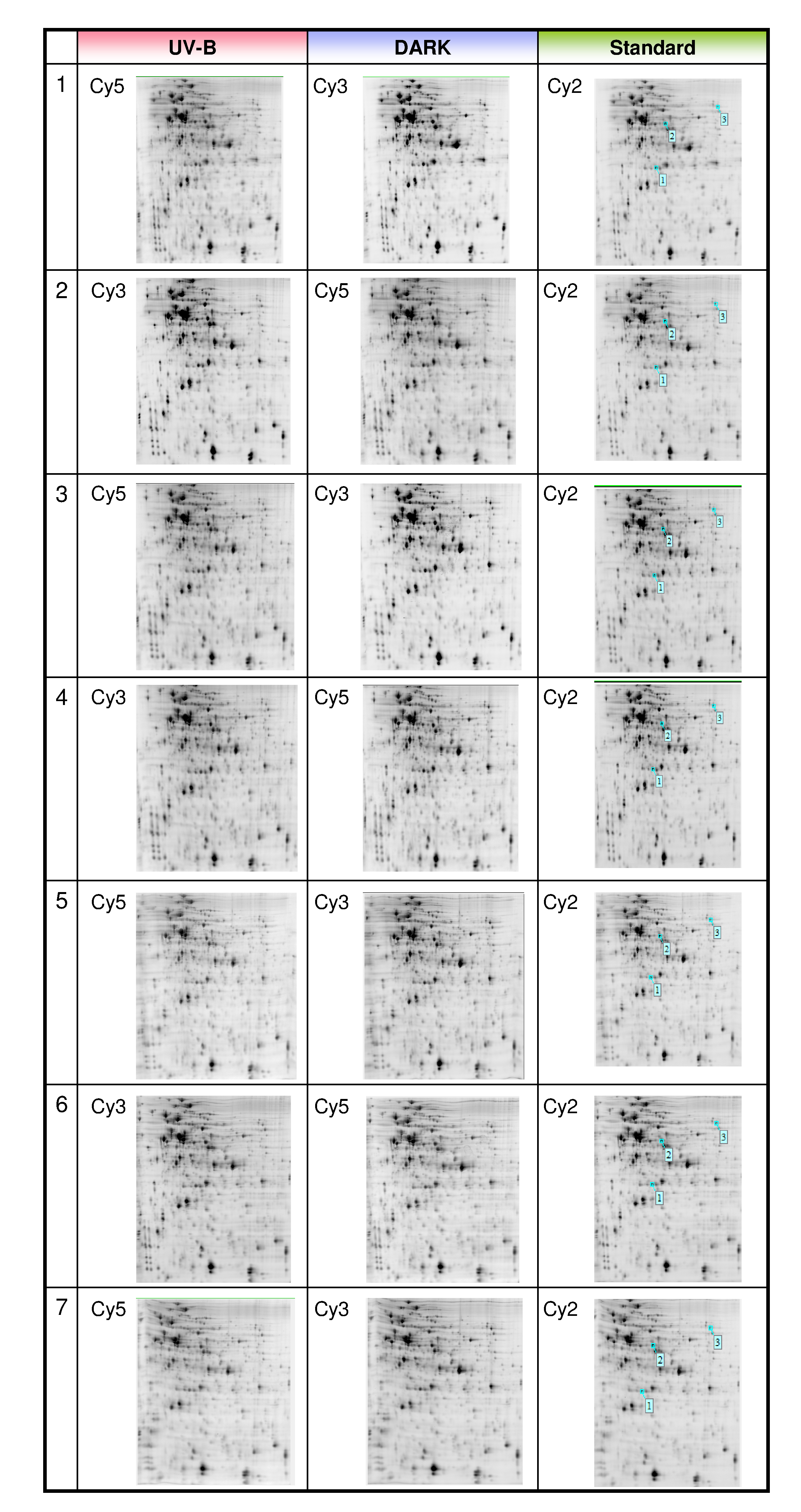

Supplement: Figure S4 — 2D-DIGE experimental workflow from seven replicates. The three landmarks used for gels matching are indicated in blue, for the seven standard gels. (TIF) [file pone.0042299.s004.tif]
